# Supplementary figures and images for: Association of suicidal behavior with exposure to suicide and suicide attempt: A systematic review and multilevel meta-analysis
Source: PLoS Med. 2020 Mar 31;17(3):e1003074. doi: 10.1371/journal.pmed.1003074 (PMC7108695; doi:10.1371/journal.pmed.1003074)

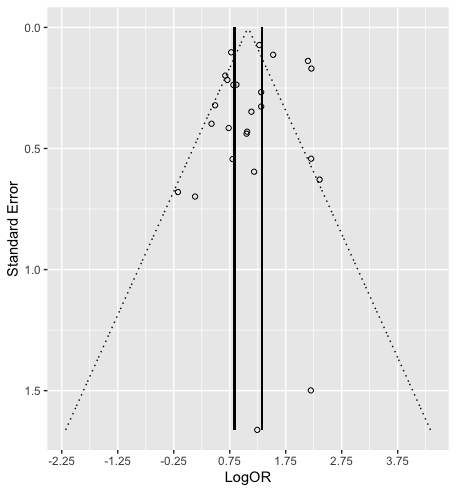

Supplement: S1 Fig — The solid vertical lines indicate the 95% confidence interval around the log odds ratio (LogOR). The dashed lines indicate the summary log odds ratio ± 1.96 × standard error for each of the standard errors on the y-axis. The resulting triangular region indicates the expected location of 95% of studies in the absence of small study effect. (TIF) [file pmed.1003074.s002.tif]

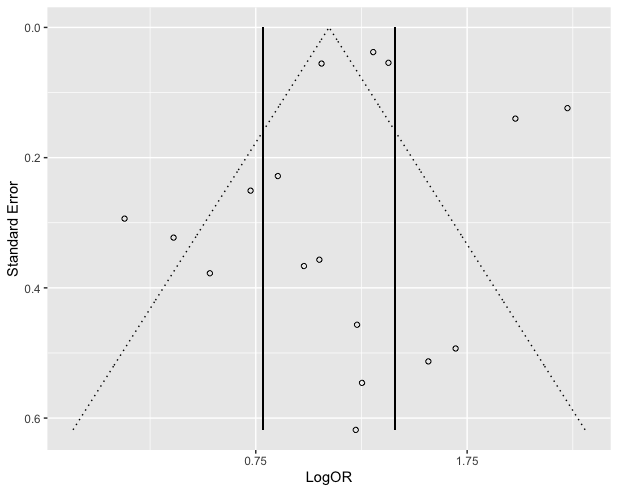

Supplement: S2 Fig — The solid vertical lines indicate the 95% confidence interval around the log odds ratio (LogOR). The dashed lines indicate the summary log odds ratio ± 1.96 × standard error for each of the standard errors on the y-axis. The resulting triangular region indicates the expected location of 95% of studies in the absence of small study effect. (TIFF) [file pmed.1003074.s003.tiff]

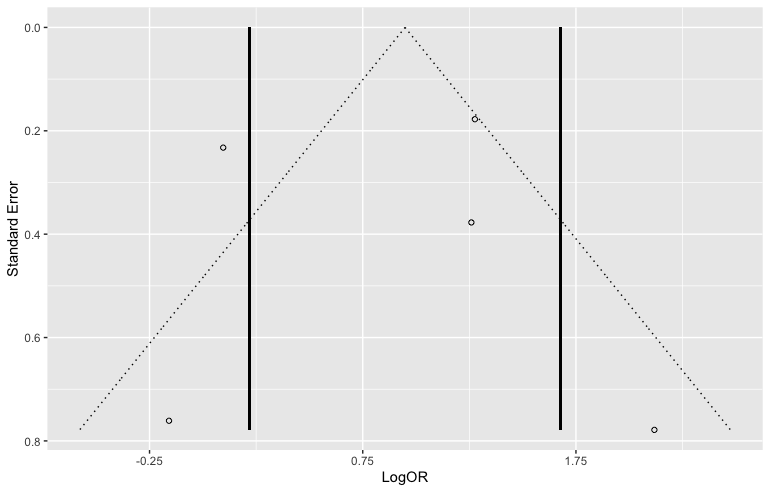

Supplement: S3 Fig — The solid vertical lines indicate the 95% confidence interval around the log odds ratio (LogOR). The dashed lines indicate the summary log odds ratio ± 1.96 × standard error for each of the standard errors on the y-axis. The resulting triangular region indicates the expected location of 95% of studies in the absence of small study effect. (TIFF) [file pmed.1003074.s004.tiff]
