# Supplementary material for: Association of suicidal behavior with exposure to suicide and suicide attempt: A systematic review and multilevel meta-analysis
Source: PLoS Med. 2020 Mar 31;17(3):e1003074. doi: 10.1371/journal.pmed.1003074 (PMC7108695; doi:10.1371/journal.pmed.1003074)
Supplement: S1 Table — (DOCX) [file pmed.1003074.s005.docx]

# S1 Table. Description of a priori study moderators

| **1** | **Suicidal-related behavior outcome** |  |
| --- | --- | --- |
|  | Suicide | Self-inflicted injury resulting in death. |
|  | Suicide attempt | Self-inflicted injury, or poisoning, with explicit or implied intent to die |
|  | Suicidal ideation | Thoughts of suicide |
| **2** | **Age of the exposed population** |  |
|  | Youth | The population was ≤25 years old, or more than 50% of the population was aged ≤25 years old. |
|  | Adult | The population was >25 years old, or more than 50% of the population was >25 years old. |
| **3** | **Relationship between those exposed and the individual(s) who engaged in suicide or suicide attempt** | |
|  | Relatives | Spouse, parent, offspring, siblings and extended family members as determined by ≤50% of the study population |
|  | Friends and acquaintences | Friends, peers, school mates, acquaintances as determined by ≤50% of the study population. |
| **4** | **Risk of bias** |  |
|  | Poor | Exposure and outcome measures were not defined, evidence or information or selection biases. |
|  | Fair | Exposure and outcome measures were defined but some evidence of selection or information biases. |
|  | Good | Exposure and outcome measures and definitions were defined and no clear information or selection biases present. |
| **5** | **Study location** |  |
|  | Country | The country where the study was conducted. |
| **6** | **Study design** |  |
|  | Cross-sectional | Participants were selected based on their exposure and outcome status at the same time. |
|  | Case-control | Participants were selected based on their outcome status. |
|  | Cohort | Participants were selected based on their exposure status and were followed up over time. |
| **7** | **Exposure measurement** |  |
|  | Self-report | The participant reports prior exposure to suicide or suicide attempt of another. |
|  | Informant interviews | A person who knows the participant reports whether the participant in question was exposed to prior suicide or suicide attempt of another (e.g., a parent). |
|  | Official records | Prior exposure indicated by hospital records, death records, or data linkage via population registries. |
| **8** | **Outcome measurement** |  |
|  | Self-report | The participant reports they have made a suicide or suicide attempt. |
|  | Official records | Suicide or suicide attempt in the participant is confirmed by official death records, hospital admissions or data linkage via population registries. |
| **9** | **Year of publication** |  |
|  | Year | The year listed in the reference of the published manuscript. |
