# Supplementary material for: Association of suicidal behavior with exposure to suicide and suicide attempt: A systematic review and multilevel meta-analysis
Source: PLoS Med. 2020 Mar 31;17(3):e1003074. doi: 10.1371/journal.pmed.1003074 (PMC7108695; doi:10.1371/journal.pmed.1003074)
Supplement: S2 Table — (DOCX) [file pmed.1003074.s006.docx]

# S2 Table. Excluded overlapping studies

| **Overlapping study- excluded from analysis** | **Reason for exclusion** | **Included study** | **Reason for including study** |
| --- | --- | --- | --- |
| Ahmadi A, Mohammadi R, Schwebel DC, Yeganeh N, Soroush A, Bazargan-Hejazi S. Familial risk factors for self-immolation: A case-control study. *Journal of Women's Health.* 2009;18(7):1025-1031. | Excluded because it’s a sub sample Ahmadi et al. 2015 study a, N= 30 cases; N= 30 controls | Ahmadi A, Mohammadi R, Almasi A, et al. A case-control study of psychosocial risk and protective factors of self-immolation in Iran. Burns. 2015;41(2):386-393. Study a | Author indicated this is the main study with complete sample of N=151 cases and N=302 controls. The study also reports outcomes for both exposure behaviours (suicide and self-harm/attempt) which is not the case in the remaining two studies. |
| Ahmadi A, Schwebel DC, Bazargan-Hejazi S, Taliee K, Karim H, Mohammadi R. Self-immolation and its adverse life-events risk factors: results from an Iranian population. *Journal of injury & violence research.* 2015;7(1):13-18. | Excluded because it’s a sub sample Ahmadi et al. 2015 study a, N= 30 cases; N= 30 controls | Ahmadi et al. 2015 study a |  |
| Brent DA, Oquendo M, Birmaher B, et al. Familial pathways to early-onset suicide attempt: Risk for suicidal behavior in offspring of mood-disordered suicide attempters. *Archives of General Psychiatry.* 2002;59(9):801-807. | Excluded because it’s a sub sample Brent et al. 2015. Offspring of parents who attempted suicide, Self-harm/suicide attempt. The sample consisted of N=449 offspring of N=255 pro-bands with history of mood disorders. | Brent DA, Melhem NM, Oquendo M, et al. Familial pathways to early-onset suicide attempt: A 5.6-year prospective study. JAMA Psychiatry. 2015;72(2):160-168. | The sample consisted of 701 offspring of 334 pro-bands with mood disorders. Pro-bands were clinically referred to the Western Psychiatric Institute or the New York State Psychiatric Institute from Jul 1997- Sep 2005, and followed through to June 2012, encompassing a 5.6-year follow-up period. |
| Melhem NM, Brent DA, Ziegler M, et al. Familial pathways to early-onset suicidal behavior: Familial and individual antecedents of suicidal behavior. *American Journal of Psychiatry.* 2007;164(9):1364-1370. | Excluded because it’s a sub sample of Brent et al. 2015. Offspring of parents who attempted suicide, Self-harm/suicide attempt. The sample consisted of N=365 offspring of N=203 pro-bands with a history of mood disorders. | Brent et al. 2015 |  |
| Cerel J, Van De Venne JG, Moore MM, Maple MJ, Flaherty C, Brown MM. Veteran exposure to suicide: Prevalence and correlates. *Journal of Affective Disorders.* 2015;179:82-87. | Excluded because it's a sub sample of Cerel et al. 2016. The sample consisted N=916 people from the general population exposed or unexposed to suicide of a friend or family member. | Cerel J, Maple M, van de Venne J, Moore M, Flaherty C, Brown M. Exposure to suicide in the community: Prevalence and correlates in one U.S. State. Public Health Reports. 2016;131(1):100-107. | The sample consisted of N=1703 people from the general population exposed or unexposed to suicide of a friend or family member. |
| Garcia-Valencia J, Palacio-Acosta C, Diago J, et al. Adverse life events and suicide: A case-control study of psychological autopsy in Medellin, Colombia. *Eventos vitales adversos y suicidio: Un estudio de autopsia psicologica en Medellin, Colombia.* 2008;37(1):11-28. | Excluded because it’s the same study as Palacio et al. 2007 but published in Spanish | Palacio C, Garcia J, Diago J, et al. Identification of Suicide Risk Factors in Medellin, Colombia: A Case-Control Study of Psychological Autopsy in a Developing Country. Archives of Suicide Research. 2007;11(3):297-308. | Same article but Published in English. |
| Brent DA, Perper JA, Moritz G, Liotus L, Schweers J, Canobbio R. Major depression or uncomplicated bereavement? A follow-up of youth exposed to suicide. Journal of the American Academy of Child and Adolescent Psychiatry. 1994;33(2):231-239. | Excluded because it’s a sub sample of Brent et al. 1996 study from an earlier time point. The sample consisted of N=146 friends of a suicide descendent and N=146 unexposed community controls, followed up at 12-18 months post baseline interview. | Brent DA, Moritz G, Bridge J, Perper J, Canobbio R. Long-term impact of exposure to suicide: A three-year controlled follow-up. Journal of the American Academy of Child and Adolescent Psychiatry. 1996;35(5):646. (study a) | The sample consisted of N=166 friends of adolescent suicide descendants and N=175 unexposed community controls, followed up at 36 months post baseline interview. The sample included initial participants and follow-up with those who refused throughout the trial. The exposure occurred between Dec 1988-March 1991. |
| Brent DA, Perper JA, Moritz G, et al. Psychiatric impact of the loss of an adolescent sibling to suicide. *Journal of Affective Disorders.* 1993;28(4):249-256. | Excluded because it’s an earlier time point of Brent 1996 study b. The sample consisted of N=25 siblings of N=20 suicide descendants and 25 matched community controls. | Brent DA, Moritz G, Bridge J, Perper J, Canobbio R. The Impact of Adolescent Suicide on Siblings and Parents: A Longitudinal Follow-Up. Suicide and Life-Threatening Behavior. 1996;26(3):253-259. (study b) | The initial sample consisted of N=25 siblings of N=20 suicide descendants and N=25 matched community controls, followed up at 37.6 months |
| C. C. Cheng et al., Risk of adolescent offspring's completed suicide increases with prior history of their same-sex parents' death by suicide. Psychol Med 44, 1845-54 (2014). | Excluded because superseded by longer timepoint in Lee et al. 2018 Data linkage study using Taiwan Births and Deaths registry.  Exposure: Suicide of parent Outcome: Suicide between 1997-2007 | K. Y. Lee et al., Age at Exposure to Parental Suicide and the Subsequent Risk of Suicide in Young People. Crisis: Journal of Crisis Intervention & Suicide 39, 27-36 (2018). | Data linkage study using Taiwan Births and Deaths registry with suicide occurring between 1978-2009 |
| Abrutyn S, Mueller AS. Are Suicidal Behaviors Contagious in Adolescence? Using Longitudinal Data to Examine Suicide Suggestion. American Sociological Review. 2014;79(2):211-227. | Excluded because superseded by longer timepoints in Thompson et al. 2011. National Longitudinal Study of Adolescent Health. Measured subsequent suicidal thoughts and attempts at wave III based on exposure to suicide attempt of family or friend at wave II (7 years earlier). | Thompson MP, Light LS. Examining Gender Differences in Risk Factors for Suicide Attempts Made 1 and 7 Years Later in a Nationally Representative Sample. Journal of Adolescent Health. 2011;48(4):391-397. | National Longitudinal Study of Adolescent Health. Included because it provides outcome measures for each of the exposure subgroups over an equivocal period of time (7 years). Subsequent suicide attempt reported at 7 years follow up (wave III) following exposures reported in wave I or II: 1) Friend completed suicide; 2) Friend attempted suicide; 3) Family completed suicide; 4) Family attempted suicide |
| Mueller AS, Abrutyn S, Stockton C. Can Social Ties Be Harmful? Examining the Spread of Suicide in Early Adulthood. Sociological Perspectives. 2015;58(2):204-222. | National Longitudinal Study of Adolescent Health.Subsequent suicide attempt reported at wave IV following exposures reported in Wave III (exposure to suicide or suicide attempt in the past 12 months). Data was not entered because the length of follow-up between measured exposure and outcome was equivocal to Thompson 2011, and the authors combined subgroups (friend and family) and exposure behaviour (suicide and suicide attempt) into one outcome. | Thompson et al. 2011 |  |
| Hill RM, Oosterhoff B, Kaplow JB. Prospective identification of adolescent suicide ideation using classification tree analysis: Models for community-based screening. *Journal of Consulting and Clinical Psychology.* 2017;85(7):702. | National Longitudinal Study of Adolescent Health. Data from wave I & II (1 year follow-up) Data not entered due to limited follow-up period. | Thompson et al. 2011 |  |
| Randall JR, Nickel NC, Colman I. Contagion from peer suicidal behavior in a representative sample of American adolescents. *Journal of Affective Disorders.* 2015;186:219-225. | National Longitudinal Study of Adolescent Health. Data from wave I & II (1 year follow-up). Data not entered due to limited follow-up period. | Thompson et al. 2011 |  |
| Mueller AS, Abrutyn S. Suicidal disclosures among friends: using social network data to understand suicide contagion. Journal of Health & Social Behavior. 2015;56(1):131-148. | National Longitudinal Study of Adolescent Health. Data from wave I & II (1 year follow-up). Data not entered due to limited follow-up period. | Thompson et al. 2011 |  |
| Nanayakkara S, Misch D, Chang L, Henry D. Depression and exposure to suicide predict suicide attempt. *Depression and Anxiety.* 2013;30(10):991-996. | National Longitudinal Study of Adolescent Health. Data from wave I & II (1-year follow-up). Data not entered due to limited follow-up period. | Thompson et al. 2011 |  |
| Ali MM, Dwyer DS, Rizzo JA. The social contagion effect of suicidal behavior in adolescents: Does it really exist? *Journal of Mental Health Policy and Economics.* 2011;14(1):3-12. | National Longitudinal Study of Adolescent Health.Data from wave I & II (1-year follow-up) | Thompson et al. 2011 |  |
| Zamora-Kapoor A, Nelson LA, Barbosa-Leiker C, Comtois KA, Walker LR, Buchwald DS. Suicidal ideation in American Indian/Alaska Native and White adolescents: The role of social isolation, exposure to suicide, and overweight. *American Indian and Alaska native mental health research (Online).* 2016;23(4):86-100. | National Longitudinal Study of Adolescent Health. Data from wave I (baseline). | Thompson et al. 2011 |  |
| Cerel J, Roberts TA. Suicidal behavior in the family and adolescent risk behavior. *Journal of Adolescent Health.* 2005;36(4):352. | National Longitudinal Study of Adolescent Health. Data from wave I (baseline) | Thompson et al. 2011 |  |
| Cerel J, Roberts TA, Nilsen WJ. Peer suicidal behavior and adolescent risk behavior. *Journal of Nervous and Mental Disease.* 2005;193(4):237-243. | National Longitudinal Study of Adolescent Health. Data from wave I (baseline). | Thompson et al. 2011 |  |
| R. X. Liu, Vulnerability to Friends’ Suicide Influence: The Moderating Effects of Gender and Adolescent Depression. Journal of Youth and Adolescence 35, 454 (2006). | National Longitudinal Study of Adolescent Health. Data from wave I (baseline). | Thompson et al. 2011 |  |
| M. Asberg et al., Family history of suicide among suicide victims. American Journal of Psychiatry 160, 1525-6 (2003). | Sweden population registry-based study Exposure to family history of suicide Exposure: Suicide in relatives Outcome: Suicide between 1963-1997 N=62118 | Tidemalm, D., Runeson, B., Waern, M., Frisell, T., Carlstrom, E., Lichtenstein, P. & Langstrom, N. Familial clustering of suicide risk: a total population study of 11.4 million individuals', Psychological Medicine. 2011; 41 (12), 2527-34. | Sweden population registry-based study. Broad definition of relative and encompassing all suicide deaths during the study period.  Exposure: Suicide in relatives Outcome: Suicide between 1957-2003  N=7,969,645 (suicide=83,951) |
| E. Rubenowitz et al., Life events and psychosocial factors in elderly suicides--a case-control study. Psychological medicine 31, 1193-202 (2001). | Psychological autopsy study but cases identified from the population register in Sweden.  Exposure: Suicide in relatives Outcome: Suicide between 1994-1996 N=238 | Tidemalm et al. 2012 |  |
| M. B. Guldin et al., Incidence of suicide among persons who had a parent who died during their childhood a population-based cohort study. JAMA Psychiatry 72, 1227-34 (2015). | Population based study based on three Scandinavian countries. Sweden, Denmark, Norway and specific to childhood exposure. | Tidemalm et al. 2013 |  |
| Agerbo E. Risk of suicide and spouse's psychiatric illness or suicide: nested case-control study. *British Medical Journal.* 2003;327(7422):1025-1026. | Denmark population registry-based study. Exposure: Suicide of spouse/relative Outcome: Suicide between 1982- 1997 N=300,403 (suicide =9011) | Agerbo E, Mortensen PB, Qin P. Suicide risk in relation to socioeconomic, demographic, psychiatric, and familial factors: a national register-based study of all suicides in Denmark, 1981-1997. *American Journal of Psychiatry.* 2003;160(4):765-772. | Denmark population registry-based study. Broad definition of relative and encompassing all suicide deaths during the study period.  Exposure: Suicide of relative Outcome: Suicide between 1981-1997 N=444,297 (suicide=21,169) |
| Qin P, Mortensen PB. The impact of parental status on the risk of completed suicide. *Archives of general psychiatry.* 2003;60(8):797-802. | Denmark population registry-based study. Exposure: Suicide of parent Outcome: Suicide between 1981-1997 N=390831 (suicide=18611) | Agerbo et al. 2003 |  |
| Qin, P., Agerbo, E., & Mortensen, P. B. (2002). Suicide risk in relation to family history of completed suicide and psychiatric disorders: a nested case-control study based on longitudinal registers. Lancet, 12, 1126-1130. | Denmark population registry-based study. Exposure: Suicide relative Outcome: Suicide between 1981-1997 N=253,679 (suicide=12897) | Agerbo et al. 2003 |  |
| Agerbo E, Nordentoft M, Mortensen PB. Familial, psychiatric, and socioeconomic risk factors for suicide in young people: Nested case-control study. *British Medical Journal.* 2002;325(7355):74. | Denmark population registry-based study. Exposure: Suicide of sibling Outcome: Suicide between 1981-1997 N=25296 | Agerbo et al. 2003 |  |
| Sorensen HJ, Mortensen EL, Wang AG, Juel K, Silverton L, Mednick SA. Suicide and mental illness in parents and risk of suicide in offspring: a birth cohort study. *Social psychiatry and psychiatric epidemiology.* 2009;44(9):748-751. | The Copenhagen Perinatal Cohort. Suicide cases identified from Denmark population registry system.  Exposure: Suicide of parent Outcome: Suicide between 1969-1996 N= 7279 | Agerbo et al. 2003 |  |
| Petersen L, Sorensen TIA, Andersen PK, Mortensen PB, Hawton K. Genetic and familial environmental effects on suicide attempts: A study of Danish adoptees and their biological and adoptive siblings. *Journal of Affective Disorders.* 2014;155:273-277. | Denmark population registry-based study. Exposure: Suicide of sibling Outcome: Suicide between 1977-2010 N=3202 | Agerbo et al. 2003 |  |
| L. Petersen et al., Genetic and familial environmental effects on suicide--an adoption study of siblings. PloS one 8, e77973 (2013). | Denmark population registry-based study.Exposure: Suicide of adopted siblingOutcome: Suicide between date of adoption to 2009N= 6172 | Agerbo et al. 2003 |  |
