# Supplementary material for: Association of suicidal behavior with exposure to suicide and suicide attempt: A systematic review and multilevel meta-analysis
Source: PLoS Med. 2020 Mar 31;17(3):e1003074. doi: 10.1371/journal.pmed.1003074 (PMC7108695; doi:10.1371/journal.pmed.1003074)
Supplement: S3 Table — (DOCX) [file pmed.1003074.s007.docx]

# S3 Table. Risk of bias

## Case control studies

| 1. Were the definitions, inclusion and exclusion criteria, algorithms or processes used to identify or select cases and controls valid, reliable, and implemented consistently across all study participants? 2. Implemented consistently 3. Valid/reliable 4. Were the cases clearly defined and differentiated from controls? 5. If less than 100 percent of eligible cases and/or controls were selected for the study, were the cases and/or controls randomly selected from those eligible? 6. Was there use of concurrent controls? 7. Were the investigators able to confirm that the exposure/risk occurred prior to the development of the condition or event that defined a participant as a case? 8. Were the measures of exposure/risk clearly defined, valid, reliable, and implemented consistently (including the same time period) across all study participants? 9. Defined 10. Valid/reliable 11. Implemented consistently 12. Were the assessors of exposure/risk blinded to the case or control status of participants? 13. Were key potential confounding variables measured and adjusted statistically in the analyses? If matching was used, did the investigators account for matching during study analysis? 14. Overall rating |
| --- |

|  |  |  |  |  | Control selection | | Participant selection | |  |  |  |  | Exposure measurement | | |  |  |  |
| --- | --- | --- | --- | --- | --- | --- | --- | --- | --- | --- | --- | --- | --- | --- | --- | --- | --- | --- |
| Study | Study design | Objective stated | Population defined | Sample size justification | Similar population | Similar timeframe | Consistent | Valid/ reliable | Adequate comparison group | Random selection | Concurrent control | Exposure preceded outcome | Defined | Valid/reliable | Implemented consistently | Assessor blinding | Confounders adjusted | Overall ROB |
| Agerbo 2003 | Case control | Yes | Yes | No^b^ | Yes | Yes | Yes | Yes | Yes | N/A | No | Yes | Yes | Yes | Yes | No | Yes | Good |
|  |  |  |  |  |  |  |  |  |  |  |  |  |  |  |  |  |  |  |
| Ahmadi 2015 | Case control | Yes | Yes | No | No | Yes | Yes | Yes | Yes | N/A | No | Yes | Yes | Yes | Yes | No | Yes | Fair |
| Chachamovich 2015 | Case control | Yes | Yes | No | No^c^ | Yes | Yes | Yes | Yes | N/A | No | No | Partial | Partial^d^ | Yes | Yes | Yes | Fair |
| Christiansen 2011 | Case control | Yes | Yes | No | Yes | Yes | Yes | Yes | Yes | N/A | No | Yes | Yes | Yes | Yes | No | Yes | Good |
| Gould 1996 | Case control | Yes | Yes | Yes | No^c^ | No | Yes | Yes | Yes | N/A | No | Yes | Yes | Partial^d^ | Yes | No | Yes | Fair |
| Gray 2014 | Case control | Yes | Yes | No | Yes | Yes | Yes | Yes | Yes | N/A | No | Yes | Yes | Partial^d^ | Yes | No | Yes | Fair |
| Hu 2017 | Case control | Yes | Yes | Yes | Yes | Yes | Yes | Yes | Yes | N/A | No | Yes | Yes | Yes | Yes | No | Yes | Good |
| Palacio 2007 | Case control | Yes | Yes | Yes | Yes | Yes | Yes | Yes | Yes | N/A | No | Yes | No | Partial^d^ | Yes | No | Yes | Poor |
| Tidemalm 2011 | Case control | Yes | Yes | Yes | Yes | Yes | Yes | Yes | Yes | N/A | No | Yes | Yes | Yes | Yes | No | Yes | Good |
| Vijayakumar 1999 | Case control | No^a^ | No | Yes | No | No | Yes | Yes | Yes | N/A | No | Yes | Yes | Partial^d^ | Yes | No | Yes | Fair |
| Conner 2007 | Case control | No^a^ | Yes | Yes | Yes | Yes | Yes | Yes | Yes | Yes | No | Yes | Yes | Yes | Yes | No | Yes | Fair |
| Foster 1999 | Case control | Yes | Yes | No | No^c^ | Yes | Yes | Yes | Yes | N/A | No | Yes | Yes | Partial^d^ | Yes | No | Yes | Fair |
| Garfinkel 1982 | Case control | No^a^ | Yes | No | Yes | Yes | NR | No | Yes | N/A | No | Yes | No | NR | Yes | No | No | Poor |
| Katibeh 2018 | Case control | No^a^ | No | No | NR | NR | NR | No | No | N/A | No | Yes | Yes | Yes | Yes | No | Yes | Poor |
| Mittendorfer-Rutz 2008 | Case control | Yes | Yes | No^b^ | Yes | Yes | Yes | Yes | Yes | N/A | No | Yes | Yes | Yes | Yes | No | Yes | Good |
| Giupponi 2018 | Case control | Yes | Yes | No | No^c^ | Yes | Yes | Yes | Yes | N/A | No | Yes | Yes | Partial^d^ | Yes | No | Yes | Fair |
| Liu 2019 | Case control | Yes | Yes | No | No^c^ | Yes | Yes | Yes | Yes | N/A | No | Yes | Yes | Partial^d^ | Yes | No | Yes | Fair |
| Cheng 2000 | Case control | Yes | Yes | Yes | No^c^ | Yes | Yes | Yes | Yes | N/A | No | Yes | No | No^d,e^ | Yes | No | Yes | Poor |
| Jollant 2014 | Case control | Yes | Yes | Yes | No | No | Yes | Yes | Yes | N/A | No | No | Yes | No^d,e^ | No | No | No | Poor |
| Mercy 2001 | Case control | Yes | Yes | No | Yes | Yes | Yes | Yes | Yes | N/A | No | Yes | Yes | No^e^ | Yes | No | Yes | Fair |
| Maniam 1994 | Case control | Yes | Yes | Yes | No^c^ | NR | Yes | Yes | Yes | N/A | No | Yes | No | No^d,e^ | Yes | No | No | Poor |
| Phillips 2002 | Case control | No^a^ | Yes | Yes | Yes | Yes | Yes | Yes | Yes | Yes | No | Yes | No | No^d,e^ | Yes | No | Yes | Poor |

NR-Not reported

^a^Exploratory study

^b^Population based study

^c^ Living control

^d^Psychological autopsy

^e^Exposure to suicidal behavior composite measure

## Cohort and cross-sectional studies

##

| 1. Was the research question or objective in this paper clearly stated? 2. Was the study population clearly specified and defined? 3. Was the participation rate of eligible persons at least 50%? 4. Were all the subjects selected or recruited from the: 5. Same or similar populations 6. Including the same time period? 7. Were inclusion and exclusion criteria for being in the study prespecified and applied uniformly to all participants? 8. Was a sample size justification, power description, or variance and effect estimates provided? 9. For the analyses in this paper, were the exposure(s) of interest measured prior to the outcome(s) being measured? 10. Was the timeframe sufficient so that one could reasonably expect to see an association between exposure and outcome if it existed? 11. For exposures that can vary in amount or level, did the study examine different levels of the exposure as related to the outcome (e.g., categories of exposure, or exposure measured as continuous variable (e.g. time since exposure, proximity, type of self-harm or suicide) 12. Were the exposure measures (independent variables) clearly: 13. Defined 14. Valid/reliable 15. Implemented consistently across all study participants?   10. Was the exposure(s) assessed more than once over time?  11. Were the outcome measures (dependent variables) clearly:   1. Defined 2. Valid/reliable 3. Implemented consistently across all study participants? 4. Were the outcome assessors blinded to the exposure status of participants? 5. Was loss to follow-up after baseline 20% or less? 6. Were key potential confounding variables measured and adjusted statistically for their impact on the relationship between exposure(s) and outcome(s)? 7. Overall rating |
| --- |

|  |  |  |  |  | Subject recruitment | | |  |  |  |  | Exposure measurement | | |  | Outcome measurement | | |  |  |  |  |
| --- | --- | --- | --- | --- | --- | --- | --- | --- | --- | --- | --- | --- | --- | --- | --- | --- | --- | --- | --- | --- | --- | --- |
| Study | Study design | Objective stated | Population defined | Participation ≥50% | Similar population | Similar timeframe | Consistent | Variance and effect estimates provided | Exposure preceded outcome | Sufficient timeframe | Measured levels of exposure | Defined | Valid/reliable | Implemented consistently | Assessed more than once | Defined | Valid/reliable | Implemented consistently | Assessor blinded | Follow-up ≤20% | Confounders adjusted | Overall ROB |
| Almeida 2012 | Cross-sec | Yes | Yes | Yes | Yes | yes | Yes | Yes | No | Yes | Yes | Yes | Yes | Yes | No | Yes | Yes | Yes | No | N/A | Yes | Good |
| Brent 1996 study a | Cohort | Yes | Yes | Yes | Yes | Yes | Yes | Yes | Yes | Yes | Yes | Yes | Yes | Yes | No | Yes | Yes | Yes | No | Yes | Yes | Good |
| Brent 1996 study b | Cohort | Yes | Yes | Yes | Yes | Yes | Yes | Yes | Yes | Yes | Yes | Yes | Yes | Yes | No | Yes | Yes | Yes | No | Yes | Yes | Good |
| Brent 2015 | Cross-sec | Yes | Yes | Yes | Yes | Yes | Yes | Yes | No | Yes | Yes | Yes | Yes | Yes | No | Yes | Yes | Yes | Yes | N/A | Yes | Good |
| Gravseth 2010 | Cohort | Yes | Yes | Yes | Yes | Yes | Yes | Yes | Yes | Yes | Yes | Yes | Yes | Yes | Yes | Yes | Yes | Yes | No | Yes | Yes | Good |
| Nrugham 2007 | Cross-sec | Yes | Yes | Yes | Yes | Yes | Yes | Yes | No | Yes | Yes | No | No | Yes | No | No | Partial | Yes | No | N/A | Yes | Poor |
| Swanson 2013 | Cohort | Yes | Yes | Yes | Yes | Yes | Yes | Yes | Yes | Yes | Yes | Yes | Yes | Yes | No | No | No | Yes | No | Yes | Yes | Fair |
| Thompson 2011 | Cohort | Yes | Yes | Yes | Yes | Yes | Yes | Yes | Yes | Yes | Yes | Partial | Partial | Yes | Yes | Yes | Yes | Yes | No | No | Yes | Fair |
| Lewinsohn 1994 | Cohort | Yes | Yes | Yes | Yes | Yes | Yes | Yes | Yes | Yes | Yes | Yes | Yes | Yes | Yes | Yes | Yes | Yes | No | Yes | Yes | Good |
| Chan 2018 | Cross-sec | Yes | Yes | Yes | Yes | Yes | Yes | Yes | Yes | Yes | Yes | Partial | Partial | Yes | No | Yes | Partial | Yes | No | Yes | Yes | Fair |
| Hishinuma 2018 | Cohort | Yes | Yes | Yes | Yes | Yes | Yes | Yes | Yes | Yes | Yes | Yes | Yes | Yes | Yes | Yes | Yes | Yes | No | No | Yes | Fair |
| Lee 2018 | Cohort | Yes | Yes | Yes | Yes | Yes | Yes | Yes | Yes | Yes | Yes | Yes | Yes | Yes | Yes | Yes | Yes | Yes | No | Yes | Yes | Good |
