# Supplementary material for: Association of suicidal behavior with exposure to suicide and suicide attempt: A systematic review and multilevel meta-analysis
Source: PLoS Med. 2020 Mar 31;17(3):e1003074. doi: 10.1371/journal.pmed.1003074 (PMC7108695; doi:10.1371/journal.pmed.1003074)
Supplement: S2 Text — (DOCX) [file pmed.1003074.s009.docx]

# S2 Text. Medline search strategy

| 1. | (Suicid* or Self-harm or self-injur* or self-mutilat*).mp. [mp=title, abstract, original title, name of substance word, subject heading word, floating sub-heading word, keyword heading word, protocol supplementary concept word, rare disease supplementary concept word, unique identifier, synonyms] |
| --- | --- |
| 2. | (bereav* or grief* or griev* or mourn* or widow* or expos* or contagio*).mp. [mp=title, abstract, original title, name of substance word, subject heading word, floating sub-heading word, keyword heading word, protocol supplementary concept word, rare disease supplementary concept word, unique identifier, synonyms] |
| 3. | (((Suicid* or Self-harm or self-injur* or self-mutilat*) and cluster*) not (ClusterADJanalys* or ClusterADJrandom*)).ab. |
| 4. | exp Genetic Predisposition to Disease/ |
| 5. | Family Characteristics/ |
| 6. | (Familial or family history or genetic predisposition).mp. [mp=title, abstract, original title, name of substance word, subject heading word, floating sub-heading word, keyword heading word, protocol supplementary concept word, rare disease supplementary concept word, unique identifier, synonyms] |
| 7. | (famil* or relative or friend or suriviv* or spous* or Parent or child* or undergraduate or student or school* or sibling).mp. [mp=title, abstract, original title, name of substance word, subject heading word, floating sub-heading word, keyword heading word, protocol supplementary concept word, rare disease supplementary concept word, unique identifier, synonyms] |
| 8. | 1 and 2 |
| 9. | 4 or 5 or 6 |
| 10. | 1 and 9 |
| 11. | 3 or 8 or 10 |
| 12. | 7 and 11 |
