# Supplementary material for: Association of suicidal behavior with exposure to suicide and suicide attempt: A systematic review and multilevel meta-analysis
Source: PLoS Med. 2020 Mar 31;17(3):e1003074. doi: 10.1371/journal.pmed.1003074 (PMC7108695; doi:10.1371/journal.pmed.1003074)
Supplement: S3 Text — (DOCX) [file pmed.1003074.s010.docx]

# S3 Text. Excluded articles

# Not exposed to suicidal behaviour (Suicide attempt/self-harm or suicide death)

1. Abou Abbas O, AlBuhairan F. Predictors of adolescents' mental health problems in Saudi Arabia: Findings from the Jeeluna national study. Child and Adolescent Psychiatry and Mental Health. 2017;11(1):52.

2. Adam KS, Lohrenz JG, Harper D. Suicidal ideation and parental loss. A preliminary research report. Canadian Psychiatric Association journal. 1973;18(2):95-100.

3. Adam KS, Lohrenz JG, Harper D, Streiner D. Early parental loss and suicidal ideation in university students. Canadian journal of psychiatry Revue canadienne de psychiatrie. 1982;27(4):275-81.

4. Adrian M, Miller AB, McCauley E, Vander Stoep A. Suicidal ideation in early to middle adolescence: sex-specific trajectories and predictors. Journal of Child Psychology and Psychiatry. 2016;57(5):645-53.

5. Ahuja M, Werner KB, Bucholz KK. Examining substance use and suicidal thoughts and behavior among African American and European American adolescents and young adults. Alcoholism: Clinical and Experimental Research. 2019;43 (Supplement 1):239A.

6. Alfonso ML, Kaur R. Self-Injury Among Early Adolescents: Identifying Segments Protected and at Risk. Journal of School Health. 2012;82(12):537-47.

7. An H, Ahn JH, Bhang SY. The association of psychosocial and familial factors with adolescent suicidal ideation: A population-based study. Psychiatry Research. 2010;177(3):318-22.

8. Andrea MM, Castellví P, Alayo I, Vilagut G, Maria Jesús B, Torrent A, et al. Gender commonalities and differences in risk and protective factors of suicidal thoughts and behaviors: A cross‐sectional study of Spanish university students. Depression and Anxiety. 2019;36(11):1102-14.

9. Anes H, Csilla C. Suicidal ideation and suicidal attempt in a representative sample of young females. Psychiatria Hungarica. 2001;16(2):134-44.

10. Ang AL, Wahab S, Abd Rahman FN, Hazmi H, Rosliwati Md Y. Depressive symptoms in adolescents in Kuching, Malaysia: Prevalence and associated factors. Pediatrics International. 2019;61(4):404-10.

11. Appel CW, Johansen C, Christensen J, Frederiksen K, Hjalgrim H, Dalton SO, et al. Risk of use of antidepressants among children and young adults exposed to the death of a parent. Epidemiology. 2016;27(4):578-85.

12. Ariapooran S, Heidari S, Asgari M, Ashtarian H, Khezeli M. Individualism-Collectivism, Social Support, Resilience and Suicidal Ideation among Women with the Experience of the Death of a Young Person. International Journal of Community Based Nursing & Midwifery. 2018;6(3):250-9.

13. Asgeirsdottir HG, Valdimarsdottir UA, orsteinsdottir K, Lund SH, Tomasson G, Nyberg U, et al. The association between different traumatic life events and suicidality. European Journal of Psychotraumatology. 2018;9(1):1510279.

14. Auerbach RP, Mortier P, Bruffaerts R, Alonso J, Benjet C, Cuijpers P, et al. WHO World Mental Health Surveys International College Student Project: Prevalence and distribution of mental disorders. Journal of Abnormal Psychology. 2018;127(7):623-38.

15. Bae SM, Lee YJ, Cho IH, Kim SJ, Im JS, Cho SJ. Risk factors for suicidal ideation of the general population. Journal of Korean Medical Science. 2013;28(4):602-7.

16. Bailly D, Alexandre JY, Collinet C, Beuscart R, Parquet Ph J. Depression in adolescents: A study in a high school student population. Psychiatrie et Psychobiologie. 1990;5(6):363-73.

17. Baldwin JR, Arseneault L, Caspi A, Moffitt TE, Fisher HL, Odgers CL, et al. Adolescent Victimization and Self-Injurious Thoughts and Behaviors: A Genetically Sensitive Cohort Study. Journal of the American Academy of Child & Adolescent Psychiatry. 2019;58(5):506-13.

18. Balk D. Adolescents' grief reactions and self-concept perceptions following sibling death: A study of 33 teenagers. Journal of youth and adolescence. 1983;12(2):137-61.

19. Ballard ED, Van Eck K, Musci RJ, Hart SR, Storr CL, Breslau N, et al. Latent classes of childhood trauma exposure predict the development of behavioral health outcomes in adolescence and young adulthood. Psychological medicine. 2015;45(15):3305-16.

20. Banerjee S, Chowdhury AN, Schelling E, Weiss MG. Household survey of pesticide practice, deliberate self-harm, and suicide in the sundarban region of West Bengal, India. BioMed Research International. 2013;2013:949076.

21. Baroud E, Ghandour LA, Alrojolah L, Zeinoun P, Maalouf FT. Suicidality among Lebanese adolescents: Prevalence, predictors and service utilization. Psychiatry Research. 2019;275:338-44.

22. Barzilay R, Calkins ME, Moore TM, Wolf DH, Satterthwaite TD, Cobb Scott J, et al. Association between traumatic stress load, psychopathology, and cognition in the Philadelphia Neurodevelopmental Cohort. Psychological medicine. 2018:1-10.

23. Beckman K, Lysell H, Haglund A, Dahlin M. Prognoses after self-harm in youth: exploring the gender factor. Social Psychiatry and Psychiatric Epidemiology. 2019;54(4):437-44.

24. Belik SL, Stein MB, Asmundson GJG, Sareen J. Relation between traumatic events and suicide attempts in canadian military personnel. Canadian Journal of Psychiatry. 2009;54(2):93-104.

25. Bellis MA, Hughes K, Leckenby N, Jones L, Baban A, Kachaeva M, et al. Adverse childhood experiences and associations with health-harming behaviours in young adults: surveys in eight eastern European countries. Bulletin of the World Health Organization. 2014;92(9):641-55.

26. Beristianos MH, Maguen S, Neylan TC, Byers AL. Trauma Exposure and Risk of Suicidal Ideation Among Older Adults. American Journal of Geriatric Psychiatry. 2016;24(8):639-43.

27. Bhattacharyya D, Timothy A, Yadav P, Namdev M. Survivors of deliberate self-harm attempt in the military milieu: An exploratory study of psychiatric morbidity and psychosocial correlates. Medical Journal Armed Forces India. 2019;75(2):197-203.

28. Bioulac S, Bourgeois M, Ekouevi DK, Bonnin JM, Gonzales B, Castello MF. Predictive factors of suicide: An 8-year prospective longitudinal study of 200 psychiatric in patients. Encephale. 2000;26(1):1-7.

29. Birtchnell J. Women whose mothers died in childhood: an outcome study. Psychological medicine. 1980;10(4):699-713.

30. Bjureberg J, Ohlis A, Ljotsson B, D'Onofrio BM, Hedman-Lagerlof E, Jokinen J, et al. Adolescent self-harm with and without suicidality: cross-sectional and longitudinal analyses of a Swedish regional register. Journal of Child Psychology & Psychiatry & Allied Disciplines. 2019;60(3):295-304.

31. Black DW, Okiishi C, Gabel J, Schlosser S. Psychiatric illness in the first-degree relatives of persons reporting multiple chemical sensitivities. Toxicology and Industrial Health. 1999;15(3-4):410-4.

32. Blum R, Sudhinaraset M, Emerson MR. Youth at Risk: Suicidal Thoughts and Attempts in Vietnam, China, and Taiwan. Journal of Adolescent Health. 2012;50(3s):S37-S44.

33. Boardman AP, Grimbaldeston AH, Handley C, Jones PW, Willmott S. The North Staffordshire Suicide Study: A case-control study of suicide in one health district. Psychological Medicine. 1999;29(1):27-33.

34. Bombay A, McQuaid RJ, Schwartz F, Thomas A, Anisman H, Matheson K. Suicidal thoughts and attempts in First Nations communities: links to parental Indian residential school attendance across development. Journal of Developmental Origins of Health and Disease. 2019;10(1):123-31.

35. Boothroyd LJ, Kirmayer LJ, Spreng S, Malus M, Hodgins S. Completed suicides among the Inuit of northern Quebec, 1982-1996: A case-control study. CMAJ. 2001;165(6):749-55.

36. Boyda D, Feeters DM, Dhingra K, Galbraith N, Hinton D. Parental psychopathology, adult attachment and risk of 12-month suicidal behaviours. Psychiatry Research. 2018;260:272-8.

37. Bracic M, Roskar S, Zager Kocjan G, Jericek Klanscek H. The Prevalence and Predictors of Suicidal Ideation Among Slovene Adolescents. Community Mental Health Journal. 2019:29.

38. Bromet EJ, Haveenaar JM, Tintle N, Kostyuchenko S, Kotov R, Gluzman S. Suicide ideation, plans and attempts in Ukraine: Findings from the Ukraine World Mental Health Survey. Psychological Medicine. 2007;37(6):807-19.

39. Bronisch T, Hecht H. Comparison of depressed patients with and without suicide attempts in their past history. Acta psychiatrica Scandinavica. 1987;76(4):438-49.

40. Bruffaerts R, Demyttenaere K, Borges G, Haro JM, Chiu WT, Hwang I, et al. Childhood adversities as risk factors for onset and persistence of suicidal behaviour. The British journal of psychiatry : the journal of mental science. 2010;197(1):20-7.

41. Bunch J, Barraclough B, Nelson B, Sainsbury P. Suicide following bereavement of parents. Social Psychiatry. 1971;6(4):193-9.

42. Bunch J, Barraclough E, Nelson B, Sainsbury P. Early parental bereavement and suicide. Social Psychiatry. 1971;6(4):200-2.

43. Byrne GJA, Raphael B. The psychological symptoms of conjugal bereavement in elderly men over the first 13 months. International Journal of Geriatric Psychiatry. 1997;12(2):241-51.

44. Calik B, Acikgoz A. Identifying the affecting factors in adolescents who attempt suicide. Annals of Clinical and Analytical Medicine. 2020;11(1):43-9.

45. Cassorla RM. Young people who attempt suicide, morbid and auto-destructive antecedents: A comparison study with normal young people and with young people with mental problems: II. Jovens que Tentam Suicidio Antecendentes Morbidos e de Condutas Autodestrutivas-Um estudo comparativo com jovens normais e com problemas mentais (II). 1984;33(2):93-8.

46. Cervilla JA, Gutierrez B, Rodriguez-Barranco M, Ibanez-Casas I, Perez-Garcia M, Valmisa E, et al. A Cross-Sectional Study on the Prevalence and Risk Correlates of Mental Disorders: The GRANADSIGMAP Study. Journal of Nervous & Mental Disease. 2018;206(9):716-25.

47. Chen JH, Bierhals AJ, Prigerson HG, Kasl SV, Mazure CM, Jacobs S. Gender differences in the effects of bereavement-related psychological distress in health outcomes. Psychological Medicine. 1999;29(2):367-80.

48. Choi B. Job strain, long work hours, and suicidal ideation in US workers: a longitudinal study. International archives of occupational and environmental health. 2018.

49. Christiansen E, Larsen KJ, Agerbo E, Bilenberg N, Stenager E. Incidence and risk factors for suicide attempts in a general population of young people: A Danish register-based study. Australian and New Zealand Journal of Psychiatry. 2013;47(3):259-70.

50. Clayton PJ, Halikes JA, Maurice WL. The bereavement of the widowed. Diseases of the Nervous System. 1971;32(9):597-604.

51. Coleman C, Wileyto EP, Lenhart CM, Patterson F. Multiple Health Risk Behaviors in Adolescents: An Examination of Youth Risk Behavior Survey Data. American Journal of Health Education. 2014;45(5):271-7.

52. Collaborators GBDEMRAH. Adolescent health in the Eastern Mediterranean Region: findings from the global burden of disease 2015 study. International Journal of Public Health. 2018;63(Suppl 1):79-96.

53. Cong EZ, Wu Y, Cai YY, Chen HY, Xu YF. [Association of suicidal ideation with family environment and psychological resilience in adolescents]. Zhongguo Dangdai Erke Zazhi. 2019;21(5):479-84.

54. Connell HM. Attempted suicide in schoolchildren. The Medical journal of Australia. 1972;1(14):686-90.

55. Corcoran P. The impact of widowhood on Irish mortality due to suicide and accidents. European journal of public health. 2009;19(6):583-5.

56. Cramer RJ, Bryson CN, Gardner BO, Webber WB. Can preferences in information processing aid in understanding suicide risk among emerging adults? Death Studies. 2016;40(6):383-91.

57. Cromer KD, Villodas MT, Chou T. Gender differences in psychological distress as a mediational pathway to suicidal ideation among adolescents at high risk for victimization by violence. Psychology of Violence. 2019;9(6):587-95.

58. Dachew BA, Bifftu BB, Tiruneh BT, Anlay DZ, Wassie MA. Suicidal thoughts among university students in Ethiopia. Annals of General Psychiatry. 2018;17(1):1.

59. De Jong ML. Attachment, Individuation, and Risk of Suicide in Late Adolescence. Journal of Youth and Adolescence. 1992;21(3):357-73.

60. De Luca SM. Latina adolescent suicide: Examining the effects of cultural status and parental, peer and teacher supports.

61. de Man AF, Labreche-Gauthier L, Leduc CP. Parent-child relationships and suicidal ideation in French-Canadian adolescents. The Journal of genetic psychology ; child behavior, animal behavior, and comparative psychology. 1993;154(1):17-23.

62. De Vanna M, Paterniti S, Milievich C, Rigamonti R, Sulich A, Faravelli C. Recent life events and attempted suicide. Journal of affective disorders. 1990;18(1):51-8.

63. Delker BC, Freyd JJ. From betrayal to the bottle: investigating possible pathways from trauma to problematic substance use. Journal of traumatic stress. 2014;27(5):576-84.

64. Dhingra K, Boduszek D, Klonsky ED. Empirically Derived Subgroups of Self-Injurious Thoughts and Behavior: Application of Latent Class Analysis. Suicide & life-threatening behavior. 2016;46(4):486-99.

65. Dhingra K, Boduszek D, O'Connor RC. Differentiating suicide attempters from suicide ideators using the Integrated Motivational-Volitional model of suicidal behaviour. Journal of Affective Disorders. 2015;186:211-8.

66. Diaz AP, Svob C, Zhao R, DiFabrizio B, Warner V, Gameroff MJ, et al. Adult outcomes of childhood disruptive disorders in offspring of depressed and healthy parents. Journal of Affective Disorders. 2019;244:107-12.

67. Dil S, Gonen Senturk S, Aykanat Girgin B. Relationship between risky health behaviors and some demographic characteristics of adolescents' self-esteem and healthy lifestyle behaviors in Cankiri. Anadolu Psikiyatri Dergisi. 2015;16(1):51-9.

68. Dizmang LH, Watson J, May PA, Bopp J. Adolescent suicide at an Indian reservation. American Journal of Orthopsychiatry. 1974;44(1):43-9.

69. Doi S, Fujiwara T. Combined effect of adverse childhood experiences and young age on self-harm ideation among postpartum women in Japan. Journal of Affective Disorders. 2019;253:410-8.

70. Donath C, Bergmann MC, Kliem S, Hillemacher T, Baier D. Epidemiology of suicidal ideation, suicide attempts, and direct self-injurious behavior in adolescents with a migration background: a representative study. BMC Pediatrics. 2019;19(1):45.

71. Dowdney L, Wilson R, Maughan B, Allerton M, et al. Psychological disturbance and service provision in parentally bereaved children: Prospective case-control study. British Medical Journal. 1999;319(7206):354-7.

72. Du Roscoat E, Legleye S, Guignard R, Husky M, Beck F. Risk factors for suicide attempts and hospitalizations in a sample of 39,542 French adolescents. Journal of Affective Disorders. 2016;190:517-21.

73. Dube SR, Anda RF, Felitti VJ, Chapman DP, Williamson DF, Giles WH. Childhood abuse, household dysfunction, and the risk of attempted suicide throughout the life span: findings from the Adverse Childhood Experiences Study. JAMA. 2001;286(24):3089-96.

74. Dupere V, Leventhal T, Lacourse E. Neighborhood poverty and suicidal thoughts and attempts in late adolescence. Psychological Medicine. 2009;39(8):1295-306.

75. Dutta R, Ball HA, Siribaddana SH, Sumathipala A, Samaraweera S, McGuffin P, et al. Genetic and other risk factors for suicidal ideation and the relationship with depression. Psychological Medicine. 2017;47(14):2438-49.

76. Eisinger GJ, Wodarski JS, Ferguson D. Psychosocial Correlates of Methamphetamine Use. Journal of Human Behavior in the Social Environment. 2009;19(7):915-31.

77. Elklit A. Victimization and PTSD in a Danish National Youth Probability Sample. Journal of the American Academy of Child and Adolescent Psychiatry. 2002;41(2):174-81.

78. Fan YG, Xiao Q, Wang Q, Li WX, Dong MX, Ye DQ. [Study on the influencing factors related to suicide ideation among undergraduates in Anhui province]. Zhonghua liu xing bing xue za zhi = Zhonghua liuxingbingxue zazhi. 2008;29(3):241-4.

79. Faris N, Baroud E, Al Hariri M, Bachir R, El-Khoury J, Batley NJ. Characteristics and dispositional determinants of psychiatric emergencies in a University Hospital in Beirut. Asian Journal of Psychiatry. 2019;42:42-7.

80. Felitti VJ, Anda RF, Nordenberg D, Williamson DF, Spitz AM, Edwards V, et al. Relationship of childhood abuse and household dysfunction to many of the leading causes of death in adults: The adverse childhood experiences (ACE) study. American Journal of Preventive Medicine. 1998;14(4):245-58.

81. Fergusson DM, Horwood LJ, Lynskey M. The childhoods of multiple problem adolescents: A 15-year longitudinal study. Journal of Child Psychology and Psychiatry and Allied Disciplines. 1994;35(6):1123-40.

82. Fergusson DM, Lynskey MT. Suicide attempts and suicidal ideation in a birth cohort of 16-year-old New Zealanders. Journal of the American Academy of Child and Adolescent Psychiatry. 1995;34(10):1308-17.

83. Fergusson DM, Woodward LJ, Horwood LJ. Risk factors and life processes associated with the onset of suicidal behaviour during adolescence and early adulthood. Psychological Medicine. 2000;30(1):23-39.

84. Fernandez Rivas A, Gonzalez Torres MA, Lasa Zulueta A. Differential characteristics of families of adolescents and young adults that have attempted suicide. Actas luso-espanolas de neurologia, psiquiatria y ciencias afines. 1998;26(2):97-103.

85. Fjeldsted R, Teasdale TW, Jensen M, Erlangsen A. Suicide in Relation to the Experience of Stressful Life Events: A Population-Based Study. Archives of Suicide Research. 2017;21(4):544-55.

86. Flannery DJ, Singer MI, Wester K. Violence exposure, psychological trauma, and suicide risk in a community sample of dangerously violent adolescents. Journal of the American Academy of Child and Adolescent Psychiatry. 2001;40(4):435-42.

87. Flisher AJ, Kramer RA, Hoven CW, King RA, Bird HR, Davies M, et al. Risk behavior in a community sample of children and adolescents. Journal of the American Academy of Child and Adolescent Psychiatry. 2000;39(7):881-7.

88. Gao T, Yu-Tao X, Zhang H, Gao J, Kong Y, Hu Y, et al. Prevalence and Correlates of Suicidal Behaviors among College Students in Northeastern China: a Cross-Sectional Study. Psychiatric Quarterly. 2018;89(2):359-70.

89. Griffith J. Suicide in the U.S. Army: Stressor-strain hypothesis among deployed and nondeployed army national guard soldiers. Journal of Aggression, Conflict and Peace Research. 2015;7(3):187-98.

90. Groholt B, Oivind E, Wichstrom L, Haldorsen T. Suicide among children and younger and older adolescents in Norway: A comparative study. Journal of the American Academy of Child and Adolescent Psychiatry. 1998;37(5):473-81.

91. Guarmit B, Brousse P, Lucarelli A, Donutil G, Cropet C, Mosnier E, et al. Descriptive epidemiology of suicide attempts and suicide in the remote villages of French Guiana. Social Psychiatry & Psychiatric Epidemiology. 2018;53(11):1197-206.

92. Hamdullahpur K, Jacobs KWJ, Gill KJ. Mental Health Among Help-Seeking Urban Women: The Relationships Between Adverse Childhood Experiences, Sexual Abuse, and Suicidality. Violence against women. 2018:1077801218761602.

93. Harris LH. Role of trauma in the lives of high school dropouts. Social Work in Education. 1983;5(2):77-88.

94. Herzog AN, Levy L, Verdonk A. Some ecological factors associated with health and social adaptation in the city of Rotterdam. Urban Ecology. 1977;2(3):205-34.

95. Hill OW. The association of childhood bereavement with suicidal attempt in depressive illness. The British journal of psychiatry : the journal of mental science. 1969;115(520):301-4.

96. Hill OW, Price JS. Childhood bereavement and adult depression. The British journal of psychiatry : the journal of mental science. 1967;113(500):743-51.

97. Hill RM, Kaplow JB, Oosterhoff B, Layne CM. Understanding grief reactions, thwarted belongingness, and suicide ideation in bereaved adolescents: Toward a unifying theory. Journal of Clinical Psychology. 2019;75(4):780-93.

98. Hollingshaus MS, Smith KR. Life and death in the family: Early parental death, parental remarriage, and offspring suicide risk in adulthood. Social Science & Medicine. 2015;131:181.

99. Hollis C. Depression, family environment, and adolescent suicidal behavior. Journal of the American Academy of Child and Adolescent Psychiatry. 1996;35(5):622-30.

100. Huang HW, Wang RH. Roles of protective factors and risk factors in suicidal ideation among adolescents in Taiwan. Public Health Nursing. 2019;36(2):155-63.

101. Hughes K, Bellis MA, Sethi D, Andrew R, Yon Y, Wood S, et al. Adverse childhood experiences, childhood relationships and associated substance use and mental health in young Europeans. European Journal of Public Health. 2019:20.

102. Jao NCMS, Robinson LDP, Kelly PJP, Ciecierski CCP, Hitsman BP. Unhealthy behavior clustering and mental health status in United States college students. Journal of American College Health. 2019;67(8):790-800.

103. Jessen G, Andersen K, Bille-Brahe U. Suicidal thoughts and suicidal attempts among 15-24 years old individuals in the Danish educational system. Ugeskrift for laeger. 1996;158(36):5026-9.

104. Joiner Jr TE. Contagion of suicidal symptoms as a function of assortative relating and shared relationship stress in college roommates. Journal of Adolescence. 2003;26(4):495-504.

105. Jones MB, Jones DR. Preferred pathways of behavioral contagion. Journal of Psychiatric Research. 1995;29(3):193-209.

106. Karami M, Yazdi-Ravandi S, Ghaleiha A, Olfatifar M. Comparison of the Clusters and Non-Clusters Areas of Attempted Suicide Cases in Hamadan Province, Western Iran: Findings from a Pilot Study (2016-2017). Journal of Research in Health Sciences. 2018;18(3):e00425.

107. Karsberg S, Armour C, Elklit A. Patterns of victimization, suicide attempt, and posttraumatic stress disorder in Greenlandic adolescents: a latent class analysis. Social psychiatry and psychiatric epidemiology. 2014;49(9):1389-99.

108. Kazanasmaz H, Kazanasmaz O, Calik M. Epidemiological and sociocultural assessment of childhood poisonings. Turkish Journal of Emergency Medicine. 2019;19(4):127-31.

109. Keeshin BR, Gray D, Zhang C, Presson AP, Coon H. Youth Suicide Deaths: Investigation of Clinical Predictors in a Statewide Sample. Suicide & Life-Threatening Behavior. 2018;48(5):601-12.

110. Kim DS. Experience of parent-related negative life events, mental health, and delinquent behavior among Korean adolescents. Journal of preventive medicine and public health = Yebang Uihakhoe chi. 2007;40(3):218-26.

111. Kim Mi Y, Yu J. Factors Contributing to Non-suicidal Self Injury in Korean Adolescents. Journal of Korean Academy of Community Health Nursing / Jiyeog Sahoe Ganho Hakoeji. 2017;28(3):271-9.

112. King RA, Schwab-Stone M, Flisher AJ, Greenwald S, Kramer RA, Goodman SH, et al. Psychosocial and risk behavior correlates of youth suicide attempts and suicidal ideation. Journal of the American Academy of Child and Adolescent Psychiatry. 2001;40(7):837-46.

113. Kinyanda E, Hoskins S, Nakku J, Nawaz S, Patel V. Prevalence and risk factors of major depressive disorder in HIV/AIDS as seen in semi-urban Entebbe district, Uganda. BMC Psychiatry. 2011;11:205.

114. Koller KM, Williams WT. Early parental deprivation and later behavioural outcomes: cluster analysis study of normal and abnormal groups. Australian and New Zealand Journal of Psychiatry. 1974;8(2):89-96.

115. Krupinski J, Tiller JWG, Burrows GD, Mackenzie A. Social and Familial Aspects of Attempted and Completed Suicide of Young People in Victoria. Australian Journal of Social Issues. 1998;33(4):323-33.

116. Kwak M, Ahn S. Childhood adversity predicted suicidal ideation in older age: Results from a National Survey in Korea. Aging & Mental Health. 2019:1-8.

117. L Beautrais A. Suicides and serious suicide attempts: two populations or one? Psychological Medicine. 2001;31(5):837-45.

118. Lansing AE, Plante WY, Beck AN. Assessing stress-related treatment needs among girls at risk for poor functional outcomes: The impact of cumulative adversity, criterion traumas, and non-criterion events. Journal of Anxiety Disorders. 2017;48:36-44.

119. Lavoie S, Talbot LR, Mathieu L, Dallaire C, Dubois M-F, Courcy F. An exploration of factors associated with post-traumatic stress in ER nurses. Journal of Nursing Management. 2016;24(2):174-83.

120. Law BMF, Shek DTL. A 6-year Longitudinal Study of Self-harm and Suicidal Behaviors among Chinese Adolescents in Hong Kong. Journal of Pediatric and Adolescent Gynecology. 2016;29(1):S38-S48.

121. Layne CM, Greeson JKP, Ostrowski SA, Kim S, Reading S, Vivrette RL, et al. Cumulative trauma exposure and high risk behavior in adolescence: Findings from the National Child Traumatic Stress Network Core Data Set. Special Issue: Recent Developments in Trauma Studies. 2014;6(Suppl 1):S40-S9.

122. LeBouthillier DM, McMillan KA, Thibodeau MA, Asmundson GJ. Types and Number of Traumas Associated With Suicidal Ideation and Suicide Attempts in PTSD: Findings From a U.S. Nationally Representative Sample. Journal of traumatic stress. 2015;28(3):183-90.

123. Lee CM, Mangurian C, Tieu L, Ponath C, Guzman D, Kushel M. Childhood Adversities Associated with Poor Adult Mental Health Outcomes in Older Homeless Adults: Results From the HOPE HOME Study. American Journal of Geriatric Psychiatry. 2017;25(2):107-17.

124. Lee D, Jung S, Park S, Lee K, Kweon Y-S, Lee E-J, et al. Youth suicide in Korea across the educational stages: A postmortem comparison of psychosocial characteristics of elementary, middle, and high school students. Crisis: The Journal of Crisis Intervention and Suicide Prevention. 2019:No Pagination Specified.

125. Lee G, Ham OK. Behavioral and psychosocial factors associated with suicidal ideation among adolescents. Nursing & Health Sciences. 2018;20(3):394-401.

126. Leiva Henriquez H, Alamos Lara L, Prussing Santibanez L, Uriarte Ruiz A. Suicide attempts: Clinical and epidemiological characteristics. Sixth Region of Chile 2002-2004. Anales de Pediatria. 2008;69(2):110-4.

127. Lester D. Experience of loss and subsequent suicide. Perceptual and motor skills. 1994;79(2):730.

128. Leveillee S, Doyon L, Cantinotti M. Evolution of paternal filicide-suicide in the province of Quebec. L'Encephale: Revue de psychiatrie clinique biologique et therapeutique. 2019;45(1):34-9.

129. Levey DF, Polimanti R, Cheng Z, Zhou H, Nunez YZ, Jain S, et al. Genetic associations with suicide attempt severity and genetic overlap with major depression. Transl Psychiatry. 2019;9(1):22.

130. Liang H, Flisher AJ, Chalton DO. Violence and substance use in adolescents with increasing severity of suicidal behavior. Archives of Suicide Research. 2003;7(1):29-40.

131. Liu BP, Wang XT, Liu ZZ, Wang ZY, Liu X, Jia CX. Stressful life events, insomnia and suicidality in a large sample of Chinese adolescents. Journal of Affective Disorders. 2019;249:404-9.

132. Liu BP, Zhang J, Chu J, Qiu HM, Jia CX, Hennessy DA. Negative life events as triggers on suicide attempt in rural China: a case-crossover study. Psychiatry Research. 2019;276:100-6.

133. Liu CH, Stevens C, Wong SHM, Yasui M, Chen JA. The prevalence and predictors of mental health diagnoses and suicide among U.S. college students: Implications for addressing disparities in service use. Depression and Anxiety. 2019;36(1):8-17.

134. Liu X, Sun Z, Yang Y. Parent-reported suicidal behavior and correlates among adolescents in China. Journal of Affective Disorders. 2008;105(1-3):73-80.

135. Luby JL, Whalen D, Tillman R, Barch DM. Clinical and Psychosocial Characteristics of Young Children With Suicidal Ideation, Behaviors, and Nonsuicidal Self-Injurious Behaviors. Journal of the American Academy of Child and Adolescent Psychiatry. 2019;58(1):117-27.

136. Macedo A, Sherr L, Tomlinson M, Skeen S, Roberts K. Parental Bereavement in Young Children Living in South Africa and Malawi: Understanding Mental Health Resilience. Journal of acquired immune deficiency syndromes (1999). 2018;78(4):390-8.

137. Macrynikola N, Miranda R, Soffer A. Social connectedness, stressful life events, and self-injurious thoughts and behaviors among young adults. Comprehensive Psychiatry. 2018;80:140-9.

138. McKeown RE, Garrison CZ, Cuffe SP, Waller JL, et al. Incidence and predictors of suicidal behaviors in a longitudinal sample of young adolescents. Journal of the American Academy of Child and Adolescent Psychiatry. 1998;37(6):612-9.

139. McLafferty M, O'Neill S, Murphy S, Armour C, Ferry F, Bunting B. The moderating impact of childhood adversity profiles and conflict on psychological health and suicidal behaviour in the Northern Ireland population. Psychiatry Research. 2018;262:213-20.

140. McLafferty M, O’Neill S, Murphy S, Armour C, Bunting B. Population attributable fractions of psychopathology and suicidal behaviour associated with childhood adversities in Northern Ireland. Child Abuse & Neglect. 2018;77:35.

141. Merrick MT, Ports KA, Ford DC, Afifi TO, Gershoff ET, Grogan-Kaylor A. Unpacking the impact of adverse childhood experiences on adult mental health. Child Abuse & Neglect. 2017;69:10.

142. Morales-Vives F, Duenas JM. Predicting Suicidal Ideation in Adolescent Boys and Girls: The Role of Psychological Maturity, Personality Traits, Depression and Life Satisfaction. Spanish Journal of Psychology. 2018;21:E10.

143. Moses T. Suicide Attempts Among Adolescents with Self-Reported Disabilities. Child Psychiatry and Human Development. 2018;49(3):420-33.

144. Naicker N, De Jager P, Naidoo S, Mathee A. Household Factors Associated with Self-Harm in Johannesburg, South African Urban-Poor Households. PLoS ONE. 2016;11(1):e0146239.

145. Netto LR, Cavalcanti-Ribeiro P, Pereira JL, Nogueira JF, Santos LL, Lira SB, et al. Clinical and socio-demographic characteristics of college students exposed to traumatic experiences: A census of seven college institutions in Northeastern Brazil. PLoS ONE. 2013;8(11):e78677.

146. Noh DPRN. Relational-Level Factors Influencing Suicidal Behaviors Among Korean Adolescents. Journal of Nursing Scholarship. 2019;51(6):634-41.

147. Oppenheimer CW, Stone LB, Hankin BL. The influence of family factors on time to suicidal ideation onsets during the adolescent developmental period. Journal of Psychiatric Research. 2018;104:72-7.

148. Paffenbarger Jr RS, Asnes DP. Chronic disease in former college students. iii. precursors of suicide in early and middle life. American Journal of Public Health. 1966;56(7):1026-36.

149. Pandey AR, Bista B, Dhungana RR, Aryal KK, Chalise B, Dhimal M. Factors associated with suicidal ideation and suicidal attempts among adolescent students in Nepal: Findings from Global School-based Students Health Survey. PLoS ONE [Electronic Resource]. 2019;14(4):e0210383.

150. Park S, Hong JP, Jeon HJ, Seong S, Cho MJ. Childhood exposure to psychological trauma and the risk of suicide attempts: The modulating effect of psychiatric disorders. Psychiatry Investigation. 2015;12(2):171-6.

151. Patalay P, Gage SH. Changes in millennial adolescent mental health and health-related behaviours over 10 years: a population cohort comparison study. International Journal of Epidemiology. 2019:27.

152. Paul E, Ortin A. Psychopathological mechanisms of early neglect and abuse on suicidal ideation and self-harm in middle childhood. European Child & Adolescent Psychiatry. 2019;28(10):1311-9.

153. Pearson RM, Culpin I, Loret de Mola C, Quevedo L, Murray J, Matijasevich A, et al. Transition to parenthood and mental health at 30 years: a prospective comparison of mothers and fathers in a large Brazilian birth cohort. Archives of Women's Mental Health. 2018:06.

154. Pereira AS, Willhelm AR, Koller SH, Almeida RMM. Risk and protective factors for suicide attempt in emerging adulthood. Ciencia & Saude Coletiva. 2018;23(11):3767-77.

155. Pina-Watson B, Castillo LG, Rodriguez KM, Ray S. Familial factors related to suicidal ideation of Latina adolescents in the United States. Archives of suicide research : official journal of the International Academy for Suicide Research. 2014;18(2):213-20.

156. Plener PL, Singer H, Goldbeck L. Traumatic events and suicidally in a German adolescent community sample. Journal of Traumatic Stress. 2011;24(1):121-4.

157. Pournaghash-Tehrani SS, Zamanian H, Amini-Tehrani M. The Impact of Relational Adverse Childhood Experiences on Suicide Outcomes During Early and Young Adulthood. Journal of Interpersonal Violence. 2019:886260519852160.

158. Radatz DL, Wright EM. Does Polyvictimization Affect Incarcerated and Non-Incarcerated Adult Women Differently? An Exploration Into Internalizing Problems. Journal of Interpersonal Violence. 2017;32(9):1379-400.

159. Rhiger M, Elklit A, Lasgaard M. Traumatic in Israeli Youth Sample: An Investigation of the Prevalence and Psychological Impact of Exposure to Traumatic Experiences. Nordic Psychology. 2008;60(2):101-13.

160. Richmond-Rakerd LS, Caspi A, Arseneault L, Baldwin JR, Danese A, Houts RM, et al. Adolescents Who Self-Harm and Commit Violent Crime: Testing Early-Life Predictors of Dual Harm in a Longitudinal Cohort Study. American Journal of Psychiatry. 2019;176(3):186-95.

161. Richmond-Rakerd LS, Trull TJ, Gizer IR, McLaughlin K, Scheiderer EM, Nelson EC, et al. Common genetic contributions to high-risk trauma exposure and self-injurious thoughts and behaviors. Psychological Medicine. 2019;49(3):421-30.

162. Rihmer Z, Gonda X, Eory A, Kalabay L, Torzsa P. [Screening of depression in primary care in Hungary and its importance in suicide prevention]. Psychiatria Hungarica : A Magyar Pszichiatriai Tarsasag tudomanyos folyoirata. 2012;27(4):224-32.

163. Roy A. Characteristics of HIV patients who attempt suicide. Acta Psychiatrica Scandinavica. 2003;107(1):41-4.

164. Ruderfer DM, Walsh CG, Aguirre MW, Tanigawa Y, Ribeiro JD, Franklin JC, et al. Significant shared heritability underlies suicide attempt and clinically predicted probability of attempting suicide. Molecular Psychiatry. 2019:04.

165. Rusu C, Zamorski MA, Boulos D, Garber BG. Prevalence Comparison of Past-year Mental Disorders and Suicidal Behaviours in the Canadian Armed Forces and the Canadian General Population. Canadian journal of psychiatry Revue canadienne de psychiatrie. 2016;61(1 Supplement):46S-55S.

166. Rytila-Manninen M, Haravuori H, Frojd S, Marttunen M, Lindberg N. Mediators between adverse childhood experiences and suicidality. Child Abuse & Neglect. 2018;77:99-109.

167. Santana GL, Coelho BM, Borges G, Viana MC, Wang YP, Andrade LH. The influence of parental psychopathology on offspring suicidal behavior across the lifespan. PLoS ONE. 2015;10(7):e0134970.

168. Schnell T, Gerstner R, Krampe H. Crisis of Meaning Predicts Suicidality in Youth Independently of Depression. Crisis: Journal of Crisis Intervention & Suicide. 2018;39(4):294-303.

169. Schwartz-Mette RA, Lawrence HR. Peer Socialization of Non-Suicidal Self-Injury in Adolescents’ Close Friendships. Journal of Abnormal Child Psychology. 2019;47(11):1851-62.

170. Sheline KT, Rosen LA. Posttraumatic growth moderates suicide risk among trauma exposed undergraduates. Journal of College Student Development. 2017;58(3):402-12.

171. Shimshock CM, Williams RA, Sullivan BJ. Suicidal thought in the adolescent: exploring the relationship between known risk factors and the presence of suicidal thought. Journal of child and adolescent psychiatric nursing : official publication of the Association of Child and Adolescent Psychiatric Nurses, Inc. 2011;24(4):237-44.

172. Sigurdson JF, Undheim AM, Wallander JL, Lydersen S, Sund AM. The Longitudinal Association of Being Bullied and Gender with Suicide Ideations, Self-Harm, and Suicide Attempts from Adolescence to Young Adulthood: A Cohort Study. Suicide & Life-Threatening Behavior. 2018;48(2):169-82.

173. Singh MK, Angal S, Nimarko A, Tallman M, Hinman K, Zalpuri I, et al. 24.2 Mechanisms and Risk Factors Underlying Adverse Events from Treating Youth with or at Risk for Bipolar Disorder. Journal of the American Academy of Child and Adolescent Psychiatry. 2019;58 (10 Supplement):S335-S6.

174. Singh S, Manjula M, Philip M. Suicidal risk and childhood adversity: A study of Indian college students. Asian Journal of Psychiatry. 2012;5(2):154-9.

175. Sitnik-Warchulska K, Izydorczyk B. Family Patterns and Suicidal and Violent Behavior among Adolescent Girls-Genogram Analysis. International Journal of Environmental Research & Public Health [Electronic Resource]. 2018;15(10):20.

176. Snir A, Apter A, Barzilay S, Feldman D, Rafaeli E, Carli V, et al. Explicit Motives, Antecedents, and Consequences of Direct Self-Injurious Behaviors. Crisis: Journal of Crisis Intervention & Suicide. 2018;39(4):255-66.

177. Stanford S, Jones MP, Hudson JL. Rethinking pathology in adolescent self-harm: Towards a more complex understanding of risk factors. Journal of Adolescence. 2017;54:32-41.

178. Stansfeld SA, Clark C, Smuk M, Power C, Davidson T, Rodgers B. Childhood adversity and midlife suicidal ideation. Psychological Medicine. 2017;47(2):327-40.

179. Stenager K, Qin P. Individual and parental psychiatric history and risk for suicide among adolescents and young adults in Denmark: a population-based study. Social psychiatry and psychiatric epidemiology. 2008;43(11):920-6.

180. Stevens D, Wilcox HC, MacKinnon DF, Mondimore FM, Schweizer B, Jancic D, et al. Posttraumatic stress disorder increases risk for suicide attempt in adults with recurrent major depression. Depression and Anxiety. 2013;30(10):940-6.

181. Syed Sheriff R, Van Hooff M, Malhi G, Grace B, McFarlane A. Childhood determinants of suicidality: comparing males in military and civilian employed populations. Psychological Medicine. 2018:1-11.

182. Szanto K, Prigerson H, Houck P, Ehrenpreis L, Reynolds ICF. Suicidal ideation in elderly bereaved: The role of complicated grief. Suicide and Life-Threatening Behavior. 1997;27(2):194-204.

183. Tan L, Xia T, Reece C. Social and individual risk factors for suicide ideation among Chinese children and adolescents: A multilevel analysis. International Journal of Psychology. 2018;53(2):117-25.

184. Targum SD, Nemeroff CB. The effect of early life stress on adult psychiatric disorders. Innovations in Clinical Neuroscience. 2019;16(1-2):35-7.

185. Thompson MP, Kingree JB, Lamis D. Associations of adverse childhood experiences and suicidal behaviors in adulthood in a U.S. nationally representative sample. Child: Care, Health & Development. 2019;45(1):121-8.

186. Tschan T, Ludtke J, Schmid M, In-Albon T. Sibling relationships of female adolescents with nonsuicidal self-injury disorder in comparison to a clinical and a nonclinical control group. Child Adolesc Psychiatry Ment Health. 2019;13:15.

187. Vaillant GE, Orav J, Meyer SE, Vaillant LM, Roston D. 1995 IPA/Bayer research awards in psychogeriatrics: Late-life consequences of affective spectrum disorder. International Psychogeriatrics. 1996;8(1):13-32.

188. Valdivia M, Silva D, Sanhueza F, Cova F, Melipillan R. Suicide attempts among Chilean adolescents. Revista medica de Chile. 2015;143(3):320-8.

189. Van Hoek G, Portzky M, Franck E. The influence of socio-demographic factors, resilience and stress reducing activities on academic outcomes of undergraduate nursing students: A cross-sectional research study. Nurse Education Today. 2019;72:90-6.

190. Van Meter AR, Algorta GP, Youngstrom EA, Lechtman Y, Youngstrom JK, Feeny NC, et al. Assessing for suicidal behavior in youth using the Achenbach System of Empirically Based Assessment. European Child & Adolescent Psychiatry. 2018;27(2):159-69.

191. Van Meter AR, Paksarian D, Merikangas KR. Social Functioning and Suicide Risk in a Community Sample of Adolescents. Journal of Clinical Child & Adolescent Psychology. 2019;48(2):273-87.

192. Wadman R, Hiller RM, St Clair MC. The influence of early familial adversity on adolescent risk behaviors and mental health: Stability and transition in family adversity profiles in a cohort sample. Development and Psychopathology. 2019:No Pagination Specified.

193. Waldrop AE, Hanson RF, Resnick HS, Kilpatrick DG, Naugle AE, Saunders BE. Risk factors for suicidal behavior among a national sample of adolescents: Implications for prevention. Journal of Traumatic Stress. 2007;20(5):869-79.

194. Warrier V, Baron-Cohen S. Childhood trauma, life-time self-harm, and suicidal behaviour and ideation are associated with polygenic scores for autism. Molecular Psychiatry. 2019:No Pagination Specified.

195. Webb RT, Pickles AR, Appleby L, Mortensen PB, Abel KM. Death by unnatural causes during childhood and early adulthood in offspring of psychiatric inpatients. Archives of General Psychiatry. 2007;64(3):345-52.

196. Weissman MM, Berry OO, Warner V, Gameroff MJ, Skipper J, Talati A, et al. A 30-year study of 3 generations at high risk and low risk for depression. JAMA Psychiatry. 2016;73(9):970-7.

197. Weller EB, Weller RA, Fristad MA, Bowes JM. Dexamethasone suppression test and depressive symptoms in bereaved children: A preliminary report. Journal of Neuropsychiatry and Clinical Neurosciences. 1990;2(4):418-21.

198. Wijngaards-De Meij L, Stroebe M, Stroebe W, Schut H, Van Den Bout J, Van Der Heijden PGM, et al. The impact of circumstances surrounding the death of a child on parents' grief. Death Studies. 2008;32(3):237-52.

199. Wilcox HC, Storr CL, Breslau N. Posttraumatic stress disorder and suicide attempts in a community sample of urban American young adults. Archives of General Psychiatry. 2009;66(3):305-11.

200. Williamson DE, Ryan ND, Birmaher B, Dahl RE, Kaufman J, Rao U, et al. A case-control family history study of depression in adolescents. Journal of the American Academy of Child and Adolescent Psychiatry. 1995;34(12):1596-607.

201. Woods-Jaeger B, Briggs EC, Vivrette RL, Lee RC, Suarez L, Belcher HME. The Association between Caregiver Substance Abuse and Mental Health Problems and Outcomes for Trauma-Exposed Youth. Journal of Child & Adolescent Trauma. 2019;12(4):447-56.

202. Yamall Orellana JD, de Souza CC, Ponte de Souza ML. Hidden Suicides of the Indigenous People of the Brazilian Amazon: Gender, Alcohol and Familial Clustering. Revista Colombiana de Psiquiatria. 2018.

203. Yamall Orellana JD, de Souza CC, Ponte de Souza ML. Hidden Suicides of the Indigenous People of the Brazilian Amazon: Gender, Alcohol and Familial Clustering. Revista Colombiana de Psiquiatria. 2019;48(3):133-9.

204. Youngblut JM, Brooten D, Blais K, Hannan J, Niyonsenga T. Grandparent's Health and Functioning After a Grandchild's Death. Journal of Pediatric Nursing. 2010;25(5):352-9.

205. Zall DS. The Long Term Effects of Childhood Bereavement: Impact on Roles as Mothers. Omega. 1994;29(3):219-30.

206. Zhang J, Stewart R, Phillips M, Shi Q, Prince M. Pesticide exposure and suicidal ideation in rural communities in Zhejiang province, China. Bulletin of the World Health Organization. 2009;87(10):746-53.

207. Zisook S, Lyons L. Bereavement and Unresolved Grief in Psychiatric Outpatients. Omega. 1990;20(4):307-22.

208. Zwald ML, Annor FB, Wilkinson A, Friedrichs M, Fondario A, Dunn AC, et al. Suicidal Ideation and Attempts Among Students in Grades 8, 10, and 12 - Utah, 2015. MMWR - Morbidity & Mortality Weekly Report. 2018;67(15):451-4.

## Outcomes not eligible

1. Appel CW, Johansen C, Deltour I, Frederiksen K, Hjalgrim H, Dalton SO, et al. Early parental death and risk of hospitalization for affective disorder in adulthood. Epidemiology. 2013;24(4):608-15.

2. Aronson KR, Kyler SJ, Morgan NR, Perkins DF, Love L. Spouse and family functioning before and after a Marine's suicide: Comparisons to deaths by accident and in combat. Military Psychology (American Psychological Association). 2017;29(4):294-306.

3. Baddeley JL, Williams JL, Rynearson T, Correa F, Saindon C, Rheingold AA. Death Thoughts and Images in Treatment-Seekers After Violent Loss. Death Studies. 2015;39(2):84-91.

4. Bailley SE, Dunham K, Kral MJ. Factor structure of The Grief Experience Questionnaire (GEQ). Death Studies. 2000;24(8):721-38.

5. Bailley SE, Kral MJ, Dunham K. Survivors of suicide do grieve differently: empirical support for a common sense proposition. Suicide & life-threatening behavior. 1999;29(3):256-71.

6. Barrett TW. The survivors: A comparison of bereavement experiences following the suicide, accidental, or natural death of a spouse. Dissertation Abstracts International. 1990;50(9-B):4210.

7. Barrett TW, Scott TB. Suicide bereavement and recovery patterns compared with nonsuicide bereavement patterns. Suicide and Life-Threatening Behavior. 1990;20(1):1-15.

8. Beristianos MH, Maguen S, Neylan TC, Byers AL. TRAUMA EXPOSURE AND RISK OF SUICIDAL IDEATION AMONG ETHNICALLY DIVERSE ADULTS. Depression and Anxiety. 2016;33(6):495-501.

9. Bernburg JG, Thorlindsson T, Sigfusdottir ID. The spreading of suicidal behavior: The contextual effect of community household poverty on adolescent suicidal behavior and the mediating role of suicide suggestion. Social science & medicine (1982). 2009;68(2):380-9.

10. Bjorkenstam E, Kosidou K, Bjorkenstam C. Childhood household dysfunction and risk of self-harm: A cohort study of 107 518 young adults in Stockholm County. International Journal of Epidemiology. 2016;45(2):501-11.

11. Bolton JM, Au W, Leslie WD, Martens PJ, Enns MW, Roos LL, et al. Parents bereaved by offspring suicide: A population-based longitudinal case-control study. JAMA Psychiatry. 2013;70(2):158-67.

12. Bottomley JS, Abrutyn S, Smigelsky MA, Neimeyer RA. Mental Health Symptomatology and Exposure to Non-Fatal Suicidal Behavior: Factors That Predict Vulnerability and Resilience Among College Students. Archives of suicide research : official journal of the International Academy for Suicide Research. 2017:1-19.

13. Bottomley JS, Abrutyn S, Smigelsky MA, Neimeyer RA. Exposure to Nonfatal Suicidal Behavior: Examining Pathways to Suicide Risk Using the Interpersonal-Psychological Theory of Suicide (IPTS). Journal of Loss & Trauma. 2019;24(3):261-78.

14. Brent D, Melhem N, Donohoe MB, Walker M. The incidence and course of depression in bereaved youth 21 months after the loss of a parent to suicide, accident, or sudden natural death. American Journal of Psychiatry. 2009;166(7):786-94.

15. Brent DA, Melhem NM, Masten AS, Porta G, Payne MW. Longitudinal Effects of Parental Bereavement on Adolescent Developmental Competence. Journal of Clinical Child and Adolescent Psychology. 2012;41(6):778-91.

16. Brent DA, Moritz G, Liotus L, Schweers J, Balach L, Roth C, et al. Familial risk factors for adolescent suicide: A case-control study. Suicide prevention: The global context. 1998:41-50.

17. Brent DA, Oquendo M, Birmaher B, Greenhill L, et al. Familial transmission of mood disorders: Convergence and divergence with transmission of suicidal behavior. Journal of the American Academy of Child and Adolescent Psychiatry. 2004;43(10):1259-66.

18. Brent DA, Perper J, Moritz G, Friend A, et al. Adolescent witnesses to a peer suicide. Journal of the American Academy of Child and Adolescent Psychiatry. 1993;32(6):1184.

19. Brent DA, Perper JA, Moritz G, Allman C, et al. Psychiatric sequelae to the loss of an adolescent peer to suicide. Journal of the American Academy of Child and Adolescent Psychiatry. 1993;32(3):509.

20. Bridge JA. Major depressive disorder among youth exposed to a friend's suicide. Dissertation Abstracts International: Section B: The Sciences and Engineering. 2002;62(9-B):3966.

21. Bridge JA, Brent DA, Johnson BA, Connolly J. Familial aggregation of psychiatric disorders in a community sample of adolescents. Journal of the American Academy of Child and Adolescent Psychiatry. 1997;36(5):628-36.

22. Bridge JA, Day NL, Day R, Richardson GA, et al. Major depressive disorder in adolescents exposed to a friend's suicide. Journal of the American Academy of Child and Adolescent Psychiatry. 2003;42(11):1294-300.

23. Brown AC, Sandler IN, Tein JY, Liu X, Haine RA. Implications of parental suicide and violent death for promotion of resilience of parentally-bereaved children. Death Studies. 2007;31(4):301-35.

24. Cerel J, Bolin MC, Moore MM. Suicide exposure, awareness and attitudes in college students. Advances in Mental Health. 2013;12(1):46-53.

25. Cerel J, Fristad MA, Weller EB, Weller RA. Suicide-bereaved children and adolescents: A controlled longitudinal examination. Journal of the American Academy of Child and Adolescent Psychiatry. 1999;38(6):672-9.

26. Cerel J, Fristad MA, Weller EB, Weller RA. Suicide-bereaved children and adolescents: II. Parental and family functioning. Journal of the American Academy of Child and Adolescent Psychiatry. 2000;39(4):437-44.

27. Chan WI, Batterham P, Christensen H, Galletly C. Suicide literacy, suicide stigma and help-seeking intentions in Australian medical students. Australasian Psychiatry. 2014;22(2):132-9.

28. Cheung T, Yip PSF. Self-harm in nurses: prevalence and correlates. Journal of Advanced Nursing. 2016;72(9):2124-37.

29. Clark SE, Goldney RD. Grief reactions and recovery in a support group for people bereaved by suicide. Crisis. 1995;16(1):27-33.

30. Cleiren MPHD, Grad O, Zavasnik A, Diekstra RFW. Psychosocial impact of bereavement after suicide and fatal traffic accident: A comparative two-country study. Acta Psychiatrica Scandinavica. 1996;94(1):37-44.

31. Constantino RE, Sekula LK, Lebish J, Buehner E. Depression and behavioral manifestation of depression in female survivors of the suicide of their significant other and female survivors of abuse. Journal of the American Psychiatric Nurses Association. 2002;8(1):27-32.

32. Cooper E, Driedger S, Lavoie JG. Employing a harm-reduction approach between women and girls within indigenous familial relationships. Culture, Medicine, and Psychiatry: An International Journal of Cross-Cultural Health Research. 2019;43(1):134-59.

33. Cox LJ, Stanley BH, Melhem NM, Oquendo MA, Birmaher B, Burke A, et al. Familial and individual correlates of nonsuicidal self-injury in the offspring of mood-disordered parents. Journal of Clinical Psychiatry. 2012;73(6):813-20.

34. Cox LJ, Stanley BH, Melhem NM, Oquendo MA, Birmaher B, Burke A, et al. A longitudinal study of nonsuicidal self-injury in offspring at high risk for mood disorder. Journal of Clinical Psychiatry. 2012;73(6):821-8.

35. Csorba J, Rozsa S, Vetro A, Gadoros J, Makra J, Somogyi E, et al. Family- and school-related stresses in depressed Hungarian children. European Psychiatry. 2001;16(1):18-26.

36. Currier JM, Holland JM, Neimeyer RA. Sense-making, grief, and the experience of violent loss: Toward a mediational model. Death Studies. 2006;30(5):403-28.

37. Deliberto TL, Nock MK. An Exploratory Study of Correlates, Onset, and Offset of Non-Suicidal Self-Injury. Archives of Suicide Research. 2008;12(3):219-31.

38. Demi AM. Adjustment to widowhood after a sudden death: Suicide and non-suicide survivors compared. Dissertation Abstracts International. 1978;38(12-B):5847-8.

39. Demi AS. Social adjustment of widows after a sudden death: Suicide and non-suicide survivors compared. Special Issue: Suicide--practical, developmental, and speculative issues. 1984;8(Suppl):91-111.

40. Demi AS, Miles MS. Suicide bereaved parents: Emotional distress and physical health problems. Death Studies. 1988;12(4):297-307.

41. Du C-J, Li X-Y, Zhang Y-P. Comparison of the characteristics of completed suicide in people with and without mental disorders older than 55 and with no serious physical illnesses. Chinese Mental Health Journal. 2008;22(6):397-400.

42. Dyregrov K. Micro-sociological analysis of social support following traumatic bereavement: Unhelpful and avoidant responses from the community. Omega. 2003;48(1):23-44.

43. Dyregrov K, Nordanger D, Dyregrov A. Predictors of psychosocial distress after suicide, sids and accidents. Death Studies. 2003;27(2):143-65.

44. Edwards AC, Ohlsson H, Moscicki EK, Sundquist J, Sundquist K, Kendler KS. Geographic proximity is associated with transmission of suicidal behavior among siblings. Acta Psychiatrica Scandinavica. 2019:18.

45. Erlangsen A, Runeson B, Bolton JM, Wilcox HC, Forman JL, Krogh J, et al. Association between spousal suicide and mental, physical, and social health outcomes a longitudinal and nationwide register-based study. JAMA Psychiatry. 2017;74(5):456-64.

46. Eskin M. Suicidal behavior as related to social support and assertiveness among Swedish and Turkish high school students: a cross-cultural investigation. Journal of clinical psychology. 1995;51(2):158-72.

47. Farberow NL, Gallagher DE, Gilewski MJ, Thompson LW. An Examination of the Early Impact of Bereavement on Psychological Distress in Survivors of Suicide. The Gerontologist. 1987;27(5):592.

48. Farberow NL, Gallagher-Thompson D, Gilewski M, Thompson L. Changes in grief and mental health of bereaved spouses of older suicides. Journals of Gerontology. 1992;47(6):P357-P66.

49. Farberow NL, Gallagher-Thompson D, Gilewski M, Thompson L. The Role of Social Supports in the Bereavement Process of Surviving Spouses of Suicide and Natural Deaths. Suicide and Life-Threatening Behavior. 1992;22(1):107-24.

50. Feigelman W, Cerel J, McIntosh JL, Brent D, Gutin N. Suicide exposures and bereavement among American adults: Evidence from the 2016 General Social Survey. Journal of Affective Disorders. 2017;227:1-6.

51. Feigelman W, Cerel J, McIntosh JL, Brent D, Gutin N. Suicide exposures and bereavement among American adults: Evidence from the 2016 General Social Survey. Journal of Affective Disorders. 2018;227:1-6.

52. Feigelman W, Jordan JR, Gorman BS. How They Died, Time Since Loss, and Bereavement Outcomes. Omega. 2008;58(4):251.

53. Feigelman W, Jordan JR, Gorman BS. Parental Grief After a Child's Drug Death Compared to Other Death Causes: Investigating a Greatly Neglected Bereavement Population. Omega. 2011;63(4):291.

54. Feigelman W, McIntosh J, Cerel J, Brent D, Gutin NJ. Identifying the Social Demographic Correlates of Suicide Bereavement. Archives of Suicide Research. 2019;23(2):273-88.

55. Fergusson DM, Beautrais AL, Horwood LJ. Vulnerability and resiliency to suicidal behaviours in young people. Psychological Medicine. 2003;33(1):61-73.

56. Floyd FJ, Mailick Seltzer M, Greenberg JS, Song J. Parental bereavement during mid-to-later life: Pre- to postbereavement functioning and intrapersonal resources for coping. Psychology and Aging. 2013;28(2):402.

57. Garber J, Little S, Hilsman R, Weaver KR. Family predictors of suicidal symptoms in young adolescents. Journal of Adolescence. 1998;21(4):445-57.

58. Gilewski MJ, Farberow NL, Gallagher DE, Thompson LW. Interaction of depression and bereavement on mental health in the elderly. Psychology and aging. 1991;6(1):67-75.

59. Goldney RD, Spence ND, Moffitt PF. The aftermath of suicide: A comparison of attitudes of those bereaved by suicide, social workers and a community sample. Crisis: The Journal of Crisis Intervention and Suicide Prevention. 1986;7(1):38-43.

60. Goldney RD, Spence ND, Moffitt PF. The aftermath of suicide: Attitudes of those bereaved by suicide, of social workers, and of a community sample. Journal of Community Psychology. 1987;15(2):141-8.

61. Grad OT, Zavasnik A. Similarities and Differences in the Process of Bereavement after Suicide and after Traffic Fatalities in Slovenia. Omega. 1996;33(3):243-51.

62. Grad OT, Zavasnik A. Phenomenology of bereavement process after suicide, traffic accident and terminal illness (in spouses). Archives of Suicide Research. 1999;5(2):157-72.

63. Grivel MM, Leong W, Masucci MD, Altschuler RA, Arndt LY, Redman SL, et al. Impact of lifetime traumatic experiences on suicidality and likelihood of conversion in a cohort of individuals at clinical high-risk for psychosis. Schizophrenia Research. 2018;195:549-53.

64. Gutierrez PM, Rodriguez PJ, Garcia P. Suicide risk factors for young adults: Testing a model across ethnicities. Death Studies. 2001;25(4):319-40.

65. Hanhart E. Contribution of genetic factors in suicide based on temporary increase in the rate of familial suicides in Swiss isolates. Archiv der Julius Klaus-Stiftung fur Vererbungsforschung, Sozialanthropologie und Rassenhygiene. 1968;43-44:305-76.

66. Hanhart E. The question of the involvement of genetic factors in suicide based on temporal clustering of familial suicides in isolated places of Switzerland. Arch Klaus-Stift Vererb Forsch. 1969;43:305-76.

67. Harris KM, Bettiol S. Exposure to suicidal behaviors: A common suicide risk factor or a personal negative life event? The International Journal of Social Psychiatry. 2017;63(1):70-7.

68. Harwood D, Hawton K, Hope T, Jacoby R. The grief experiences and needs of bereaved relatives and friends of older people dying through suicide: A descriptive and case-control study. Journal of Affective Disorders. 2002;72(2):185-94.

69. Hasking P, Andrews T, Martin G. The Role of Exposure to Self-Injury Among Peers in Predicting Later Self-Injury. Journal of Youth and Adolescence. 2013;42(10):1543-56.

70. Helsing KJ, Comstock GW, Szklo M. Causes of death in a widowed population. American Journal of Epidemiology. 1982;116(3):524-32.

71. Herndon BK. Toward the prediction of suicidal behavior: Psychosocial characteristics of adolescent suicide attempters. Dissertation Abstracts International: Section B: The Sciences and Engineering. 1994;55(1-B):6712.

72. Hoeg BL, Johansen C, Christensen J, Frederiksen K, Dalton SO, Dyregrov A, et al. Early parental loss and intimate relationships in adulthood: A nationwide study. Developmental psychology. 2018;54(5):963-74.

73. Hoehne A, Richard-Devantoy S, Ding Y, Turecki G, Jollant F. First-degree relatives of suicide completers may have impaired decision-making but functional cognitive control. Journal of Psychiatric Research. 2015;68:192-7.

74. Hollingshaus MS, Coon H, Crowell SE, Gray DD, Hanson HA, Pimentel R, et al. Differential Vulnerability to Early-Life Parental Death: The Moderating Effects of Family Suicide History on Risks for Major Depression and Substance Abuse in Later Life. Biodemography and Social Biology. 2016;62(1):105.

75. Hunt QA, Young TA, Hertlein KM. The process of long-term suicide bereavement: Responsibility, familial support, and meaning making. Contemporary Family Therapy: An International Journal. 2019:No Pagination Specified.

76. Johansson L, Lindqvist P, Eriksson A. Teenage suicide cluster formation and contagion: Implications for primary care. BMC Family Practice. 2006;7:32.

77. Kaplow JB, Howell KH, Layne CM. Do circumstances of the death matter? Identifying socioenvironmental risks for grief-related psychopathology in bereaved youth. Journal of traumatic stress. 2014;27(1):42-9.

78. Kaprio J, Koskenvuo M, Rita H. Mortality after bereavement: A prospective study of 95,647 widowed persons. American Journal of Public Health. 1987;77(3):283-7.

79. Kebede D, Ketsela T. Suicide attempts in Ethiopian adolescents in Addis Abeba high schools. Ethiopian medical journal. 1993;31(2):83-90.

80. Kennedy B, Chen R, Valdimarsdottir U, Montgomery S, Fang F, Fall K. Childhood Bereavement and Lower Stress Resilience in Late Adolescence. Journal of Adolescent Health. 2018.

81. Kersting A, Brahler E, Glaesmer H, Wagner B. Prevalence of complicated grief in a representative population-based sample. Journal of Affective Disorders. 2011;131(1-3):339-43.

82. Kessing LV, Agerbo E, Mortensen PB. Does the impact of major stressful life events on the risk of developing depression change throughout life? Psychological medicine. 2003;33(7):1177-84.

83. Kitson GC. Adjustment to Violent and Natural Deaths in Later and Earlier Life for Black and White Widows. Journals of Gerontology Series B: Psychological Sciences and Social Sciences. 2000;55B(6):S341-S51.

84. Kolves K, de Leo D. Suicide bereavement: piloting a longitudinal study in Australia. BMJ Open. 2018;8(1):e019504.

85. Kolves K, Zhao Q, Ross V, Hawgood J, Spence SH, de Leo D. Suicide and other sudden death bereavement of immediate family members: An analysis of grief reactions six-months after death. Journal of Affective Disorders. 2019;243:96-102.

86. Kovarsky RS. Loneliness and disturbed grief: a comparison of parents who lost a child to suicide or accidental death. Archives of psychiatric nursing. 1989;3(2):86-96.

87. Kwon H-K, Rueter MA. Developmental trends in adolescent suicidal ideation. Journal of Research on Adolescence. 2005;15(2):205-22.

88. Lee E, Kim Sw, Enright RD. Beyond Grief and Survival. Omega. 2019;79(4):414-35.

89. Levi-Belz Y. To share or not to share? The contribution of self-disclosure to stress-related growth among suicide survivors. Death studies. 2016;40(I7):405-13.

90. Liang H, Olsen J, Yuan W, Cnattingus S, Vestergaard M, Obel C, et al. Early life bereavement and schizophrenia: A nationwide cohort study in Denmark and Sweden. Medicine (United States). 2016;95(3):e2434.

91. Lichtenthal WG, Neimeyer RA, Currier JM, Roberts K, Jordan N. Cause of Death and the Quest for Meaning After the Loss of a Child. Death Studies. 2013;37(4):311-42.

92. Ljung T, Sandin S, Langstrom N, Runeson B, Lichtenstein P, Larsson H. Offspring death and subsequent psychiatric morbidity in bereaved parents: addressing mechanisms in a total population cohort. Psychological Medicine. 2014;44(9):1879-87.

93. Lohan JA, Murphy SA. Family functioning and family typology after an adolescent or young adult's sudden violent death. Journal of Family Nursing. 2002;8(1):32-49.

94. Maciejewski DF, Renteria ME, Abdellaoui A, Medland SE, Few LR, Gordon SD, et al. The Association of Genetic Predisposition to Depressive Symptoms with Non-suicidal and Suicidal Self-Injuries. Behavior Genetics. 2017;47(1):3-10.

95. McIntosh JL, Kelly LD. Survivors' reactions: Suicide vs. other causes. Crisis: The Journal of Crisis Intervention and Suicide Prevention. 1992;13(2):82-93.

96. McLaughlin KA, Gadermann AM, Hwang I, Sampson NA, Al-Hamzawi A, Andrade LH, et al. Parent psychopathology and offspring mental disorders: results from the WHO World Mental Health Surveys. The British Journal of Psychiatry. 2012;200(4):290-9.

97. McManama O'Brien KH, Salas-Wright CP, Vaughn MG, LeCloux M. Childhood exposure to a parental suicide attempt and risk for substance use disorders. Addictive Behaviors. 2015;46:70-6.

98. McNiel DE, Hatcher C, Reubin R. Family survivors of suicide and accidental death: Consequences for widows. Suicide and Life-Threatening Behavior. 1988;18(2):137-48.

99. Medina CMO, Herrera A, Kullgren G. Suicidal expression in adolescents in Nicaragua in relation to Youth Self-Report (YSR) syndromes and exposure to suicide. Clinical Practice and Epidemiology in Mental Health. 2011;7:89-96.

100. Melhem NM, Porta G, Shamseddeen W, Walker Payne M, Brent DA. Grief in children and adolescents bereaved by sudden parental death. Archives of General Psychiatry. 2011;68(9):911-9.

101. Miles MS, Demi AS. A Comparison of Guilt in Bereaved Parents whose Children Died by Suicide, Accident, or Chronic Disease. Omega. 1992;24(3):203-15.

102. Miyabayashi S, Yasuda J. Effects of loss from suicide, accidents, acute illness and chronic illness on bereaved spouses and parents in Japan: Their general health, depressive mood, and grief reaction. Psychiatry and Clinical Neurosciences. 2007;61(5):502-8.

103. Mok PLH, Pedersen CB, Springate D, Astrup A, Kapur N, Antonsen S, et al. Parental psychiatric disease and risks of attempted suicide and violent criminal offending in offspring a population-based cohort study. JAMA Psychiatry. 2016;73(10):1015-22.

104. Murphy SA, Johnson LC, Wu L, Fan JJ, Lohan J. Bereaved parents' outcomes 4 to 60 months after their children's deaths by accident, suicide, or homicide: A comparative study demonstrating differences. Death Studies. 2003;27(1):39-61.

105. Nelson BJ, Frantz TT. Family interactions of suicide survivors and survivors of non-suicidal death. Omega: Journal of Death and Dying. 1996;33(2):131-46.

106. Ogata K, Ishikawa T, Michiue T, Nishi Y, Maeda H. Posttraumatic Symptoms in Japanese Bereaved Family Members with Special Regard to Suicide and Homicide Cases. Death Studies. 2011;35(6):525-35.

107. Omerov P, Steineck G, Nyberg T, Runeson B, Nyberg U. Psychological morbidity among suicide-bereaved and non-bereaved parents: a nationwide population survey. BMJ open. 2013;3(8):e003108.

108. Pedraza RS, Guzman Y, Rubio HC. Study of Imitation as a Risk Factor for Suicidal Ideation in University Adolescent Students. Estudio de la imitacion como factor de riesgo para ideacion suicida en estudiantes universitarios adolescentes. 2005;34(1[41], 41):12-25.

109. Pirelli G, Jeglic EL. The Influence of Death Exposure on Suicidal Thoughts and Behaviors. Archives of Suicide Research. 2009;13(2):136-46.

110. Pitman A, Osborn D, King M. Suicide bereavement and risk for suicide attempt: A national cross-sectional survey of young adults. The Lancet. 2014;383(SUPPL. 1):S82.

111. Pitman A, Rantell K, Marston L, King M, Osborn D. Perceived stigma of sudden bereavement as a risk factor for suicidal thoughts and suicide attempt: Analysis of British cross-sectional survey data on 3387 young bereaved adults. International Journal of Environmental Research and Public Health. 2017;14(3):286.

112. Pitman AL, Osborn DPJ, Rantell K, King MB. The stigma perceived by people bereaved by suicide and other sudden deaths: A cross-sectional UK study of 3432 bereaved adults. Journal of Psychosomatic Research. 2016;87:22-9.

113. Pitman AL, Rantell K, Moran P, Sireling L, Marston L, King M, et al. Support received after bereavement by suicide and other sudden deaths: A cross-sectional UK study of 3432 young bereaved adults. BMJ Open. 2017;7(5):e014487.

114. Portzky G, Audenaert K, Van Heeringen K. Psychosocial and psychiatric factors associated with adolescent suicide: A case-control psychological autopsy study. Journal of Adolescence. 2009;32(4):849-62.

115. Range LM, Calhoun LG. Responses following Suicide and Other Types of Death: The Perspective of the Bereaved. Omega. 1990;21(4):311-20.

116. Range LM, Niss NM. Long-term bereavement from suicide, homicide, accidents, and natural deaths. Death Studies. 1990;14(5):423-33.

117. Reed MD. Predicting grief symptomatology among the suddenly bereaved. Suicide and Life-Threatening Behavior. 1998;28(3):285-301.

118. Reed MD, Greenwald JY. Survivor-victim status, attachment, and sudden death bereavement. Suicide and Life-Threatening Behavior. 1991;21(4):385-401.

119. Reyes-Portillo JA, Lake AM, Kleinman M, Gould MS. The Relation between Descriptive Norms, Suicide Ideation, and Suicide Attempts among Adolescents. Suicide & life-threatening behavior. 2018.

120. Rohanachandra YM, Prathapan S, Wijetunge GS. Characteristics of mothers' depressive illness as predictors for emotional and behavioural problems in children in a Sri Lankan setting. Asian Journal of Psychiatry. 2018;33:74-7.

121. Rostila M, Saarela J, Kawachi I. Mortality in parents following the death of a child: A nationwide follow-up study from Sweden. Journal of Epidemiology and Community Health. 2012;66(10):927-33.

122. Rostila M, Saarela J, Kawachi I. Suicide following the death of a sibling: A nationwide follow-up study from Sweden. BMJ Open. 2013;3(4):e002618.

123. Roy A. Family history of suicide and neuroticism: A preliminary study. Psychiatry Research. 2002;110(1):87-90.

124. Roze M, Vandentorren S, Vuillermoz C, Chauvin P, Melchior M. Emotional and behavioral difficulties in children growing up homeless in Paris. Results of the ENFAMS survey. European psychiatry : the journal of the Association of European Psychiatrists. 2016;38:51-60.

125. Santos S, Campos RC, Tavares S. Suicidal ideation and distress in family members bereaved by suicide in Portugal. Death studies. 2015;39(6):332-41.

126. Seguin M, Lesage A, Kiely M. History of early loss among a group of suicide survivors. Crisis. 1995;16(3):121-5.

127. Seguin M, Lesage A, Kiely MC. Parental bereavement after suicide and accident: A comparative study. Suicide and Life-Threatening Behavior. 1995;25(4):489-98.

128. Seponski DM, Somo CM, Kao S, Lahar CJ, Khann S, Schunert T. Family, health, and poverty factors impacting suicide attempts in Cambodian women: A qualitative analysis from a randomly selected national sample. Crisis: The Journal of Crisis Intervention and Suicide Prevention. 2019;40(2):141-5.

129. Sethi S, Bhargava SC. Child and adolescent survivors of suicide. Crisis. 2003;24(1):4-6.

130. Shanahan L, Schorpp KM, Volpe VV, Linthicum K, Freeman JA. Developmental timing of suicide attempts and cardiovascular risk during young adulthood. Health Psychology. 2016;35(10):1135.

131. Sharpe TL, Joe S, Taylor KC. Suicide and homicide bereavement among African Americans: Implications for survivor research and practice. Omega (United States). 2012;66(2):153-72.

132. Shepherd D, Barraclough BM. The aftermath of suicide. British medical journal. 1974;2(5919):600-3.

133. Shepherd DM, Barraclough BM. The aftermath of parental suicide for children. British Journal of Psychiatry. 1976;129(9):267-76.

134. Silverman E, Range L, Overholser J. Bereavement from suicide as compared to other forms of bereavement. Omega: Journal of Death and Dying. 1995;30(1):41-51.

135. Stanford S, Jones MP, Hudson JL. Appreciating Complexity in Adolescent Self-Harm Risk Factors: Psychological Profiling in a Longitudinal Community Sample. Journal of youth and adolescence. 2018;47(5):916-31.

136. Stein D, Witztum E, Brom D, DeNour AK, Elizur A. The association between adolescents' attitudes toward suicide and their psychosocial background and suicidal tendencies. Adolescence. 1992;27(108):949-59.

137. Thompson KE, Range LM. Recent Bereavement from Suicide and other Deaths: Can People Imagine it as it Really is? Omega. 1991;22(4):249-59.

138. Thompson KE, Range LM. Bereavement following Suicide and Other Deaths: Why Support Attempts Fail. Omega. 1992;26(1):61-70.

139. Thompson M, Kuruwita C, Foster EM. Transitions in Suicide Risk in a Nationally Representative Sample of Adolescents. Journal of Adolescent Health. 2009;44(5):458-63.

140. Thompson MP, Swartout K. Epidemiology of Suicide Attempts among Youth Transitioning to Adulthood. Journal of Youth and Adolescence. 2018;47(4):807-17.

141. Torzsa P, Rihmer Z, Gonda X, Szokontor N, Sebestyen B, Faludi G, et al. Family history of suicide: A clinical marker for major depression in primary care practice? Journal of Affective Disorders. 2009;117(3):202-4.

142. Tsuchiya KJ, Agerbo E, Mortensen PB. Parental death and bipolar disorder: A robust association was found in early maternal suicide. Journal of Affective Disorders. 2005;86(2-3):151-9.

143. Ugurlu N, Ona N. Relationship Between the Stress-Coping Levels of University Students and their Probability of Committing Suicide. Social Behavior and Personality. 2009;37(9):1221-30.

144. von Borczyskowski A, Lindblad F, Vinnerljung B, Reintjes R, Hjern A. Familial factors and suicide: an adoption study in a Swedish National Cohort. Psychological Medicine. 2011;41(4):749-58.

145. Wagner KG, Calhoun LG. Perceptions of Social Support by Suicide Survivors and their Social Networks. Omega. 1992;24(1):61-73.

146. Whalen DJP, Dixon-Gordon KP, Belden ACP, Barch DP, Luby JLMD. Correlates and Consequences of Suicidal Cognitions and Behaviors in Children Ages 3 to 7 Years. Journal of the American Academy of Child and Adolescent Psychiatry. 2015;54(11):926.

147. Wienforth J. [Suicidal behavior and transmission of death experiences in the family]. Suizidalitat und Weitergabe von Todeserlebnissen in der Familie. 1985;31(4):365-79.

148. Wilcox HC, Kuramoto SJ, Brent D, Runeson B. The interaction of parental history of suicidal behavior and exposure to adoptive parents' psychiatric disorders on adoptee suicide attempt hospitalizations. American Journal of Psychiatry. 2012;169(3):309-15.

149. Wilcox HC, Mittendorfer-Rutz E, Kjeldgard L, Alexanderson K, Runeson B. Functional impairment due to bereavement after the death of adolescent or young adult offspring in a national population study of 1,051,515 parents. Social psychiatry and psychiatric epidemiology. 2015;50(8):1249-56.

150. Xu G, Li N. A comparison study on mental health status between suicide survivors and survivors of accidental deaths in rural China. Journal of psychiatric and mental health nursing. 2014;21(10):859-65.

151. Yang L, Zhang Z, Sun L, Wu H, Sun Y. [Risk and risk factors of suicide attempt after first onset of suicide ideation: findings from medical students in grades 1 and 2]. Wei sheng yan jiu = Journal of hygiene research. 2014;43(1):47-53.

152. Zammit S, Gunnell D, Lewis G, Leckie G, Dalman C, Allebeck P. Individual- and area-level influence on suicide risk: A multilevel longitudinal study of Swedish schoolchildren. Psychological Medicine. 2014;44(2):267-77.

153. Zelkowitz RL, Porter AC, Heiman ER, Cole DA. Social exposure and emotion dysregulation: Main effects in relation to nonsuicidal self-injury. Journal of Adolescence. 2017;60:94.

154. Zhang J, Hui Qi T, Zhou L. THE EFFECT OF BEREAVEMENT DUE TO SUICIDE ON SURVIVORS' DEPRESSION: A STUDY OF CHINESE SAMPLES. Omega. 2005;51(3):217-27.

155. Zhang J, Jia CX. Attitudes toward suicide: The effect of suicide death in the family. Omega: Journal of Death and Dying. 2010;60(4):365-82.

156. Zimmerman GM, Rees C, Posick C, Zimmerman LA. The power of (Mis)perception: Rethinking suicide contagion in youth friendship networks. Social Science & Medicine. 2016;157:31.

157. Zisook S, Chentsova-Dutton Y, Shuchter SR. PTSD following bereavement. Annals of Clinical Psychiatry. 1998;10(4):157-63.

## No control/comparison group or control/comparison group not eligible

1. Aarab C, Elghazouani F, Aalouane R, Rammouz I. 5-year prospective follow-up suicide attempts in clinical population in the region of Fez, Morocco. Pan African Medical Journal. 2014;18:321.

2. Aas M, Henry C, Bellivier F, Lajnef M, Gard S, Kahn JP, et al. Affective lability mediates the association between childhood trauma and suicide attempts, mixed episodes and co-morbid anxiety disorders in bipolar disorders. Psychological Medicine. 2017;47(5):902-12.

3. Abbott CH, Zakriski AL. Grief and Attitudes Toward Suicide in Peers Affected by a Cluster of Suicides as Adolescents. Suicide and Life-Threatening Behavior. 2014;44(6):668.

4. Abe K, Mertz KJ, Powell KE, Hanzlick RL. Characteristics of Black and White suicide decedents in Fulton County, Georgia 1988-2002. American Journal of Public Health. 2006;96(10):1794-8.

5. Adams E, Hawgood J, Bundock A, Kolves K. A phenomenological study of siblings bereaved by suicide: A shared experience. Death Studies. 2019;43(5):324-32.

6. Agerbo E. Midlife suicide risk, partner's psychiatric illness, spouse and child bereavement by suicide or other modes of death: A gender specific study. Journal of Epidemiology and Community Health. 2005;59(5):407-12.

7. Allen BG, Calhoun LG, Cann A, Tedeschi RG. The effect of cause of death on responses to the bereaved: Suicide compared to accident and natural causes. Omega: Journal of Death and Dying. 1993;28(1):39-48.

8. Annor FB, Zwald ML, Wilkinson A, Friedrichs M, Fondario A, Dunn A, et al. Characteristics of and Precipitating Circumstances Surrounding Suicide Among Persons Aged 10-17 Years - Utah, 2011-2015. MMWR - Morbidity & Mortality Weekly Report. 2018;67(11):329-32.

9. Anonymous. Adolescent suicide and suicide attempts--Santa Fe County, New Mexico, January 1985-May 1990. MMWR Morbidity and mortality weekly report. 1991;40(20):329-31.

10. Asare-Doku W, Osafo J, Akotia CS. Comparing the reasons for suicide from attempt survivors and their families in Ghana. BMC Public Health. 2019;19(1):412.

11. Balestri L, Giordano PL. Study of the family atmosphere as it affects attempted suicides during adolescence. Giornale di psichiatria e di neuropatologia. 1966;94(2):325-36.

12. Bansal PD, Barman R. Psychiatric morbidity and the socio-demographic determinants of deliberate self harm. Journal of Clinical and Diagnostic Research. 2011;5(3):601-4.

13. Beautrais AL. Suicide and serious suicide attempts in youth: a multiple-group comparison study. American Journal of Psychiatry. 2003;160(6):1093-9.

14. Benedek CDM. Impact of soldier suicide on unit and care givers: Implications for education and training. Psychiatry. 2011;74(2):124-6.

15. Bhatia MS, Aggarwal NK, Aggarwal BBL. Psychosocial profile of suicide ideators, attempters and completers in India. International Journal of Social Psychiatry. 2000;46(3):155-63.

16. Bhushan B, Kumar S, Harizuka S. Bereavement, Cognitive-Emotional Processing, and Coping With the Loss: A Study of Indian and Japanese Students. Journal of Social Work in End-of-Life & Palliative Care. 2011;7(2-3):263-80.

17. Biddle L. Public hazards or private tragedies? an exploratory study of the effect of coroners' procedures on those bereaved by suicide. Social Science and Medicine. 2003;56(5):1033-45.

18. Blasco-Fontecilla H, Jaussent I, Olie E, Garcia EB, Beziat S, Malafosse A, et al. Additive effects between prematurity and postnatal risk factors of suicidal behavior. Journal of Psychiatric Research. 2013;47(7):937-43.

19. Brent D, Day N, Day R, Melhem NM, Reynolds Iii CF, Shear MK. Traumatic grief among adolescents exposed to a peer's suicide. American Journal of Psychiatry. 2004;161(8):1411-6.

20. Brent DA, Kerr MM, Goldstein C, Bozigar J, et al. An outbreak of suicide and suicidal behavior in a high school. Journal of the American Academy of Child and Adolescent Psychiatry. 1989;28(6):918.

21. Brent DA, Kerr MM, Goldstein C, Bozigar J, Wartella M, Allan MJ. An outbreak of suicide and suicidal behavior in a high school. Annual progress in child psychiatry and child development, 1990. 1991:532-46.

22. Brent DA, Perper J, Moritz G, Allman C, Liotus L, Schweers J, et al. Bereavement or depression? The impact of the loss of a friend to suicide. Journal of the American Academy of Child and Adolescent Psychiatry. 1993;32(6):1189-97.

23. Burrell LV, Mehlum L, Qin P. Risk factors for suicide in offspring bereaved by sudden parental death from external causes. Journal of Affective Disorders. 2017;222:71-8.

24. Burrell LV, Mehlum L, Qin P. Sudden parental death from external causes and risk of suicide in the bereaved offspring: A national study. Journal of Psychiatric Research. 2018;96:49-56.

25. Cain AC, Fast I. THE LEGACY OF SUICIDE. OBSERVATIONS ON THE PATHOGENIC IMPACT OF SUICIDE UPON MARITAL PARTNERS. Psychiatry. 1966;29(4):406-11.

26. Caravaca Sanchez F, Ignatyev Y, Mundt AP. Associations between childhood abuse, mental health problems, and suicide risk among male prison populations in Spain. Criminal Behaviour & Mental Health. 2019;29(1):18-30.

27. Carbone JT, Holzer KJ, Vaughn MG. Child and Adolescent Suicidal Ideation and Suicide Attempts: Evidence from the Healthcare Cost and Utilization Project. Journal of Pediatrics. 2019;206:225-31.

28. Cassorla RM. Family characteristics of youngsters attempting suicide in Campinas, Brazil: a comparative study with normal and psychiatric youngsters. Acta psiquiatrica y psicologica de America latina. 1984;30(2):125-34.

29. Chapman AL, Specht MW, Cellucci T. Factors Associated with Suicide Attempts in Female Inmates: The Hegemony of Hopelessness. Suicide and Life-Threatening Behavior. 2005;35(5):558-69.

30. Cho J, Jung SH, Kim C, Suh M, Choi YJ, Sohn J, et al. Suicide loss, changes in medical care utilization, and hospitalization for cardiovascular disease and diabetes mellitus. European Heart Journal. 2016;37(9):764-70.

31. Chowdhury AN, Banerjee S, Brahma A, Das S, Sarker P, Biswas MK, et al. A prospective study of suicidal behaviour in Sundarban delta, West Bengal, India. National Medical Journal of India. 2010;23(4):201-5.

32. Clarke CS, Wrigley M. Suicide-related bereavement and psychiatric morbidity in the elderly. Irish Journal of Psychological Medicine. 2004;21(1):22-4.

33. Decke D, Lange E. [Family, educational and school conditions in suicide candidates]. Familien-, Erziehungs- und Schulbedingungen bei Suizidenten. 1978;72(18):901-4.

34. Dugas M, Mouren MC, Le Heuzey MF. Pathological mourning in children. Neuropsychiatrie de l'Enfance et de l'Adolescence. 1979;27(4-5):187-92.

35. Duggan C, Sham P, Minne C, Lee A, Murray R. Family history as a predictor of poor long-term outcome in depression. British Journal of Psychiatry. 1998;173(DEC.):527-30.

36. Dyregrov K, Dyregrov A. Siblings after Suicide -- "The Forgotten Bereaved". Suicide and Life-Threatening Behavior. 2005;35(6):714-24.

37. El Majzoub I, El Khuri C, Hajjar K, Bou Chebl R, Talih F, Makki M, et al. Characteristics of patients presenting post-suicide attempt to an Academic Medical Center Emergency Department in Lebanon. Annals of General Psychiatry. 2018;17:21.

38. Feigelman W, Jordan JR, Gorman BS. Personal Growth After a Suicide Loss: Cross-Sectional Findings Suggest Growth After Loss May be Associated with Better Mental Health among Survivors. Omega - Journal of Death and Dying. 2009;59(3):181-202.

39. Fekete S, Schmidtke A. Suicide-Modeling-Family. Szuicidium-Modellkovetes-Csalad: Transkulturalis osszehasonlito vizsgalat. 1995;10(2):131-45.

40. Ferrara P, Benincasa C, Galluccio C, Franceschini G, Mariotti P. Pediatric suicide is a preventable phenomenon: A single center experience. Minerva Psichiatrica. 2019;60(3):103-6.

41. Fowler KA, Crosby AE, Parks SE, Ivey AZ, Silverman PR. Epidemiological investigation of a youth suicide cluster: Delaware 2012. Delaware medical journal. 2013;85(1):15-9.

42. Garssen J, Deerenberg I, Mackenbach JP, Kerkhof A, Kunst AE. Familial Risk of Early Suicide: Variations by Age and Sex of Children and Parents. Suicide and Life-Threatening Behavior. 2011;41(6):585-613.

43. Gebremariam EH, Reta MM, Nasir Z, Amdie FZ. Prevalence and Associated Factors of Suicidal Ideation and Attempt among People Living with HIV/AIDS at Zewditu Memorial Hospital, Addis Ababa, Ethiopia: A Cross-Sectional Study. Psychiatry journal. 2017;2017:2301524.

44. Giupponi G, Innamorati M, Baldessarini RJ, De Leo D, de Giovannelli F, Pycha R, et al. Factors associated with suicide: Case-control study in South Tyrol. Comprehensive Psychiatry. 2018;80:150-4.

45. Goncalves Estella F, Acosta Lopez A, Ledesma Jimeno A. A clinical and epidemiologic study of the depression and the aggressiveness in a group of rural population. Estudio clinico y epidemiologico de las depresiones y la agresividad en un nucleo de poblacion rural. 1991;19(4):210-9.

46. Gonzalez-Castro TB, Tovilla-Zarate CA, Hernandez-Diaz Y, Juarez-Rojop IE, Leon-Garibay AG, Guzman-Priego CG, et al. Characteristics of Mexican children and adolescents who died by suicide: A study of psychological autopsies. Journal of Forensic and Legal Medicine. 2017;52:236-40.

47. Gregory RJ. Grief and loss among Eskimos attempting suicide in Western Alaska. American Journal of Psychiatry. 1994;151(12):1815-6.

48. Grendas LN, Rojas SM, Rodante DE, Puppo S, Vidjen P, Portela A, et al. Differential impact of child sexual abuse and family history of suicidal behavior in high-risk suicidal patients. Archives of Suicide Research. 2019:No Pagination Specified.

49. Grove O, Lynge I. Suicide and Attempted Suicide in Greenland: A Controlled Study in Nuuk (Godthaab). Acta Psychiatrica Scandinavica. 1979;60(4):375-91.

50. Hamaoka DA, Fullerton CS, Benedek DM, Gifford R, Nam T, Ursano RJ. Medical students' responses to an inpatient suicide: opportunities for education and support. Academic psychiatry : the journal of the American Association of Directors of Psychiatric Residency Training and the Association for Academic Psychiatry. 2007;31(5):350-3.

51. Hashim U. Title of the article: A study of suicidal attempts among the elderly in a rural area. Indian Journal of Psychiatry. 2019;61 (9 Supplement 3):S545.

52. Hazell P, Lewin T. Friends of adolescent suicide attempters and completers. Journal of the American Academy of Child and Adolescent Psychiatry. 1993;32(1):76.

53. Horwitz AG, Smith DL, Held P, Zalta AK. Characteristics of Veteran and Civilian Suicide Decedents: A Sex-Stratified Analysis. American Journal of Preventive Medicine. 2019;56(5):e163-e8.

54. Jeglic EL, Sharp IR, Chapman JE, Brown GK, Beck AT. History of family suicide behaviors and negative problem solving in multiple suicide attempters. Archives of Suicide Research. 2005;9(2):135-46.

55. Johnson BA, Brent DA, Bridge J, Connolly J. The familial aggregation of adolescent suicide attempts. Acta Psychiatrica Scandinavica. 1998;97(1):18-24.

56. Kawano K. [The mental health of persons bereaved by suicide]. Seishin shinkeigaku zasshi = Psychiatria et neurologia Japonica. 2011;113(1):87-93.

57. Kawashima D, Kawano K. Parental Grief After Offspring Suicide and Adaptation to the Loss in Japan. Omega. 2017:30222817710139.

58. Kawashima D, Kawano K. Parental Grief After Offspring Suicide and Adaptation to the Loss in Japan. Omega - Journal of Death & Dying. 2019;79(1):34-51.

59. Kelly B, Raphael B, Judd F, Perdices M, Kernutt G, Burnett P, et al. Suicidal ideation, suicide attempts, and HIV infection. Psychosomatics. 1998;39(5):405-15.

60. Khatwa SA, Abdou MH. Adult depression in Alexandria, Egypt, 1998. The Journal of the Egyptian Public Health Association. 1999;74(3-4):333-52.

61. Kolves K, Ross V, Hawgood J, Spence SH, De Leo D. The impact of a student's suicide: Teachers' perspectives. Journal of Affective Disorders. 2017;207:276-81.

62. Lazzarini TA, Goncalves CCM, Benites WM, Silva LFD, Tsuha DH, Ko AI, et al. Suicide in Brazilian indigenous communities: clustering of cases in children and adolescents by household. Revista de Saude Publica. 2018;52:56.

63. Li J, Vestergaard M, Cnattingius S, Gissler M, Bech BH, Obel C, et al. Mortality after Parental Death in Childhood: A Nationwide Cohort Study from Three Nordic Countries. PLoS Medicine. 2014;11(7):e1001679.

64. Linker J, Gillespie NA, Maes H, Eaves L, Silberg JL. Suicidal Ideation, Depression, and Conduct Disorder in a Sample of Adolescent and Young Adult Twins. Suicide and Life-Threatening Behavior. 2012;42(4):426-36.

65. Liu B-P, Qin P, Jia C-X. Behavior Characteristics and Risk Factors for Suicide Among the Elderly in Rural China. The Journal of nervous and mental disease. 2018;206(3):195-201.

66. Liu BP, Wang XT, Zhang J, Chu J, Pan YF, Yu PP, et al. The relationship between mental stimulation level of life events and suicide attempt of rural residents in Shandong Province. [Chinese]. Zhonghua yu fang yi xue za zhi [Chinese journal of preventive medicine]. 2019;53(9):896-9.

67. Liu Y, Zhang J, Sun L, Zhao S. The age-specific characteristics of medically serious suicide attempters aged 15-45 years in rural China. Psychiatry Research. 2018;261:178-85.

68. Lizardi D, Sher L, Sullivan GM, Stanley B, Burke A, Oquendo MA. Association between familial suicidal behavior and frequency of attempts among depressed suicide attempters. Acta Psychiatrica Scandinavica. 2009;119(5):406-10.

69. Lopez-Castroman J, Jaussent I, Beziat S, Genty C, Olie E, de Leon-Martinez V, et al. Suicidal phenotypes associated with family history of suicidal behavior and early traumatic experiences. Journal of Affective Disorders. 2012;142(1-3):193-9.

70. Matthieu MMP, Gardiner G, Ziegemeier E, Buxton M, Han L, Cross W. Personal and Professional Knowledge of and Experience With Suicide and Suicide Prevention Among Stakeholders in Clinical and Community Practice. Social work in mental health. 2014;12(5-6):443-56.

71. McCluskey CK, Allareddy V, Rampa S, Allareddy V, Rotta AT. Characterization of Suicide and Deliberate Self-Harm Among Children in the United States. Clinical Pediatrics. 2019;58(1):66-72.

72. McGirr A, Alda M, Seguin M, Cabot S, Lesage A, Turecki G. Familial aggregation of suicide explained by cluster B traits: A three-group family study of suicide controlling for major depressive disorder. American Journal of Psychiatry. 2009;166(10):1124-34.

73. Melhem NM, Walker M, Moritz G, Brent DA. Antecedents and sequelae of sudden parental death in offspring and surviving caregivers. Archives of Pediatrics and Adolescent Medicine. 2008;162(5):403-10.

74. Mirahmadizadeh A, Rezaei F, Mokhtari AM, Gholamzadeh S, Baseri A. Epidemiology of suicide attempts and deaths: a population-based study in Fars, Iran (2011-16). J Public Health (Oxf). 2019;22:22.

75. Mitchell AM, Kim Y, Prigerson HG, Mortimer MK. Complicated grief and suicidal ideation in adult survivors of suicide. Suicide and Life-Threatening Behavior. 2005;35(5):498-506.

76. Mitchell AM, Sakraida TJ, Kim Y, Bullian L, Chiappetta L. Depression, Anxiety and Quality of Life in Suicide Survivors: A Comparison of Close and Distant Relationships. Archives of Psychiatric Nursing. 2009;23(1):2-10.

77. Murphy SA, Tapper VJ, Johnson LC, Lohan J. Suicide ideation among parents bereaved by the violent deaths of their children. Issues in mental health nursing. 2003;24(1):5-25.

78. Nam I. Suicide Bereavement and Complicated Grief: Experiential Avoidance as a Mediating Mechanism. Journal of Loss & Trauma. 2016;21(4):325-34.

79. Niederkrotenthaler T, Floderus B, Alexanderson K, Rasmussen F, Mittendorfer-Rutz E. Exposure to parental mortality and markers of morbidity, and the risks of attempted and completed suicide in offspring: An analysis of sensitive life periods. Journal of Epidemiology and Community Health. 2012;66(3):233-9.

80. Nierenberg AA, Alpert JE, Gaynes BN, Warden D, Wisniewski SR, Biggs MM, et al. Family history of completed suicide and characteristics of major depressive disorder: A STARD (sequenced treatment alternatives to relieve depression) study. Journal of Affective Disorders. 2008;108(1-2):129-34.

81. Nyberg T, Myrberg IH, Omerov P, Steineck G, Nyberg U. Depression among parents two to six years following the loss of a child by suicide: A novel prediction model. PLoS ONE. 2016;11(10):e0164091.

82. Oyen N, Boyd HA, Poulsen G, Wohlfahrt J, Melbye M. The clustering of premature deaths in families. Epidemiology. 2009;20(5):757-65.

83. Pfeffer CR, Karus D, Siegel K, Jiang H. Child survivors of parental death from cancer or suicide: depressive and behavioral outcomes. Psycho-Oncology. 2000;9(1):1-10.

84. Pitman AL, Osborn DPJ, Rantell K, King MB. Bereavement by suicide as a risk factor for suicide attempt: A cross-sectional national UK-wide study of 3432 young bereaved adults. BMJ Open. 2016;6(1):e009948.

85. Prigerson HG, Bridge J, Maciejewski PK, Beery LC, Rosenheck RA, Jacobs SC, et al. Influence of traumatic grief on suicidal ideation among young adults. American Journal of Psychiatry. 1999;156(12):1994-5.

86. Pulmanis T, Trapencieris M, Taube M. Adolescent self-reported lifetime suicide attempts in latvia: Family related factors. Atencion Primaria. 2013;45(SUPPL. 2):123.

87. Raj MAJ, Kumaraiah V, Bhide AV. Social and clinical factors related to deliberate self harm. NIMHANS Journal. 2000;18(1-2):3-18.

88. Rajendran K. Psycho-socio-demographic and clinical profile of single and multiple suicide attempters in a tertiary care centre-a comparative study. Indian Journal of Psychiatry. 2019;61 (9 Supplement 3):S506.

89. Rende R, Warner V, Wickramarante P, Weissman MM. Sibling aggregation for psychiatric disorders in offspring at high and low risk for depression: 10-year followup. Psychological Medicine. 1999;29(6):1291-8.

90. Saarinen PI, Hintikka J, Vnamaki H, Lehtonen J, Lonnqvist J. Is it possible to adapt to the the suicide of a close individual? Results of a 10-year prospective follow-up study. International Journal of Social Psychiatry. 2000;46(3):182-90.

91. Schachter S. Adolescent Experiences with the Death of a Peer. Omega. 1992;24(1):1-11.

92. Shah M, Yousafzai AW, Khan MZ, Khan MM. Clinical and demographic characteristics of patients presenting with deliberate self harm in a tertiary care hospital-Pakistan. Journal of Medical Sciences (Peshawar). 2019;27(2):71-4.

93. Spillane A, Matvienko-Sikar K, Larkin C, Corcoran P, Arensman E. What are the physical and psychological health effects of suicide bereavement on family members? An observational and interview mixed-methods study in Ireland. BMJ open. 2018;8(1):e019472.

94. Tang CP, Hung SF, Lee CC, Ho TP, Leung PW. 15-year computer-record study of adolescents exposed to peer suicide. Hong Kong Medical Journal. 2019;25 Suppl 3(1):11-2.

95. Tanner AK, Hasking P, Martin G. Suicidality among adolescents engaging in nonsuicidal self-injury (NSSI) and firesetting: The role of psychosocial characteristics and reasons for living. Child and Adolescent Psychiatry and Mental Health. 2015;9(1):33.

96. Thomson P, Jaque SV. Posttraumatic stress disorder and psychopathology in dancers. Medical Problems of Performing Artists. 2015;30(3):157-62.

97. Tong Y, Phillips MR, Duberstein P, Zhan W. Suicidal Behavior in Relatives or Associates Moderates the Strength of Common Risk Factors for Suicide. Suicide & life-threatening behavior. 2015;45(4):505-17.

98. Venkoba Rao A, Mahendran N, Gopalakrishnan C, Reddy TK, Prabhakar ER, Swaminathnan R, et al. One hundred female burns cases : a study in suicidology. Indian journal of psychiatry. 1989;31(1):43-50.

99. Voltas N, Hernandez-Martinez C, Arija V, Canals J. Suicidality in a Community Sample of Early Adolescents: A Three-Phase Follow-Up Study. Archives of Suicide Research. 2019:1-19.

100. Waern M. Suicides among family members of elderly suicide victims: An exploratory study. Suicide and Life-Threatening Behavior. 2005;35(3):356-64.

101. Wedig MM, Silverman MH, Frankenburg FR, Reich DB, Fitzmaurice G, Zanarini MC. Predictors of suicide attempts in patients with borderline personality disorder over 16 years of prospective follow-up. Psychological Medicine. 2012;42(11):2395-404.

102. Wenz FV. Family constellation factors, depression, and parent suicide potential. The American journal of orthopsychiatry. 1979;49(1):164-7.

103. Winokur G, Morrison J, Clancy J, Crowe R. The Iowa 500: familial and clinical findings favor two kinds of depressive illness. Comprehensive Psychiatry. 1973;14(2):99-106.

104. Wittkowski J, Scheuchenpflug R. Grief depending on kinship relationship with the deceased and on mode of death. Trauern in Abhangigkeit vom Verwandtschaftsverhaltnis zum Verstorbenen und der Todesart. 2016;24(3):107-18.

105. Wojtkowiak J, Wild V, Egger J. Grief experiences and expectance of suicide. Suicide & life-threatening behavior. 2012;42(1):56-66.

106. Yousaf F, Hawthorne M, Sedgwick P. Impact of patient suicide on psychiatric trainees. Psychiatric Bulletin. 2002;26(2):53-5.

107. Zimmermann P, Hollmer H, Guhn A, Strohle A. Predictors of suicidality in German soldiers. Nervenarzt. 2012;83(3):359-65.

## **Study population not eligible**

1. Abel KM, Heuvelman HP, Jörgensen L, Magnusson C, Wicks S, Susser E, et al. Severe bereavement stress during the prenatal and childhood periods and risk of psychosis in later Life: population based cohort study. British Medical Journal. 2014;348(7942):13.

2. Birmaher B, Brent DA, Brodsky B, Ellis SP, Firinciogullari S, Greenhill L, et al. Peripubertal suicide attempts in offspring of suicide attempters with siblings concordant for suicidal behavior. American Journal of Psychiatry. 2003;160(8):1486-93.

3. Borges G, Nock MK, Haro Abad JM, Hwang I, Sampson NA, Alonso J, et al. Twelve-month prevalence of and risk factors for suicide attempts in the World Health Organization World Mental Health Surveys. The Journal of Clinical Psychiatry. 2010;71(12):1617-28.

4. Byrne M, Agerbo E, Mortensen PB. Family history of psychiatric disorders and age at first contact in schizophrenia: An epidemiological study. British Journal of Psychiatry. 2002;181(SUPPL. 43):s19-s25.

5. Choudhary P, Gupta B, Nischal A, Tripathi A, Agarwal M. A study suicidal of intent and temporal variation in subjects with suicide attempt. Indian Journal of Psychiatry. 2019;61 (9 Supplement 3):S546.

6. Fazel S, Wolf A, Palm C, Lichtenstein P. Violent crime, suicide, and premature mortality in patients with schizophrenia and related disorders: A 38-year total population study in Sweden. The Lancet Psychiatry. 2014;1(1):44-54.

7. Gunter TD, Chibnall JT, Antoniak SK, McCormick B, Black DW. Relative Contributions of Gender and Traumatic Life Experience to the Prediction of Mental Disorders in a Sample of Incarcerated Offenders. Behavioral Sciences & the Law. 2012;30(5):615-30.

8. Hettiarachchi LV, Kinner SA, Tibble H, Borschmann R. Self-harm among young people detained in the youth justice system in Sri Lanka. International Journal of Environmental Research and Public Health. 2018;15(2):209.

9. Ljung T, Chen Q, Lichtenstein P, Larsson H. Common etiological factors of attention-deficit/hyperactivity disorder and suicidal behavior: A population-based study in Sweden. JAMA Psychiatry. 2014;71(8):958-64.

10. Marzano L, Hawton K, Rivlin A, Fazel S. Psychosocial influences on prisoner suicide: A case-control study of near-lethal self-harm in women prisoners. Social Science & Medicine. 2011;72(6):874-83.

11. Muller M, Vandeleur C, Rodgers S, Rossler W, Castelao E, Preisig M, et al. Childhood adversities as specific contributors to the co-occurrence of posttraumatic stress and alcohol use disorders. Psychiatry Research. 2015;228(3):251-6.

12. Pawlak JM, Miechowicz I, Dmitrzak-Weglarz M, Szczepankiewicz A, Zaremba D, Kapelski P, et al. Are suicide risk factors gender specific? Psychiatria Polska. 2018;52(1):21-32.

13. Roley-Roberts ME, Hill RM, Layne CM, Goldenthal H, Kaplow JB. Cause of Caregiver Death and Surviving Caregiver Coping Style Predict Thwarted Belongingness in Bereaved Youth. Archives of suicide research : official journal of the International Academy for Suicide Research. 2018:1-32.

14. Roy A, Janal M. Family history of suicide, female sex, and childhood trauma: Separate or interacting risk factors for attempts at suicide? Acta Psychiatrica Scandinavica. 2005;112(5):367-71.

15. Salokangas RKR, Patterson P, Hietala J, Heinimaa M, From T, Ilonen T, et al. Childhood adversity predicts persistence of suicidal thoughts differently in females and males at clinical high-risk patients of psychosis. Results of the EPOS project. Early intervention in psychiatry. 2018:23.

16. Segal NL. Suicidal behaviors in surviving monozygotic and dizygotic co-twins: is the nature of the co-twin's cause of death a factor? Suicide Life Threat Behav. 2009;39(6):569-75.

17. Sidorchuk A, Kuja-Halkola R, Runeson B, Lichtenstein P, Larsson H, Ruck C, et al. Genetic and environmental sources of familial coaggregation of obsessive-compulsive disorder and suicidal behavior: A population-based birth cohort and family study. Molecular Psychiatry. 2019:No Pagination Specified.

18. Smith MT, Edwards RR, Robinson RC, Dworkin RH. Suicidal ideation, plans, and attempts in chronic pain patients: Factors associated with increased risk. Pain. 2004;111(1-2):201-8.

19. Tremeau F, Staner L, Duval F, Bailey P, Crocq MA, Correa H, et al. Suicidal behavior in schizophrenia and family history of suicide. Dialogues in clinical neuroscience. 2001;3(2):120-1.

20. Tremeau F, Staner L, Duval F, Correa H, Crocq MA, Darreye A, et al. Suicide attempts and family history of suicide in three psychiatric populations. Suicide and Life-Threatening Behavior. 2005;35(6):702-13.

## Reviews, abstracts, dissertations

1. Abramowitz ES. Risk factors associated with adolescent suicide attempting. Dissertation Abstracts International. 1992;53(4-B):2048.

2. Ahern J, Karasek D, Bruckner TA. Racial/ethnic differences in the relation between severe childhood adverse events and adolescent mental health disorders. American Journal of Epidemiology. 2013;177(SUPPL. 11):S28.

3. Aupperle DR. Sibling death in adolescence: The relationship of coping responses to adjustment, stressor type, and age. Dissertation Abstracts International: Section B: The Sciences and Engineering. 1995;56(2-B):1098.

4. Azure JA. Depressed native Americans and suicidal ideation contagion. Dissertation Abstracts International: Section B: The Sciences and Engineering. 2013;73(10-B(E)):No-Specified.

5. Ballard E, Cui L, Machado-Vieira R, Zarate C, Merikangas K. Anxiety disorders underlie the familial transmission of suicide attempts. Neuropsychopharmacology. 2015;40(SUPPL. 1):S274-S5.

6. Bolton JM, Spiwak R, Sareen J. Consequences of sibling death: Problematic, potentially predictable, and poorly managed. JAMA Pediatrics. 2017;171(6):519-20.

7. Carballo JJ, Llorente C, Kehrmann L, Flamarique I, Zuddas A, Purper-Ouakil D, et al. Psychosocial risk factors for suicidality in children and adolescents. European Child & Adolescent Psychiatry. 2019:25.

8. Carmassi C, Pergentini I, Calderani E, Manni C, Menichini M, Gemignani S, et al. Validation of the italian version of the inventory of complicated grief (ICG). European Neuropsychopharmacology. 2013;23(SUPPL. 2):S627-S8.

9. Chen R, An J, Ou J. Suicidal behaviour among children and adolescents in China. The Lancet Child & Adolescent Health. 2018;2(8):551-3.

10. Cohen JA, Mannarino AP. Helping Children With Traumatic Reactions to Parental Suicide. Journal of the American Academy of Child & Adolescent Psychiatry. 2018;57(8):619.

11. Cole JA. Parental bereavement: An investigation of the short-term and long-term effects. Dissertation Abstracts International: Section B: The Sciences and Engineering. 2000;61(6-B):3272.

12. Dickens N. Prevalence of Complicated Grief and Posttraumatic Stress Disorder in Children and Adolescents Following Sibling Death. Family Journal. 2014;22(1):119.

13. Dispenza C. Reintegration and renewed meaning and purpose in life of bereaved parents after the suicidal death of a child. Dissertation Abstracts International. 1993;53(9-B):5002.

14. Evans EA. Childhood adversity and the presence and persistence of substance use disorders over the life course among a nationally representative sample of adult women. Dissertation Abstracts International: Section B: The Sciences and Engineering. 2016;77(5-B(E)):No-Specified.

15. Farber ML. Factors Determining the Incidence of Suicide within Families. Suicide and Life-Threatening Behavior. 1977;7(1):3-6.

16. Garcia-Williams AG. Suicide behavior in college students and peers' response. Dissertation Abstracts International: Section B: The Sciences and Engineering. 2016;76(12-B(E)):No-Specified.

17. Garrett JE. Multiple losses in older adults. Journal of Gerontological Nursing. 1987;13(8):8-12.

18. Hagopian LP, Frank-Crawford MA. Classification of self-injurious behaviour across the continuum of relative environmental-biological influence. Journal of Intellectual Disability Research. 2018;62(12):1108-13.

19. Hannah SD, Jr. Family characteristics that influence and differentiate adolescent non-single, and multiple suicide attempts. Dissertation Abstracts International: Section B: The Sciences and Engineering. 2004;65(5-B):2608.

20. Hanson M, Tiberius R, Hodges B, Mackay S, McNaughton N, Dickens S, et al. Implications of suicide contagion for the selection of adolescent standardized patients. Academic Medicine. 2002;77(10 SUPPL.):S100-S2.

21. Hill O. Some psychiatric non-sequelae of childhood bereavement. The British journal of psychiatry : the journal of mental science. 1970;116(535):679-80.

22. Hill OW. Child bereavement and adult psychiatric disturbance. Journal of psychosomatic research. 1972;16(5):357-60.

23. Howard EL. Incidence and impact of client suicide on health service providers in psychology. Dissertation Abstracts International: Section B: The Sciences and Engineering. 2001;61(9-B):4986.

24. Howe-Martin LS. Adolescent self-mutilating behaviors: Experiential avoidance coupled with imitation? Dissertation Abstracts International: Section B: The Sciences and Engineering. 2009;70(4-B):2574.

25. Kelly TM. A comparison of suicide completers with psychiatrically disordered and non-disordered subjects via the psychological autopsy method. Dissertation Abstracts International: Section B: The Sciences and Engineering. 1997;57(7-B):4712.

26. Kersting A. Are suicide dependants liable to an increased risk of traumatic mourning? Psychiatrische Praxis. 2002;29(1):48-9.

27. Kestenbaum JY. Suicidal versus non-suicidal children: Psychological, cognitive, psychiatric and family history differences. Dissertation Abstracts International. 1993;53(11-B):5980.

28. Li J, Vestergaard M, Cnattingus S, Gissler M, Bech BH, Obel C, et al. Mortality after parental death in childhood: A nationwide cohort study from 3 Nordic countries. European Journal of Epidemiology. 2013;28(1 SUPPL. 1):S240.

29. Lieb R, Bronisch T, Hofler M, Schreier A, Wittchen HU. Maternal suicidality and risk of suicidality in offspring: Findings from a community study. American Journal of Psychiatry. 2005;162(9):1665-71.

30. Liebscher BJ. Parental death and the impact on grieving children: A comparison of homicide or suicide and natural or accidental deaths. Dissertation Abstracts International: Section B: The Sciences and Engineering. 2001;62(2-B):1089.

31. Liu H, Liu S, Tjung J, Huang Y. Recent exposure to others' suicidal thoughts increases the risk of deliberate self-harm in adolescents. Neuropsychiatrie de l'Enfance et de l'Adolescence. 2012;60(5 SUPPL. 1):S151.

32. Maria Cecilia deSouza M, Bastos Figueiredo AE, Raimunda Matilde doNascimento M. Estudo das publicações científicas (2002-2017) sobre ideação suicida, tentativas de suicídio e autonegligência de idosos internados em Instituições de Longa Permanência. Ciência & Saúde Coletiva. 2019;24(4).

33. Marmer MA. Parental loss of a child: A comparison of sudden illness, accident, or the suicidal death of a child. Dissertation Abstracts International: Section B: The Sciences and Engineering. 1995;56(3-B):1704.

34. Martinez MS. Correlates of suicide-related behaviors among children ages six to twelve. Dissertation Abstracts International: Section B: The Sciences and Engineering. 2015;76(5-B(E)):No-Specified.

35. McGee PD. Movements in bereavement: The bereavement experience of parents who lose a grown child through a suicide or non-suicide sudden death. Dissertation Abstracts International Section A: Humanities and Social Sciences. 1994;54(9-A):3331.

36. McLoughlin DP. Suicide among Canadian First Nations males. Dissertation Abstracts International: Section B: The Sciences and Engineering. 2007;68(1-B):629.

37. McMahon E, Corcoran P, Keeley H, Perry I, Arensman E. Adolescents exposed to suicidal behaviour of others: Prevalence of self-harm and associated psychological, lifestyle and life event factors. European Child and Adolescent Psychiatry. 2013;22(2 SUPPL. 1):S290.

38. Melhem NM. Traumatic grief among adolescents exposed to their peer's suicide. Dissertation Abstracts International: Section B: The Sciences and Engineering. 2002;63(1-B):172.

39. Menard C. Clustering of child abuse, parental psychopathology, and other family-level stressors and children's psychosocial outcomes. Dissertation Abstracts International: Section B: The Sciences and Engineering. 2004;64(10-B):4891.

40. Minayo MCS, Figueiredo AEB, Mangas R. Study of scientific publications (2002-2017) on suicidal ideation, suicide attempts and self-neglect of elderly people hospitalized in Long-Term Care Establishments. Ciencia & Saude Coletiva. 2019;24(4):1393-404.

41. Moore MM. Posttraumatic growth among parent survivors of suicide. Dissertation Abstracts International: Section B: The Sciences and Engineering. 2013;74(3-B(E)):No-Specified.

42. Murphy SA. Parent Bereavement stress and preventive intervention following the violent deaths of adolescent or young adult children. Death Studies. 1996;20(5):441-52.

43. Paul EK. The development of suicidal ideation and self-harm in childhood and adolescence. Dissertation Abstracts International: Section B: The Sciences and Engineering. 2018;79(2-B(E)):No-Specified.

44. Pedersen NL, Fiske A. Genetic influences on suicide and nonfatal suicidal behavior: Twin study findings. European Psychiatry. 2010;25(5):264-7.

45. Pynoos RS, Nader K. Children's exposure to violence and traumatic death. Psychiatric Annals. 1990;20(6):334-44.

46. Razzino BE. The impact of violence on children: Specific effects and mediating processes. Dissertation Abstracts International: Section B: The Sciences and Engineering. 2000;60(8-B):4247.

47. Roach A. A concept analysis of adolescent friendship. Nursing Forum. 2019:25.

48. Roberts WD, III. Impact of client suicide and guilt on the mental health professional. Dissertation Abstracts International: Section B: The Sciences and Engineering. 2014;74(12-B(E)):No-Specified.

49. Rodriguez EP. Contributing factors of suicidal ideation in Mexican Americans in the central valley of California. Dissertation Abstracts International: Section B: The Sciences and Engineering. 2008;69(3-B):1970.

50. Schafrik-Arsenault J. Maternal health and psychopathology: Impact on child behavior and development. Dissertation Abstracts International: Section B: The Sciences and Engineering. 1997;57(9-B):5950.

51. Sheline KT. Posttraumatic growth and suicide risk in college students according to trauma type: A failure to replicate. Dissertation Abstracts International: Section B: The Sciences and Engineering. 2016;77(1-B(E)):No-Specified.

52. Siegel EJ. Conjugal bereavement in older adults: World assumptions, depression, and suicidal ideation. Dissertation Abstracts International: Section B: The Sciences and Engineering. 2002;62(11-B):5393.

53. Silva NH. The variables associated with drug abuse: An archival study of inpatient adolescents. Dissertation Abstracts International: Section B: The Sciences and Engineering. 1997;58(5-B):2700.

54. Smith PC. Bereavement outcome following suicidal, homicidal, accidental, and natural deaths. Dissertation Abstracts International. 1990;51(4-B):2074-5.

55. Sood AB, Razdan A, Weller EB, Weller RA. Children's reactions to parental and sibling death. Current Psychiatry Reports. 2006;8(2):115-20.

56. Sotero MM. The effects of adverse childhood experiences on subsequent injury in young adulthood: Findings from the National Longitudinal Study of Adolescent and Adult Health. Dissertation Abstracts International: Section B: The Sciences and Engineering. 2016;76(12-B(E)):No-Specified.

57. Stoelb M, Chiriboga J. A process model for assessing adolescent risk for suicide. Journal of Adolescence. 1998;21(4):359-70.

58. Torzsa P, Eory A, Gonda X, Kalabay L, Rihmer Z. The importance of family suicide history in the screening of depression in primary care. European Journal of General Practice. 2012;18(3):175-6.

59. Trolley BC. Suicide parents and sudden death parents: The grief process, the nature of help, the role of the professional, and methodological issues. Dissertation Abstracts International. 1986;46(7-B):2449-50.

60. Vessier-Batchen M. Life after death: A comparison of coping and symptoms of complicated grief in survivors of homicide and suicide decedents. Dissertation Abstracts International: Section B: The Sciences and Engineering. 2007;68(6-B):3697.

61. Voracek M. Suicide risk after spousal suicide or psychiatric admission: Effects of assortative mating on heritable traits compared with environmental explanations. Journal of Epidemiology and Community Health. 2005;59(5):347-8.

62. Vrabie MI, Marinescu V, Enache D, Vuta R. Assessing the relationship between history of suicide attempts and cognitive dysfunction in bipolar affective disorder. International Journal of Neuropsychopharmacology. 2012;15(SUPPL. 1):88-9.

63. Vrabtchev SV. Grieving the death of a child: supporting health professionals who care for grieving patients may benefit all. BMJ (Clinical research ed). 2006;332(7544):794-5.

64. Watkins RL. Exposure to peer suicide in college students. Dissertation Abstracts International: Section B: The Sciences and Engineering. 2005;65(9-B):4856.

65. Wright TR. The impact of suicide during childhood on the mourning process and psychosocial functioning of child and sibling survivors. Dissertation Abstracts International: Section B: The Sciences and Engineering. 1999;59(10-B):5591.

66. Wyss J. Perceived open communication following parental suicide. Dissertation Abstracts International: Section B: The Sciences and Engineering. 2003;64(2-B):977.

67. Yates TM. A longitudinal study of self-injurious behavior in a community sample. Dissertation Abstracts International: Section B: The Sciences and Engineering. 2006;66(8-B):4518.

68. Zolot J. HEALTH EFFECTS FOLLOWING THE SUICIDE OF A SPOUSE. AJN American Journal of Nursing. 2017;117(7):64-.

69. Zuckerman S. What predicts suicide among psychologists? An examination of vulnerability and protective factors. Dissertation Abstracts International: Section B: The Sciences and Engineering. 2018;79(4-B(E)):No-Specified.

## Combined exposure to suicidal behaviour with other mortality/morbidity exposures

1. Berg L, Rostila M, Hjern A. Parental death during childhood and depression in young adults - a national cohort study. Journal of child psychology and psychiatry, and allied disciplines. 2016;57(9):1092-8.

2. Bjorkenstam E, Hjern A, Mittendorfer-Rutz E, Vinnerljung B, Hallqvist J, Ljung R. Multi-Exposure and Clustering of Adverse Childhood Experiences, Socioeconomic Differences and Psychotropic Medication in Young Adults. PLoS ONE. 2013;8(1):e53551.

3. Boelen PA, Huntjens RJC. Intrusive images in grief: An exploratory study. Clinical Psychology and Psychotherapy. 2008;15(4):217-26.

4. Boelen PA, Prigerson HG. The influence of symptoms of prolonged grief disorder, depression, and anxiety on quality of life among bereaved adults: a prospective study. European archives of psychiatry and clinical neuroscience. 2007;257(8):444-52.

5. Bolton JM, Au W, Chateau D, Walld R, Leslie WD, Enns J, et al. Bereavement after sibling death: A population-based longitudinal case-control study. World Psychiatry. 2016;15(1):59-66.

6. Brenn T, Ytterstad E. Increased risk of death immediately after losing a spouse: Cause-specific mortality following widowhood in Norway. Preventive Medicine. 2016;89:251-6.

7. Carballo JJ, Harkavy-Friedman J, Burke AK, Sher L, Enriquebaca G, Sullivan GM, et al. Family history of suicidal behavior and early traumatic experiences: Additive effect on suicidality and course of bipolar illness? Journal of Affective Disorders. 2008;109(1-2):57-63.

8. Cleiren M, Diekstra RF, Kerkhof AJ, van der Wal J. Mode of death and kinship in bereavement: focusing on "who" rather than "how". Crisis. 1994;15(1):22-36.

9. Davidson LE, Rosenberg ML, Mercy JA, Franklin J, Simmons JT. An epidemiologic study of risk factors in two teenage suicide clusters. Journal of the American Medical Association. 1989;262(19):2687-92.

10. Feigelman W, Gorman BS, Jordan JR. Stigmatization and Suicide Bereavement. Death Studies. 2009;33(7):591-608.

11. Gray LB, Weller RA, Fristad M, Weller EB. Depression in children and adolescents two months after the death of a parent. Journal of Affective Disorders. 2011;135(1-3):277-83.

12. Guldin MB, Ina Siegismund Kjaersgaard M, Fenger-Gron M, Thorlund Parner E, Li J, Prior A, et al. Risk of suicide, deliberate self-harm and psychiatric illness after the loss of a close relative: A nationwide cohort study. World Psychiatry. 2017;16(2):193-9.

13. Gutierrez PM. Suicidality in parentally bereaved adolescents. Death Studies. 1999;23(4):359-70.

14. Hamdan S, Mazariegos D, Melhem NM, Porta G, Payne MW, Brent DA. Effect of parental bereavement on health risk behaviors in youth: A 3-year follow-up. Archives of Pediatrics and Adolescent Medicine. 2012;166(3):216-23.

15. Hamdan S, Melhem NM, Porta G, Song MS, Brent DA. Alcohol and substance abuse in parentally bereaved youth. Journal of Clinical Psychiatry. 2013;74(8):828-33.

16. Hardison HG, Neimeyer RA, Lichstein KL. Insomnia and complicated grief symptoms in bereaved college students. Behavioral sleep medicine. 2005;3(2):99-111.

17. Hjern A, Bremberg S. Social aetiology of violent deaths in Swedish children and youth. Journal of epidemiology and community health. 2002;56(9):688-92.

18. Hopmeyer E, Werk A. A Comparative Study of Four Family Bereavement Groups. Groupwork. 1993;6(2):107-21.

19. Houtepen LC, Heron J, Suderman MJ, Tilling K, Howe LD. Adverse childhood experiences in the children of the Avon Longitudinal Study of Parents and Children (ALSPAC). Wellcome Open Research. 2018;3:106.

20. Jeon HJ, Hong JP, Fava M, Mischoulon D, Nyer M, Inamori A, et al. Childhood Parental Death and Lifetime Suicide Attempt of the Opposite-Gender Offspring in a Nationwide Community Sample of Korea. Suicide and Life-Threatening Behavior. 2013;43(6):598-610.

21. Johnson LC, Murphy SA, Dimond M. Reliability, construct validity, and subscale norms of the Brief Symptom Inventory when administered to bereaved parents. Journal of nursing measurement. 1996;4(2):117-27.

22. Laursen TM, Munk-Olsen T, Nordentoft M, Mortensen PB. A comparison of selected risk factors for unipolar depressive disorder, bipolar affective disorder, schizoaffective disorder, and schizophrenia from a Danish population-based cohort. Journal of Clinical Psychiatry. 2007;68(11):1673-81.

23. Lohan JA, Murphy SA. MENTAL DISTRESS AND FAMILY FUNCTIONING AMONG MARRIED PARENTS BEREAVED BY A CHILD'S SUDDEN DEATH. Omega. 2005;52(4):295-305.

24. Muller-Fahlbusch H. Endogenous depressive phases following the death of a relative. Provocation of endogenous depressive phases. Der Nervenarzt. 1971;42(8):426-31.

25. Muniz-Cohen M, Melhem NM, Brent DA. Health risk behaviors in parentally bereaved youth. Archives of Pediatrics and Adolescent Medicine. 2010;164(7):621-4.

26. Murphy SA, Braun T, Tillery L, Cain KC, Johnson LC, Beaton RD. PTSD among Bereaved Parents following the Violent Deaths of Their 12- to 28-Year-Old Children: A Longitudinal Prospective Analysis. Journal of Traumatic Stress. 1999;12(2):273-91.

27. Murphy SA, Chung IJ, Johnson LC. Patterns of mental distress following the violent death of a child and predictors of change over time. Research in nursing & health. 2002;25(6):425-37.

28. Murphy SA, Das Gupta A, Cain KC, Johnson LC, Lohan J, Wu L, et al. Changes in parents' mental distress after the violent death of an adolescent or young adult child: A longitudinal prospective analysis. Death Studies. 1999;23(2):129-59.

29. Murphy SA, Johnson LC, Chung IJ, Beaton RD. The prevalence of PTSD following the violent death of a child and predictors of change 5 years later. Journal of Traumatic Stress. 2003;16(1):17-25.

30. Murphy SA, Johnson LC, Lohan J. The aftermath of the violent death of a child: An integration of the assessments of parents' mental distress and PTSD during the first 5 years of bereavement. Journal of Loss and Trauma. 2002;7(3):203-22.

31. Murphy SA, Johnson LC, Lohan J, Tapper VJ. Bereaved parents' use of individual, family, and community resources 4 to 60 months after a child's violent death. Family & community health. 2002;25(1):71-82.

32. Murphy SA, Johnson LC, Weber NA. Coping strategies following a child's violent death: How parents differ in their responses. Omega. 2002;45(2):99-118.

33. O'Neill S, McLafferty M, Ennis E, Lapsley C, Bjourson T, Armour C, et al. Socio-demographic, mental health and childhood adversity risk factors for self-harm and suicidal behaviour in College students in Northern Ireland. Journal of Affective Disorders. 2018;239:58-65.

34. Payton JB, Krocker-Tuskan M. Children's reactions to loss of parent through violence. Journal of the American Academy of Child & Adolescent Psychiatry. 1988;27(5):563-6.

35. Peltzer K. Posttraumatic stress symptoms in a population of rural children in South Africa. Psychological reports. 1999;85(2):646-50.

36. Pham S, Porta G, Biernesser C, Walker Payne M, Iyengar S, Melhem N, et al. The Burden of Bereavement: Early-Onset Depression and Impairment in Youths Bereaved by Sudden Parental Death in a 7-Year Prospective Study. The American journal of psychiatry. 2018:appiajp201817070792.

37. Pinto VCP, Alves JFC, Maia AC. Adversity in childhood predicts depressive symptoms and suicide attempts in adult Portuguese women. Adversidade na infancia prediz sintomas depressivos e tentativas de suicidio em mulheres adultas portuguesas. 2015;32(4):617-25.

38. Pitkänen J, Remes H, Aaltonen M, Martikainen P. Experience of maternal and paternal adversities in childhood as determinants of self-harm in adolescence and young adulthood. Journal of Epidemiology and Community Health. 2019;73(11):1040.

39. Prigerson HG, Bierhals AJ, Kasl SV, Reynolds ICF, Shear MK, Day N, et al. Traumatic grief as a risk factor for mental and physical morbidity. American Journal of Psychiatry. 1997;154(5):616-23.

40. Raleva M. Early Life Stress: a Key Link between Childhood Adversity and Risk of Attempting Suicide. Psychiatria Danubina. 2018;30(Suppl 6):341-7.

41. Reed MD. Sudden death and bereavement outcomes: the impact of resources on grief symptomatology and detachment. Suicide & life-threatening behavior. 1993;23(3):204-20.

42. Roos LE, Afifi TO, Martin CG, Pietrzak RH, Tsai J, Sareen J. Linking typologies of childhood adversity to adult incarceration: Findings from a nationally representative sample. American Journal of Orthopsychiatry. 2016;86(5):584.

43. Rostila M, Berg L, Arat A, Vinnerljung B, Hjern A. Parental death in childhood and self-inflicted injuries in young adults-a national cohort study from Sweden. European Child & Adolescent Psychiatry. 2016;25(10):1103-11.

44. Rostila M, Berg L, Saarela J, Kawachi I, Hjern A. Experience of sibling death in childhood and risk of psychiatric care in adulthood: a national cohort study from Sweden. European Child & Adolescent Psychiatry. 2019:01.

45. Rostila M, Saarela J, Kawachi I. The forgotten griever: A nationwide follow-up study of mortality subsequent to the death of a sibling. American Journal of Epidemiology. 2012;176(4):338-46.

46. Rostila M, Saarela J, Kawachi I. "The psychological skeleton in the closet": mortality after a sibling's suicide. Social psychiatry and psychiatric epidemiology. 2014;49(6):919-27.

47. Rostila M, Saarela J, Kawachi I, Hjern A. Testing the anniversary reaction: causal effects of bereavement in a nationwide follow-up study from Sweden. European Journal of Epidemiology. 2015;30(3):239-47.

48. Russell AE, Heron J, Gunnell D, Ford T, Gibran H, Joinson C, et al. Pathways between early‐life adversity and adolescent self‐harm: the mediating role of inflammation in the Avon Longitudinal Study of Parents and Children. Journal of Child Psychology and Psychiatry. 2019;60(10):1094-103.

49. Sherkat DE, Reed MD. The Effects of Religion and Social Support on Self-Esteem and Depression among the Suddenly Bereaved. Social Indicators Research. 1992;26(3):259-75.

50. Stelle CD, Uchida M. The Stability and Change in the Social Support Networks of Widowers Following Spousal Bereavement. Journal of Men's Studies. 2004;13(1):85.

51. Van Der Hoek W, Konradsen F. Risk factors for acute pesticide poisoning in Sri Lanka. Tropical Medicine and International Health. 2005;10(6):589-96.

52. Vargas LA, Loya F, Hodde-Vargas J. Exploring the multidimensional aspects of grief reactions. American Journal of Psychiatry. 1989;146(11):1484-8.

53. Wan Y, Chen R, Ma S, McFeeters D, Sun Y, Hao J, et al. Associations of adverse childhood experiences and social support with self-injurious behaviour and suicidality in adolescents. British Journal of Psychiatry. 2019;214(3):146-52.

54. Weinberg RJ, Dietz LJ, Stoyak S, Melhem NM, Porta G, Payne MW, et al. A prospective study of parentally bereaved youth, caregiver depression, and body mass index. Journal of Clinical Psychiatry. 2013;74(8):834-40.

55. Zetumer S, Young I, Shear MK, Skritskaya N, Lebowitz B, Simon N, et al. The impact of losing a child on the clinical presentation of complicated grief. Journal of Affective Disorders. 2014;170:15-21.

56. Zisook S, Shuchter SR. Uncomplicated bereavement. Journal of Clinical Psychiatry. 1993;54(10):365-72.

57. Zisook S, Shuchter SR, Lyons LE. Predictors of psychological reactions during the early stages of widowhood. Psychiatric Clinics of North America. 1987;10(3):355-68.

## Lifetime prevalence of exposure or outcome measurement

1. Agrawal A, Constantino AM, Bucholz KK, Glowinski A, Madden PAF, Heath AC, et al. Characterizing Alcohol Use Disorders and Suicidal Ideation in Young Women. Journal of Studies on Alcohol and Drugs. 2013;74(3):406-12.

2. Atay IM, Eren I, Gundogar D. The prevalence of death ideation and attempted suicide and the associated risk factors in Isparta, Turkey. Turk psikiyatri dergisi = Turkish journal of psychiatry. 2012;23(2):89-98.

3. Atwoli L, Nock MK, Williams DR, Stein DJ. Association between parental psychopathology and suicidal behavior among adult offspring: Results from the cross-sectional South African Stress and Health survey. BMC Psychiatry. 2014;14(1):65.

4. Ballard ED, Cui L, Vandeleur C, Castelao E, Zarate CA, Jr., Preisig M, et al. Familial Aggregation and Coaggregation of Suicide Attempts and Comorbid Mental Disorders in Adults. JAMA Psychiatry. 2019:27.

5. Benjet C, Menendez D, Albor Y, Borges G, Orozco R, Medina-Mora ME. Adolescent Predictors of Incidence and Persistence of Suicide-Related Outcomes in Young Adulthood: A Longitudinal Study of Mexican Youth. Suicide & Life-Threatening Behavior. 2018;48(6):755-66.

6. Blasco MJ, Vilagut G, Almenara J, Roca M, Piqueras JA, Gabilondo A, et al. Suicidal Thoughts and Behaviors: Prevalence and Association with Distal and Proximal Factors in Spanish University Students. Suicide & Life Threatening Behavior. 2018:23.

7. Borowsky IW, Resnick MD, Ireland M, Blum RW. Suicide attempts among American Indian and Alaska native youth: Risk and protective factors. Archives of Pediatrics and Adolescent Medicine. 1999;153(6):573-80.

8. Brent DA, Bridge J, Johnson BA, Connolly J. Suicidal behavior runs in families: A controlled family study of adolescent suicide victims. Archives of General Psychiatry. 1996;53(12):1145-52.

9. Brick LA, Marraccini ME, Micalizzi L, Benca-Bachman CE, Knopik VS, Palmer RHC. Overlapping genetic effects between suicidal ideation and neurocognitive functioning. Journal of Affective Disorders. 2019;249:104-11.

10. Burke AKP, Galfalvy HP, Everett BBA, Currier DP, Zelazny JMPH, Oquendo MAMD, et al. Effect of Exposure to Suicidal Behavior on Suicide Attempt in a High-Risk Sample of Offspring of Depressed Parents. Journal of the American Academy of Child and Adolescent Psychiatry. 2010;49(2):114.

11. Campos RC, Holden RR, Santos S. Exposure to suicide in the family: Suicide risk and psychache in individuals who have lost a family member by suicide. Journal of clinical psychology. 2018;74(3):407-17.

12. Cerel J, Maple M, van de Venne J, Moore M, Flaherty C, Brown M. Exposure to suicide in the community: Prevalence and correlates in one U.S. State. Public Health Reports. 2016;131(1):100-7.

13. Chen JI, Bozzay ML, Monahan MF, Gryglewicz K, Romero G, Steding LH, et al. Life after loss: Comparing student service member/veteran and civilian mental health characteristics among individuals exposed to death by suicide. Journal of American college health : J of ACH. 2018:1-25.

14. Chen JI, Bozzay ML, Monahan MF, Gryglewicz K, Romero G, Steding LH, et al. Life after loss: Comparing student service member/veteran and civilian mental health characteristics among individuals exposed to death by suicide. Journal of American College Health. 2019;67(3):197-206.

15. Copeland M, Siennick SE, Feinberg ME, Moody J, Ragan DT. Social Ties Cut Both Ways: Self-Harm and Adolescent Peer Networks. Journal of Youth & Adolescence. 2019:15.

16. Coppersmith DDL, Nada-Raja S, Beautrais AL. Non-suicidal self-injury and suicide attempts in a New Zealand birth cohort. Journal of Affective Disorders. 2017;221:89-96.

17. Crosby AE, Sacks JJ. Exposure to suicide: Incidence and association with suicidal ideation and behavior: United States, 1994. Suicide and Life-Threatening Behavior. 2002;32(3):321-8.

18. Darre T, Consuela KAC, Saka B, Djiwa T, Ekouevi KD, Napo-Koura G. Suicidal ideation and suicide attempts in subjects aged 15-19 in Lome (Togo). BMC Research Notes. 2019;12(1):187.

19. De Araujo RMF, Mazzochi L, Lara DR, Ottoni GL. Thinking about dying and trying and intending to Die: Results on suicidal behavior from a large web-based sample. Journal of Clinical Psychiatry. 2015;76(3):e359-e65.

20. De Groot MH, De Keijser J, Neeleman J. Grief shortly after suicide and natural death: A comparative study among spouses and first-degree relatives. Suicide and Life-Threatening Behavior. 2006;36(4):418-31.

21. De Leo D, Heller T. Social modeling in the transmission of suicidality. Crisis. 2008;29(1):11-9.

22. Doyle L, Treacy MP, Sheridan A. Self-harm in young people: Prevalence, associated factors, and help-seeking in school-going adolescents. International journal of mental health nursing. 2015;24(6):485-94.

23. Feigelman W, Gorman BS. Assessing the Effects of Peer Suicide on Youth Suicide. Suicide and Life-Threatening Behavior. 2008;38(2):181-94.

24. Fleming TM, Merry SN, Robinson EM, Denny SJ, Watson PD. Self-reported suicide attempts and associated risk and protective factors among secondary school students in New Zealand. The Australian and New Zealand journal of psychiatry. 2007;41(3):213-21.

25. Georgiades K, Boylan K, Duncan L, Wang L, Colman I, Rhodes AE, et al. Prevalence and Correlates of Youth Suicidal Ideation and Attempts: Evidence from the 2014 Ontario Child Health Study. Canadian Journal of Psychiatry - Revue Canadienne de Psychiatrie. 2019;64(4):265-74.

26. Ghaffari M, Ahmadi A, Abedi MR, Fatehizade M, Baghban I. Impulsivity, substance abuse, and family/friends history of suicide attempts in university students with and without suicidal ideation. Iranian Journal of Psychiatry and Behavioral Sciences. 2011;5(2):99-105.

27. Glowinski AL, Bucholz KK, Nelson EC, Fu Q, et al. Suicide attempts in an adolescent female twin sample. Journal of the American Academy of Child and Adolescent Psychiatry. 2001;40(11):1300-7.

28. Goodwin RD, Beautrais AL, Fergusson DM. Familial transmission of suicidal ideation and suicide attempts: Evidence from a general population sample. Psychiatry Research. 2004;126(2):159-65.

29. Gould MS, Lake AM, Kleinman M, Galfalvy H, Chowdhury S, Madnick A. Exposure to suicide in high schools: Impact on serious suicidal ideation/behavior, depression, maladaptive coping strategies, and attitudes toward help-seeking. International Journal of Environmental Research and Public Health. 2018;15(3):455.

30. Grossman DC, Milligan BC, Deyo RA. Risk factors for suicide attempts among Navajo adolescents. American Journal of Public Health. 1991;81(Jul 91):870-4.

31. Guerreiro DF, Sampaio D, Figueira ML, Madge N. Self-Harm in Adolescents: A Self-Report Survey in Schools from Lisbon, Portugal. Archives of Suicide Research. 2017;21(1):83-99.

32. Gureje O, Kola L, Uwakwe R, Udofia O, Wakil A, Afolabi E. The profile and risks of suicidal behaviours in the Nigerian Survey of Mental Health and Well-Being. Psychological Medicine. 2007;37(6):821-30.

33. Gureje O, Oladeji B, Hwang I, Chiu WT, Kessler RC, Sampson NA, et al. Parental psychopathology and the risk of suicidal behavior in their offspring: Results from the World Mental Health surveys. Molecular Psychiatry. 2011;16(12):1221-33.

34. Hamdan S, Melhem N, Orbach I, Farbstein I, El-Haib M, Apter A, et al. Risk factors for suicide attempt in an Arab kindred. Journal of Affective Disorders. 2011;132(1-2):247-53.

35. Hargus E, Hawton K, Rodham K. Distinguishing between subgroups of adolescents who self-harm. Suicide and Life-Threatening Behavior. 2009;39(5):518-37.

36. Ho TP, Leung PW, Hung SF, Lee CC, Tang CP. The mental health of the peers of suicide completers and attempters. J Child Psychol Psychiatry. 2000;41(3):301-8.

37. Hom MA, Stanley IH, Gutierrez PM, Joiner TE. Exploring the association between exposure to suicide and suicide risk among military service members and veterans. Journal of Affective Disorders. 2017;207:327-35.

38. Hom MA, Stanley IH, Spencer-Thomas S, Joiner TE. Exposure to suicide and suicide bereavement among women firefighters: Associated suicidality and psychiatric symptoms. J Clin Psychol. 2018;74(12):2219-37.

39. Jegannathan B, Kullgren G. Gender differences in suicidal expressions and their determinants among young people in Cambodia, a post-conflict country. BMC Psychiatry. 2011;11:47.

40. Kenneally LB, Szucs A, Szanto K, Dombrovski AY. Familial and social transmission of suicidal behavior in older adults. Journal of Affective Disorders. 2019;245:589-96.

41. Kim CD, Seguin M, Therrien N, Riopel G, Chawky N, Lesage AD, et al. Familial aggregation of suicidal behavior: A family study of male suicide completers from the general population. American Journal of Psychiatry. 2005;162(5):1017-9.

42. Kirmayer LJ, Malus M, Boothroyd LJ. Suicide attempts among Inuit youth: A community survey of prevalence and risk factors. Acta Psychiatrica Scandinavica. 1996;94(1):8-17.

43. Knipe DW, Gunnell D, Pearson M, Jayamanne S, Pieris R, Priyadarshana C, et al. Attempted suicide in Sri Lanka - An epidemiological study of household and community factors. Journal of Affective Disorders. 2018;232:177-84.

44. Lee H, Seol KH, Kim JW. Age and sex-related differences in risk factors for elderly suicide: Differentiating between suicide ideation and attempts. International Journal of Geriatric Psychiatry. 2018;33(2):e300-e6.

45. Lee M-A, Kim S, Shim E-J. Exposure to suicide and suicidality in Korea: Differential effects across men and women? International Journal of Social Psychiatry. 2012;59(3):224-31.

46. Liu XC, Chen H, Liu ZZ, Wang JY, Jia CX. Prevalence of suicidal behaviour and associated factors in a large sample of Chinese adolescents. Epidemiology & Psychiatric Science. 2019;28(3):280-9.

47. Liu ZZ, Wang ZY, Bo QG, Qi ZB, Xu RJ, Jia CX, et al. Suicidal behaviours among Chinese adolescents exposed to suicide attempt or death. Epidemiology & Psychiatric Science. 2018:1-9.

48. Lyu J, Wang Y, Shi H, Zhang J. Early warnings for suicide attempt among Chinese rural population. Journal of Affective Disorders. 2018;238:353-8.

49. Mars B, Heron J, Klonsky ED, Moran P, O'Connor RC, Tilling K, et al. What distinguishes adolescents with suicidal thoughts from those who have attempted suicide? A population-based birth cohort study. Journal of Child Psychology & Psychiatry & Allied Disciplines. 2019;60(1):91-9.

50. McMahon EM, Reulbach U, Corcoran P, Keeley HS, Perry IJ, Arensman E. Factors associated with deliberate self-harm among Irish adolescents. Psychological Medicine. 2010;40(11):1811-9.

51. Medina CO, Jegannathan B, Dahlblom K, Kullgren G. Suicidal expressions among young people in Nicaragua and Cambodia: A cross-cultural study. BMC Psychiatry. 2012;12(1):28.

52. Mitchell KJ, Turner HA, Jones LM. Youth Exposure to Suicide Attempts: Relative Impact on Personal Trauma Symptoms. American Journal of Preventive Medicine. 2019;56(1):109-15.

53. Nojomi M, Malakouti SK, Bolhari J, Poshtmashhadi M. A predictor model for suicide attempt: Evidence from a population-based study. Archives of Iranian Medicine. 2007;10(4):452-8.

54. O'Connor RC, Rasmussen S, Hawton K. Distinguishing adolescents who think about self-harm from those who engage in self-harm. The British Journal of Psychiatry. 2012;200(4):330-5.

55. Pfeffer CR, Normandin L, Kakuma T. Suicidal children grow up: Suicidal behavior and psychiatric disorders among relatives. Journal of the American Academy of Child and Adolescent Psychiatry. 1994;33(8):1087.

56. Pfeffer CR, Normandin L, Kakuma T. Suicidal children grow up: Relations between family psychopathology and adolescents' lifetime suicidal behavior. Journal of Nervous and Mental Disease. 1998;186(5):269-75.

57. Portzky G, Wilde E-J, Heeringen K. Deliberate self-harm in young people: differences in prevalence and risk factors between The Netherlands and Belgium. European Child & Adolescent Psychiatry. 2008;17(3):179-86.

58. Reyes-Portillo JA, Lake AM, Kleinman M, Gould MS. The Relation between Descriptive Norms, Suicide Ideation, and Suicide Attempts among Adolescents. Suicide & Life-Threatening Behavior. 2019;49(2):535-46.

59. Rihmer Z, Gonda X, Torzsa P, Kalabay L, Akiskal HS, Eory A. Affective temperament, history of suicide attempt and family history of suicide in general practice patients. Journal of Affective Disorders. 2013;149(1-3):350-4.

60. Song IH, Kwon SW, Kim JE. Association Between Suicidal Ideation and Exposure to Suicide in Social Relationships Among Family, Friend, and Acquaintance Survivors in South Korea. Suicide and Life-Threatening Behavior. 2015;45(3):376.

61. Sorenson SB, Rutter CM. Transgenerational patterns of suicide attempt. Journal of Consulting and Clinical Psychology. 1991;59(Dec 91):861-73.

62. Spiwak R, Pagura J, Bolton JM, Elias B, Beesdo-Baum K, Lieb R, et al. Childhood Exposure to Caregiver Suicidal Behavior and Risk for Adult Suicide Attempts: Findings from a National Survey. Archives of Suicide Research. 2011;15(4):313-26.

63. Tomori M, Kienhorst CW, de Wilde EJ, van den Bout J. Suicidal behaviour and family factors among Dutch and Slovenian high school students: a comparison. Acta psychiatrica Scandinavica. 2001;104(3):198-203.

64. Toros F, Bilgin NG, Sasmaz T, Bugdayci R, Camdeviren H. Suicide attempts and risk factors among children and adolescents. Yonsei Medical Journal. 2004;45(3):367-74.

65. Van Rijsselberghe L, Portzky G, Van Heeringen C. Self harm in adolescents in Flanders. Tijdschrift voor Psychiatrie. 2009;51(9):629-40.

66. Wang J, Dong Y, Qi J-L, Wang H-B, Zhang Q. Related factors of suicide ideation in military personnel. Chinese Mental Health Journal. 2017;31(6):485-9.

67. Wang YG, Chen S, Xu ZM, Shen ZH, He XY, Cao RF, et al. Family history of suicide and high motor impulsivity distinguish suicide attempters from suicide ideators among college students. Journal of Psychiatric Research. 2017;90:21-5.

68. Wilcox HC, Kuramoto SJ, Lichtenstein P, Langstrom N, Brent DA, Runeson B. Psychiatric Morbidity, Violent Crime, and Suicide Among Children and Adolescents Exposed to Parental Death. Journal of the American Academy of Child and Adolescent Psychiatry. 2010;49(5):514-23.

69. Wong JPS, Stewart SM, Ho SY, Rao U, Lam TH. Exposure to Suicide and Suicidal Behaviors among Hong Kong Adolescents. Social Science & Medicine. 2005;61(3):591-9.

70. Wyman PA, Pickering TA, Pisani AR, Rulison K, Karen SC, Hartley C, et al. Peer‐adult network structure and suicide attempts in 38 high schools: implications for network‐informed suicide prevention. Journal of Child Psychology and Psychiatry. 2019;60(10):1065-75.

71. Yoder KA. Comparing suicide attempters, suicide ideators and nonsuicidal homeless and runaway adolescents. Suicide and Life-Threatening Behavior. 1999;29(1):25-36.

72. Ystgaard M, Reinholdt NP, Husby J, Mehlum L. Deliberate self harm in adolescents. Tidsskrift for den Norske Laegeforening. 2003;123(16):2241-5.

73. Zhang J, Zhou L. Suicidal ideation, plans, and attempts among rural young Chinese: The effect of suicide death by a family member or friend. Community Mental Health Journal. 2011;47(5):506-12.

## Overlapping populations and/or time periods

1. Abrutyn S, Mueller AS. Are Suicidal Behaviors Contagious in Adolescence? Using Longitudinal Data to Examine Suicide Suggestion. American Sociological Review. 2014;79(2):211-27.

2. Agerbo E. Risk of suicide and spouse's psychiatric illness or suicide: nested case-control study. British Medical Journal. 2003;327(7422):1025-6.

3. Agerbo E, Nordentoft M, Mortensen PB. Familial, psychiatric, and socioeconomic risk factors for suicide in young people: Nested case-control study. British Medical Journal. 2002;325(7355):74.

4. Ahmadi A, Mohammadi R, Schwebel DC, Yeganeh N, Soroush A, Bazargan-Hejazi S. Familial risk factors for self-immolation: A case-control study. Journal of Women's Health. 2009;18(7):1025-31.

5. Ahmadi A, Schwebel DC, Bazargan-Hejazi S, Taliee K, Karim H, Mohammadi R. Self-immolation and its adverse life-events risk factors: results from an Iranian population. Journal of injury & violence research. 2015;7(1):13-8.

6. Ali MM, Dwyer DS, Rizzo JA. The social contagion effect of suicidal behavior in adolescents: Does it really exist? Journal of Mental Health Policy and Economics. 2011;14(1):3-12.

7. Asberg M, Runeson B. Family history of suicide among suicide victims. American Journal of Psychiatry. 2003;160(8):1525-6.

8. Brent DA, Oquendo M, Birmaher B, Greenhill L, Kolko D, Stanley B, et al. Familial pathways to early-onset suicide attempt: Risk for suicidal behavior in offspring of mood-disordered suicide attempters. Archives of General Psychiatry. 2002;59(9):801-7.

9. Brent DA, Perper JA, Moritz G, Liotus L, Schweers J, Canobbio R. Major depression or uncomplicated bereavement? A follow-up of youth exposed to suicide. Journal of the American Academy of Child and Adolescent Psychiatry. 1994;33(2):231-9.

10. Brent DA, Perper JA, Moritz G, Liotus L, Schweers J, Roth C, et al. Psychiatric impact of the loss of an adolescent sibling to suicide. Journal of Affective Disorders. 1993;28(4):249-56.

11. Cerel J, Roberts TA. Suicidal behavior in the family and adolescent risk behavior. Journal of Adolescent Health. 2005;36(4):352.

12. Cerel J, Roberts TA, Nilsen WJ. Peer suicidal behavior and adolescent risk behavior. Journal of Nervous and Mental Disease. 2005;193(4):237-43.

13. Cerel J, Van De Venne JG, Moore MM, Maple MJ, Flaherty C, Brown MM. Veteran exposure to suicide: Prevalence and correlates. Journal of Affective Disorders. 2015;179:82-7.

14. Cheng CC, Yen WJ, Chang WT, Wu KC, Ko MC, Li CY. Risk of adolescent offspring's completed suicide increases with prior history of their same-sex parents' death by suicide. Psychol Med. 2014;44(9):1845-54.

15. Garcia-Valencia J, Palacio-Acosta C, Diago J, Zapata C, Lopez G, Ortiz J, et al. Adverse life events and suicide: A case-control study of psychological autopsy in Medellin, Colombia. Eventos vitales adversos y suicidio: Un estudio de autopsia psicologica en Medellin, Colombia. 2008;37(1):11-28.

16. Guldin MB, Li J, Pedersen HS, Obel C, Agerbo E, Gissler M, et al. Incidence of suicide among persons who had a parent who died during their childhood a population-based cohort study. JAMA Psychiatry. 2015;72(12):1227-34.

17. Hill RM, Oosterhoff B, Kaplow JB. Prospective identification of adolescent suicide ideation using classification tree analysis: Models for community-based screening. Journal of Consulting and Clinical Psychology. 2017;85(7):702.

18. Liu RX. Vulnerability to Friends’ Suicide Influence: The Moderating Effects of Gender and Adolescent Depression. Journal of Youth and Adolescence. 2006;35(3):454.

19. Melhem NM, Brent DA, Ziegler M, Iyengar S, Kolko D, Oquendo M, et al. Familial pathways to early-onset suicidal behavior: Familial and individual antecedents of suicidal behavior. American Journal of Psychiatry. 2007;164(9):1364-70.

20. Mueller AS, Abrutyn S. Suicidal disclosures among friends: using social network data to understand suicide contagion. Journal of Health & Social Behavior. 2015;56(1):131-48.

21. Mueller AS, Abrutyn S, Stockton C. Can Social Ties Be Harmful? Examining the Spread of Suicide in Early Adulthood. Sociological Perspectives. 2015;58(2):204-22.

22. Nanayakkara S, Misch D, Chang L, Henry D. Depression and exposure to suicide predict suicide attempt. Depression and Anxiety. 2013;30(10):991-6.

23. Petersen L, Sorensen TIA, Andersen PK, Mortensen PB, Hawton K. Genetic and familial environmental effects on suicide--an adoption study of siblings. PloS one. 2013;8(10):e77973.

24. Petersen L, Sorensen TIA, Andersen PK, Mortensen PB, Hawton K. Genetic and familial environmental effects on suicide attempts: A study of Danish adoptees and their biological and adoptive siblings. Journal of Affective Disorders. 2014;155:273-7.

25. Qin P, Agerbo E, Mortensen PB. Suicide risk in relation to family history of completed suicide and psychiatric disorders: a nested case-control study based on longitudinal registers. Lancet. 2002;12:1126-30.

26. Qin P, Mortensen PB. The impact of parental status on the risk of completed suicide. Archives of general psychiatry. 2003;60(8):797-802.

27. Randall JR, Nickel NC, Colman I. Contagion from peer suicidal behavior in a representative sample of American adolescents. Journal of Affective Disorders. 2015;186:219-25.

28. Rubenowitz E, Waern M, Wilhelmson K, Allebeck P. Life events and psychosocial factors in elderly suicides--a case-control study. Psychological medicine. 2001;31(7):1193-202.

29. Sorensen HJ, Mortensen EL, Wang AG, Juel K, Silverton L, Mednick SA. Suicide and mental illness in parents and risk of suicide in offspring: a birth cohort study. Social psychiatry and psychiatric epidemiology. 2009;44(9):748-51.

30. Zamora-Kapoor A, Nelson LA, Barbosa-Leiker C, Comtois KA, Walker LR, Buchwald DS. Suicidal ideation in American Indian/Alaska Native and White adolescents: The role of social isolation, exposure to suicide, and overweight. American Indian and Alaska native mental health research (Online). 2016;23(4):86-100.

## Missing information

1. Statham DJ, Heath AC, Madden PAF, et al. Suicidal behaviour: An epidemiological and genetic study. *Psychological Medicine.* 1998;28(4):839-855.

2. Watkins RL, Gutierrez PM. The relationship between exposure to adolescent suicide and subsequent suicide risk. *Suicide and Life-Threatening Behavior.* 2003;33(1):21-32.

## Outlier

1. Martiello MA, Boncompagni G, Lacangellera D, Corlito G. Risk factors for suicide in rural italy: A case-control study. *Social Psychiatry & Psychiatric Epidemiology.* 2019;54(5):607-616.
2. Rasouli N, Malakouti SK, Rezaeian M, Saberi SM, Nojomi M, De Leo D, et al. Risk Factors of Suicide Death Based on Psychological Autopsy Method; a Case-Control Study. Arch. 2019;7(1):e50.

## Included studies

1. Agerbo E, Mortensen PB, Qin P. Suicide risk in relation to socioeconomic, demographic, psychiatric, and familial factors: a national register-based study of all suicides in Denmark, 1981-1997. American Journal of Psychiatry. 2003;160(4):765-72.

2. Ahmadi A, Mohammadi R, Almasi A, Amini-Saman J, Sadeghi-Bazargani H, Bazargan-Hejazi S, et al. A case-control study of psychosocial risk and protective factors of self-immolation in Iran. Burns. 2015;41(2):386-93.

3. Almeida OP, Draper B, Snowdon J, Lautenschlager NT, Pirkis J, Byrne G, et al. Factors associated with suicidal thoughts in a large community study of older adults. The British Journal of Psychiatry. 2012;201(6):466-72.

4. Brent DA, Melhem NM, Oquendo M, Burke A, Birmaher B, Stanley B, et al. Familial pathways to early-onset suicide attempt: A 5.6-year prospective study. JAMA Psychiatry. 2015;72(2):160-8.

5. Chachamovich E, Kirmayer LJ, Haggarty JM, Cargo M, McCormick R, Turecki G. Suicide among Inuit: Results from a large, epidemiologically representative follow-back study in Nunavut. Canadian Journal of Psychiatry. 2015;60(6):268-75.

6. Chan S, Denny S, Fleming T, Fortune S, Peiris-John R, Dyson B. Exposure to suicide behaviour and individual risk of self-harm: Findings from a nationally representative New Zealand high school survey. Australian and New Zealand Journal of Psychiatry. 2018;52(4):349-56.

7. Cheng ATA, Chen THH, Chen C-C, Jenkins R. Psychosocial and psychiatric risk factors for suicide: Case-control psychological autopsy study. The British Journal of Psychiatry. 2000;177:360-5.

8. Christiansen E, Goldney RD, Beautrai AL, Agerbo E. Youth suicide attempts and the dose–response relationship to parental risk factors: a population-based study. Psychological Medicine. 2011;41(2):313-9.

9. Conner KR, Phillips MR, Meldrum SC. Predictors of low-intent and high-intent suicide attempts in rural China. American Journal of Public Health. 2007;97(10):1842-6.

10. Foster T, Gillespie K, McClelland R, Patterson C. Risk factors for suicide independent of DSM-III-R Axis I disorder. Case-control psychological autopsy study in Northern Ireland. Br J Psychiatry. 1999;175:175-9.

11. Garfinkel BD, Froese A, Hood J. Suicide attempts in children and adolescents. Am J Psychiatry. 1982;139(10):1257-61.

12. Giupponi G, Innamorati M, Baldessarini RJ, De Leo D, de Giovannelli F, Pycha R, et al. Factors associated with suicide: Case-control study in South Tyrol. Comprehensive Psychiatry. 2018;80:150-4.

13. Gould MS, Fisher P, Parides M, Flory M, Shaffer D. Psychosocial risk factors of child and adolescent completed suicide. Archives of General Psychiatry. 1996;53(12):1155-62.

14. Gravseth HM, Mehlum L, Bjerkedal T, Kristensen P. Suicide in young Norwegians in a life course perspective: population-based cohort study. J Epidemiol Community Health. 2010;64(5):407-12.

15. Gray D, Coon H, McGlade E, Callor WB, Byrd J, Viskochil J, et al. Comparative analysis of suicide, accidental, and undetermined cause of death classification. Suicide & life-threatening behavior. 2014;44(3):304-16.

16. Hishinuma ES, Smith MD, McCarthy K, Lee M, Goebert DA, Sugimoto-Matsuda JJ, et al. Longitudinal Prediction of Suicide Attempts for a Diverse Adolescent Sample of Native Hawaiians, Pacific Peoples, and Asian Americans. Archives of Suicide Research. 2018;22(1):67-90.

17. Hu N, Li J, Glauert RA, Taylor CL. Influence of exposure to perinatal risk factors and parental mental health related hospital admission on adolescent deliberate self-harm risk. European Child & Adolescent Psychiatry. 2017;26(7):791-803.

18. Jollant F, Malafosse A, Docto R, Macdonald C. A pocket of very high suicide rates in a non-violent, egalitarian and cooperative population of South-East Asia. Psychological Medicine. 2014;44(11):2323-9.

19. Katibeh P, Inaloo S, Shokrpour N, Dashti H, Alavi Shoostari A. A Survey of the Suicidal Attempt Risk Factors in Adolescents in Southern Iran. Int J School Health. 2018;5(1):e12783.

20. Lee KY, Li CY, Chang KC, Lu TH, Chen YY. Age at Exposure to Parental Suicide and the Subsequent Risk of Suicide in Young People. Crisis: Journal of Crisis Intervention & Suicide. 2018;39(1):27-36.

21. Lewinsohn PM, Rohde P, Seeley JR. Psychosocial risk factors for future adolescent suicide attempts. J Consult Clin Psychol. 1994;62(2):297-305.

22. Liu BP, Qin P, Jia CX. Behavior Characteristics and Risk Factors for Suicide Among the Elderly in Rural China. Journal of Nervous & Mental Disease. 2018;206(3):195-201.

23. Maniam T. Family characteristics of suicides in Cameron Highlands: a controlled study. The Medical journal of Malaysia. 1994;49(3):247-51.

24. Mercy JA, Kresnow MJ, O'Carroll PW, Lee RK, Powell KE, Potter LB, et al. Is suicide contagious? A study of the relation between exposure to the suicidal behavior of others and nearly lethal suicide attempts. American Journal of Epidemiology. 2001;154(2):120-7.

25. Mittendorfer-Rutz E, Rasmussen F, Wasserman D. Familial clustering of suicidal behaviour and psychopathology in young suicide attempters. A register-based nested case control study. Social psychiatry and psychiatric epidemiology. 2008;43(1):28-36.

26. Nrugham L, Larsson B, Sund AM. Predictors of suicidal acts across adolescence: Influences of familial, peer and individual factors. Journal of Affective Disorders. 2008;109(1-2):35-45.

27. Palacio C, Garcia J, Diago J, Zapata C, Lopez G, Ortiz J, et al. Identification of Suicide Risk Factors in Medellin, Colombia: A Case-Control Study of Psychological Autopsy in a Developing Country. Archives of Suicide Research. 2007;11(3):297-308.

28. Phillips MR, Yang G, Zhang Y, Wang L, Ji H, Zhou M. Risk factors for suicide in China: A national case-control psychological autopsy study. Lancet. 2002;360(9347):1728-36.

29. Brent DA, Moritz G, Bridge J, Perper J, Canobbio R. Long-term impact of exposure to suicide: A three-year controlled follow-up. Journal of the American Academy of Child and Adolescent Psychiatry. 1996;35(5):646.

30. Brent DA, Moritz G, Bridge J, Perper J, Canobbio R. The Impact of Adolescent Suicide on Siblings and Parents: A Longitudinal Follow-Up. Suicide and Life-Threatening Behavior. 1996;26(3):253-9.

31. Swanson SA, Colman I. Association between exposure to suicide and suicidality outcomes in youth. CMAJ. 2013;185(10):870-7.

32. Thompson MP, Light LS. Examining Gender Differences in Risk Factors for Suicide Attempts Made 1 and 7 Years Later in a Nationally Representative Sample. Journal of Adolescent Health. 2011;48(4):391-7.

33. Tidemalm D, Runeson B, Waern M, Frisell T, Carlström E, Lichtenstein P, et al. Familial clustering of suicide risk: a total population study of 11.4 million individuals. Psychological Medicine. 2011;41(12):2527-34.

34. Vijayakumar L, Rajkumar S. Are risk factors for suicide universal? A case-control study in India. Acta Psychiatrica Scandinavica. 1999;99(6):407-11.
