# Supplementary material for: Association of suicidal behavior with exposure to suicide and suicide attempt: A systematic review and multilevel meta-analysis
Source: PLoS Med. 2020 Mar 31;17(3):e1003074. doi: 10.1371/journal.pmed.1003074 (PMC7108695; doi:10.1371/journal.pmed.1003074)
Supplement: Attachment — Submitted filename: hill.pdf [file pmed.1003074.s011.pdf]

This paper presents a systematic review with multi–level meta–analysis of studies of suicide.

I was asked for a statistical report and I interpret that to include all aspects of the design and conduct of the study.

# Points of detail

**Page 6** Here the authors refer to 'multilevel (three–level)'. I wonder whether in fact the term multilevel might be preferred in the title as the more general term? I have reviewed many studies which could have benefited from a multilevel meta–analysis but can count on the fingers of one hand those which actually did it. Making it easy to find the more generic term might help others to find this study as an example of how it is done.

**Page 10** I am not sure that the arbitrary cut–offs for heterogeneity are really helpful. In epidemiological studies high levels of heterogeneity are to be expected and the important thing is to describe it and, if possible, say why it is there.

**Page 13** I have to say that I would normally object quite strongly to excluding outliers but an odds ratio of 36.4 is so extreme that I can see the authors' point here. I suppose we have no way of knowing what happened there? Extreme odds ratios are often due to separation or quasi–separation.

**Page 13** I think there is a (k = ??) missing after the phrase 'suicide attempt' in one place.

**Page 25** Given the problems in detecting small study effects in the presence of heterogeneity I would hesitate to read too much into $p = 0.06$ here.

**Page 25** '[. . . ] not suicidal ideation [. . . ] CI = 0.97'. Well, that is strictly true but I think this is slightly too strong. The authors found no evidence of association but the estimate is quite imprecise. My comment also applies to the same result in the abstract and to other results about ideation later. How about 'there was no evidence of an association with suicidal ideation'?

**Supplementary S2** Typo, ¿ instead of >

**Supplementary S3** That is a very impressive and helpful list of studies consulted.

**Supplementary S6** Unfortunately the file contained a duplicate of Figure S7.

# Points of more substance

## Age

The problem with the way age is incorporated into the analysis is that the coverage of studies overlaps substantially and many of the ones labelled adult also include youths and vice versa. So the comment about age on page 25 may reflect the imprecision in the classification of the moderator variable. I think I might mention this as a limitation. This is of course an ecological analysis and so perhaps less crucial.

## Multiple exposure

On reading that 21% have experienced suicide in someone known to them the statistical reader immediately thinks that, conditional on independence, that means about 4.4% have experienced it twice, and indeed about 1% three or more times. There is a brief mention on page 36 of this. Would it be helpful if there are studies which have examined this to mention which they are and perhaps give a narrative summary?

# Summary

This is a meticulous and comprehensive report of a very substantial piece of work on which I have made a few comments of detail.

Michael Dewey
