# Supplementary material for: Association of suicidal behavior with exposure to suicide and suicide attempt: A systematic review and multilevel meta-analysis
Source: PLoS Med. 2020 Mar 31;17(3):e1003074. doi: 10.1371/journal.pmed.1003074 (PMC7108695; doi:10.1371/journal.pmed.1003074)
Supplement: Attachment — Submitted filename: Response to reviewers.docx [file pmed.1003074.s012.docx]

**Requests from the editors**

*1. Data availability statement: Thank you for your willingness to make your data available as Supporting Information.  Please note in the Data Availability Statement and the Methods that the study data are included as Supporting Information, and reference the file (e.g. S1 Table).*

**These data are provided as S1 Data (xls file). We have updated the Methods (ln 223) to:**

*Study-level data are provided as S1 Data.*

**We have also updated the Data Availability Statement (ln 972-5) to:**

*All relevant data are within the manuscript and its Supporting information Files. Summary data used in the analyses are provided as S1 Data.*

*2. Title: Please revise the title to: “Association of suicidal behavior with exposure to suicide and suicide attempt: A systematic review and multilevel meta-analysis*

**The title has been revised accordingly.**

*3. Abstract: Please include methods of assessing risk of bias in the abstract Methods and Findings section.*

**The Methods and Findings section has been updated to include the following (ln 19-20):**

*We assessed study quality using the National Heart, Lung, and Blood Institute quality assessment tool for observational studies*

*4. Abstract: Please include numbers of studies and participants for each of the main outcomes described in the abstract Methods and Findings section.*

**The Methods and Findings section has been updated accordingly. For example (ln 29-33):**

*Exposure to suicide was associated with increased odds of suicide (11 studies, N=13,464,582; OR=3.23, 95%CI=2.32 to 4.51, P<0.001) and suicide attempt (10 studies, N=121,836; OR=2.91, 95%CI=2.01 to 4.23, P<0.001) but no evidence of an association was observed for suicidal ideation outcomes (2 studies, N=43,354; OR=1.85, 95%CI=0.97 to 3.51, P=0.06).*

*5. Abstract: Please quantify the main results with both 95% CIs and p values.*

**The Methods and Findings section has been updated accordingly, as demonstrated in our response to the previous comment.**

*6. Author Summary: At this stage, we ask that you include a short, non-technical Author Summary of your research to make findings accessible to a wide audience that includes both scientists and non-scientists.*

**The summary has been provided accordingly (ln 52-74)**

*7. Methods: Please update your search to the present time, as we require that SRs are updated to within roughly 6 months of the expected publication date and your search has not been updated since June 2019.*

**We updated the search to 19 November 2019. As discussed in the Cover Letter, this study the updated research resulted in the addition of one study [1], which reported one eligible comparison but was excluded due to extreme OR related to quasi-separation. Please see additional information in our response to Reviewer #2.**

*8. Results: Please provide both 95% CIs and p values for the analysis of exposure to suicide and association with increased suicide, suicide attempt, and suicide ideation (Lines 275-277).*

**We apologise for this omission. The Results (ln 348-52) now read:**

*Results from the subgroup analysis showed that exposure to suicide was associated with increased odds of suicide (k=24, OR=3.23, CI=2.32 to 4.51, P<0.001) and suicide attempt (k=15, OR=2.91, CI=2.01 to 4.23, P<0.001). However, there was no evidence of an association with suicidal ideation (k=3, OR=1.85, CI=0.97 to 3.51,P=0.06; Q between subgroups=2.31, df=2, P=0.32,* $R_{(2)}^{2}$*=11.77%,* $R_{(3)}^{2}$*=0%).*

*9. Discussion: Conclusions: Line 452-425: Please revise the first sentence of the conclusion paragraph to: “Our findings suggest that prior exposure to suicide is associated with increased risk of suicide and suicide attempt.” or similar.*

**The first sentence of the conclusion (ln 605) now reads:**

*Our findings suggest that prior exposure to suicide is associated with increased risk of suicide and suicide attempt.*

*10. Figure 2, Figure 3, Figure 4:  Please provide p values associated with the 95% CIs and odds ratios presented.  Please define the abbreviation “OR” and “CI”  in the figure legend.  Please indicate in the figure legend the meaning of the bars and markers.*

**We provided the Z- and P-values associated with each subgroup and overall analysis, along with heterogeneity estimates for the overall models.**

**The abbreviations have been defined.**

*11.Table 2, Table 3: Please define the abbreviation “CI” in the table legend.*

**All column headings have been amended to:**

*Odds Ratio (95% Confidence Interval)*

*12. S6 Figure and S7 Figure: One of the figures appears to have been duplicated here, please update with the correct figures.*

**We apologise for this error. The figures are now correct.**

*13. S6, S7, and S8 Figures: Please define the dashed and solid lines in the funnel plots in the figure legends.  Please define the abbreviation “OR” in the figure legends.*

Response

**These legends now read:**

*Note: LogOR = Log odds ratio. The solid vertical lines indicate the 95% confidence interval around the log odds ratio. The dashed lines indicate log odds ratio±1.96 standard error of the log odds ratio. The resulting triangular region indicates the expected location of 95% of studies in the absence of small study effect.*

*14. S9 Figure: What is the rationale for including the results of moderator analyses for exposure to suicide and suicide attempt as main manuscript tables, while the moderator analyses for exposure to composite measures of suicidal behavior is included in the supporting information?  Please include these data in the main manuscript unless there is a reason not to do so.*

**We assume this comment refers to S9 Table in our previous submission. These data are now included in the main manuscript as Table 4.**

*15. Checklist: Thank you for completing and including the PRISMA Checklist (S10 Text).  Please revise the checklist using section and paragraph numbers, rather than page numbers, to note the location of the checklist items.*

**The updated checklist has been provided as S9 Text PRISMA Checklist**

**Reviewer #1**

*Although this study has been conducted thoroughly, my main concern lies on pooling together rates from observation studies. It is well known that observational studies are susceptible to selection bias and their results are most of the times influenced by several confounding variables. The authors tried to adjust for confounders but there might be many more factors that have not been taking into account when pooling the rates across the different studies. Moreover, the gold standard approach in examining moderators at a meta-analytic level is the individual patient data meta-analysis. Study level meta-analysis rely on average effects and cannot examine individual participant differences. Thus, the examination of moderators is very limited in a conventional meta-analysis.*

**We agree that once we have identified study-level association and examined key moderators, the natural next step would be to conduct an individual participant data meta-analysis (IPDMA) aiming to examine individual-level moderators. We have therefore strengthened this point in the Discussion (ln 555-9):**

*Understanding these factors is important for identifying specifically, who within in the general population is most at risk. However, the pooling of observation studies meant that analyses of these factors were outside the scope of the present study. An important next step forward would therefore be examinations of exposure to suicide while taking these risk factors into account using individual participant data meta-analyses.*

*Moreover, I have some additional comments related to heterogeneity:*

*(a) in many outcomes, heterogeneity is surprisingly "low" given the nature of the included studies. Nevertheless, one cannot fully grasp the magnitude of heterogeneity based solely on the point estimate of I-square. The authors should calculate and report 95% CI around I-square to allow a better understanding of the heterogeneity in their meta-analyses.*

**We provided 95% CI estimates around the I-square statistic (**$\boldsymbol{I}_{\mathbf{(2)}}^{\mathbf{2}}$ **and** $\boldsymbol{I}_{\mathbf{(3)}}^{\mathbf{2}}$**) for the overall analyses of exposure to suicide and suicide attempt in the text and Figs 2&3, based on the test-based method proposed by Higgins & Thompson [2] and implemented in metaSEM. We refer to this interval as CI for clarity but note that uncertainty intervals might be a more appropriate term [3]. Please note that due to a very low power it was unfeasible to provide a reliable CI estimation for *I^2^* of exposure to suicidal behaviour [4].**

*(b) the authors reported a very high heterogeneity (87%) across 13 studies examining exposure to suicide attempt. Such heterogeneity implies a great diversity among the examined studies, making the use of meta-analytic methodology questionable. Did the authors explore sources of this heterogeneity? How do they interpret it?*

**We agree that between-study (i.e., level 3) heterogeneity was high for exposure to suicide attempt. However, we share the view that statistical heterogeneity alone cannot guide the decision of whether to pool data, and in fact can often be seen as an opportunity to study potential effect moderators, which is one of the main advantages of a meta-analysis [5]. To this end, we examined and present moderator (Maximum likelihood mixed-effects) analyses of 8 pre-registered design factors, along with R^2^ estimations at each level. These results are provided in Tables 2-4.**

**Moreover, throughout the Discussion we provide interpretations of key findings, either as factors that should be considered in future trial design or as caveats against over-interpretation of our results. For example, under Limitations we note (ln 562-9):**

*By contrast, study design characteristics accounted for 72.8% of between-study heterogeneity (*$I_{(3)}^{2}$*=87.8%) following exposure to suicide attempt. In this instance, cross-sectional studies reported significantly larger odds ratios (OR=8.23) compared to case-control (OR=2.74) and cohort (OR=2.69) studies. In general, cross-sectional studies are prone to an inherently greater number of biases, compared to case-control and cohort studies.*

**Reviewer #2**

***Page 6***	*Here the authors refer to ‘multilevel (three–level)’. I wonder whether in fact the term multilevel might be preferred in the title as the more general term? I have reviewed many studies which could have beneﬁted from a multilevel meta–analysis but can count on the ﬁngers of one hand those which actually did it. Making it easy to ﬁnd the more generic term might help others to ﬁnd this study as an example of how it is done.*

**We agree that the general term might be more useful for the reader and replaced the term ‘three-level’ with ‘multilevel’ throughout.**

***Page 10*** *I am not sure that the arbitrary cut off’s for heterogeneity are really helpful. In epidemiological studies high levels of heterogeneity are to be expected and the important thing is to describe it and, if possible, say why it is there.*

**We have removed the arbitrary cut offs from the Methods and have also avoided narrative descriptions of I^2^ values in the Results.**

***Page 13*** *I have to say that I would normally object quite strongly to excluding outliers but an odds ratio of 36.4 is so extreme that I can see the authors’ point here. I suppose we have no way of knowing what happened there? Extreme odds ratios are often due to separation or quasi–separation.*

**This was indeed a case of quasi-separation in a case-control study based on psychological autopsies. For the exact same reason we have excluded another study from the updated search with an OR of 18.1 (95% CI 1 to 329), once again due to 7/40 exposures among cases vs 0/40 among controls** **[1].**

***Page 13*** *I think there is a (k = ??) missing after the phrase ‘suicide attempt’ in one place.*

**We apologise for this omission and corrected it.**

***Page 25*** *Given the problems in detecting small study effects in the presence of heterogeneity I would hesitate to read too much into p =0.06 here.*

**We agree and therefore did not proceed beyond reporting this result.**

***Page 25*** *‘[. . . ] not suicidal ideation [. . . ] CI = 0.97’. Well, that is strictly true but I think this is slightly too strong. The authors found no evidence of association but the estimate is quite imprecise. My comment also applies to the same result in the abstract and to other results about ideation later. How about ‘there was no evidence of an association with suicidal ideation’?*

**We thank you for this comment and have modified the manuscript accordingly. For example:**

*However, no evidence of an association was observed for suicidal ideation outcomes (2 studies, N=43,354; OR=1.85, 95%CI=0.97 to 3.51, P=0.06).* **(ln 32-3)**

*However, there was no evidence of an association with suicidal ideation (k=3, OR=1.85, CI=0.97 to 3.51, P=0.06; Q-between subgroups=2.22, df=2, P=0.33,* $R_{(2)}^{2}$*=11.8%,* $R_{(3)}^{2}$*=0%).* **(ln 350-2)**

***Supplementary S2*** *Typo, ¿ instead of >*

**We corrected this typo.**

***Supplementary S3*** *That is a very impressive and helpful list of studies consulted.*

**Thank you. We have updated the list to include an additional 48 full text articles that were screened during the 19 November search update.**

***Supplementary S6*** *Unfortunately the ﬁle contained a duplicate of Figure S7.*

**We apologise for this error. The correct file has been uploaded.**

***Age***	*The problem with the way age is incorporated into the analysis is that the coverage of studies overlaps substantially and many of the ones labelled adult also include youths and vice versa. So the comment about age on page 25 may reﬂect the imprecision in the classiﬁcation of the moderator variable. I think I might mention this as a limitation. This is of course an ecological analysis and so perhaps less crucial.*

**We fully agree and removed this comment from the Results. In addition, we provide more context to the lack of evidence for age effects under the Limitations (ln 570-5):**

*It is noteworthy that we did not find evidence to support the role of age as a risk moderator suggested in previous reviews. Yet these results should be interpreted with caution, as the dichotomization of youth and adults across included studies were not mutually exclusive and included both adult and youth populations in 13 out of 34 studies. These findings may therefore be an artefact introduced by the imprecise age classification of the included population in individual studies.*

***Multiple exposure***	*On reading that 21% have experienced suicide in someone known to them the statistical reader immediately thinks that, conditional on independence, that means about 4.4% have experienced it twice, and indeed about 1% three or more times. There is a brief mention on page 36 of this. Would it be helpful if there are studies which have examined this to mention which they are and perhaps give a narrative summary?*

**We thank you for the suggestion and have updated the discussion (ln 535-45):**

*For example, a previous systematic review on pre-and post-loss features of suicide bereavement in young people found evidence of a cumulative effect of exposure to suicide on subsequent suicide risk. In the present meta-analysis two out of thirty-four studies included in our analyses provided separate estimates for multiple exposures to suicide and suicide attempt. In one study, exposure to two or more suicide deaths affected less than 1% of the population, but was associated with 9.8 greater odds of suicide attempt compared to an odds ratio of 3.8 among those who had been exposed to the suicide of one relative. Similarly, those exposed to the suicide attempt of two parents were 5.67 times more likely to make a suicide attempt, compared to an odds ratio of 2.89-3.89 (for paternal and maternal exposures, respectively) among youth who had been exposed to the suicide attempt of one parent.*

**Reviewer #3**

*First, it is noteworthy that exposure to suicide/suicide attempt in relatives vs. non-relatives produce similar odds ratios. This pattern suggests that the mechanism of increased risk for suicidal behavior is likely environmental more than genetic. That said, there was a tendency for the relatives odds ratio to be modestly higher than for non-relatives; if the authors think this difference is meaningful (it appears to be on the margins of statistical reliability), then perhaps the data suggest a small role for genetic transmission of risk in addition to environment.*

**Genetic transmission is a plausible contributor to the overall familial risk identified in our study. However, since families typically also share similar environmental factors, studies included in our meta-analysis were unable to delineate genetic from environmental factors.**

**Our results (Tables 2-4) do not indicate a difference between the two types of exposures, as indicated by the overlap between the two confidence intervals and weak indications of between-subgroup heterogeneity. Our Discussion now refers this issue as an arena for further study (ln 547-59):**

*For example, we did not find evidence of a significant difference in suicide-related outcomes when the exposure to suicide or suicide attempt occurred in relatives compared to friends and acquaintances. Although previous registry-based studies have shown a six-fold increase of suicide among biological relatives of adoptees who have died by suicide, in the present meta-analysis it was not possible to delineate between relatives who resided in the same household and therefore shared many of the same environmental risk factors. Understanding these factors is important for identifying specifically, who within in the general population is most at risk. However, the pooling of observation studies meant that analyses of these factors were outside the scope of the present study.*

*Second, it is noteworthy that exposure increases risk for suicidal behavior but not attempts. This pattern is consistent with recent suicide theory emphasizing a role for suicide capability in the transition from suicidal thoughts and behavior. For example the Three Step Theory (Klonsky & May, 2015) suggests that pain and hopelessness drive suicidal ideation, but capability for suicide (including acquired and practical contributors to capability) increase one's ability to act on suicidal ideation and make an actual suicide attempt. From this perspective, it is significant that exposure increases risk for behavior rather than ideation. Most people with suicidal thoughts do not make an attempt. For most people, even people with suicidal ideation, the idea of attempting suicide is cognitive difficult (is this really something i could do?) as well as practically difficult (how would i do it? what if my attempt went wrong? what if it's too scary?). Being exposed to someone who*

*has died by suicide can make the act of suicide seem more plausible, more cognitively feasible. In addition, knowing the method of the person's attempt can increase practical capability, in that the person exposed to the attempt now has concrete information about a method that resulted in suicide death. Thus, from the perspective of the Three Step Theory, exposure to suicide increases capability for suicide which, in turn, increases risk of suicidal behavior (but not suicide ideation).*

**We assume this comment refers to the inconclusive evidence regarding association with suicidal ideation and highly appreciate this very interesting interpretation of our results. In light of Reviewer #2’s comment on the same analysis, we believe that the best course of action would be to raise this extremely important issue while not suggesting that our results provide robust evidence either way. Thus, we have added an additional paragraph which explores our findings in the context of their limitations and existing theory of suicidality (ln 474-85):**

*Evidence from two studies suggest that exposure to suicide may be associated with increased risk of suicidal ideation, especially in older adults. Conversely, results from a single cohort study in youth indicate higher risk for suicide attempt than suicidal ideation, pointing once more to lack of uniformity across populations and outcomes. Moreover, theoretical and empirical accounts suggest that while exposure to suicide may contribute to subsequent suicidal ideation to some extent, its effect on people with a history of suicidal ideation may be more pronounced, as this experience might reduce cognitive and practical barriers to acting on one’s suicidal thoughts. A more comprehensive look at this interaction may have important practical implications for developing specific interventions for this high-risk population, in particular those guided by the ‘ideation to action framework’ that aim to reduce acquired capability for suicidal behavior among individuals exposed to suicide.*

**REFERENCES**

1.	Rasouli N, Malakouti SK, Rezaeian M, Saberi SM, Nojomi M, De Leo D, et al. Risk Factors of Suicide Death Based on Psychological Autopsy Method; a Case-Control Study. Arch Acad Emerg Med. 2019;7(1):e50. Epub 2019/10/12. PubMed PMID: 31602433; PubMed Central PMCID: PMCPMC6785207.

2.	Higgins JP, Thompson SG. Quantifying heterogeneity in a meta-analysis. Stat Med. 2002;21(11):1539-58. doi: 10.1002/sim.1186. PubMed PMID: 12111919.

3.	Higgins JP, Thompson SG, Deeks JJ, Altman DG. Measuring inconsistency in meta-analyses. BMJ. 2003;327(7414):557-60. doi: 10.1136/bmj.327.7414.557. PubMed PMID: 12958120; PubMed Central PMCID: PMC192859.

4.	Huedo-Medina TB, Sanchez-Meca J, Marin-Martinez F, Botella J. Assessing heterogeneity in meta-analysis: Q statistic or I2 index? Psychol Methods. 2006;11(2):193-206. Epub 2006/06/21. doi: 10.1037/1082-989X.11.2.193. PubMed PMID: 16784338.

5.	Ioannidis JP, Patsopoulos NA, Rothstein HR. Reasons or excuses for avoiding meta-analysis in forest plots. BMJ. 2008;336(7658):1413-5. doi: 10.1136/bmj.a117. PubMed PMID: 18566080; PubMed Central PMCID: PMCPMC2432114.
