# Supplementary material for: Association of suicidal behavior with exposure to suicide and suicide attempt: A systematic review and multilevel meta-analysis
Source: PLoS Med. 2020 Mar 31;17(3):e1003074. doi: 10.1371/journal.pmed.1003074 (PMC7108695; doi:10.1371/journal.pmed.1003074)
Supplement: Attachment — Submitted filename: Response to reviewers.docx [file pmed.1003074.s013.docx]

**Requests from Editors:**

1. *The abstract could be trimmed – perhaps the methods and findings section, mostly.*

**The abstract has been revised accordingly.**

2. *Thank you for providing an author summary – it should be in bulleted format for house style, please (the text of the points is fine, just please add bullets).*

**The author summary has been revised accordingly.**

3. *Line 293 Studies were most commonly rated fair (13/34), followed by good (13/34) and poor – is this correct? Fair and good have the same rating? Just checking, apologie s if I’ve misunderstood.*

**The numbers are correct. However the section has been updated (ln 326) to:**

*“Studies were most commonly rated fair (13/34) and good (13/34), followed by poor (8/34, S3 Table).”*

**The discussion has also been updated (ln 386) to:**

*“Based on findings from 34 studies of mostly good and fair quality”*

4. *Line 505 – please just say association instead of evidence of association*

**The section has been revised accordingly.**

5. *Line 36 – limitations – Please note study quality, and/or the possibility of unmeasured confounders influencing the findings.*

**The section has been updated to (ln 36):**

*“A key limitation of this study include fair study quality, and the possibility of unmeasured confounders influencing the findings.”*

6. *At line 40, suggest beginning the sentence with "The findings of this systematic review and meta-analysis  indicate that …*

**The section has been revised accordingly.**

7. *Please delete "2.99" at line 377 (or add "(OR=2.99)").*

**The section has been revised accordingly.**

8. *I wonder if "prior exposure to suicide" is somewhat ambiguous as it could be read to include exposure to media representations. Perhaps one might amend the wording to "direct exposure to suicide" early on in the paper.*

**We thank you for the suggestion. We have clarified the relationship of exposure to relatives and friends and acquaintances as we believe direct exposure may be potentially interpreted as having witnessed the suicide-related act. We have updated the Abstract (ln 14-17) to:**

*“Eligible studies included cohort, case-control, and cross-sectional designs reporting comparative data from prior exposure to suicide, suicide attempt, or composite measures of suicidal behavior on the outcomes of suicide, suicide attempt and suicidal ideation in relatives, friends and acquaintances.”*

***We have also updated the Introduction (ln 134-37) to:***

*We therefore aimed to conduct a systematic review and multilevel meta-analysis investigating the independent association between prior exposure to suicide, suicide attempt and composite measures of suicidal behavior on subsequent suicide, suicide attempt, and suicidal ideation in relatives, friends and acquaintances.*

**FIGURES and GRAPHICS:**

1. Please remove embedded figures from your manuscript file and

**The sections have been revised accordingly.**

2. Ensure the most up to date versions of your figures are uploaded to our submission system as separate TIF or EPS files. Please do not remove the figure legends and captions.

**The sections have been submitted accordingly.**

3. Please ensure that all main figure files are cited in ascending numerical order in the main text of the article.

**The sections have been revised accordingly.**

4. Please ensure that all main figures are referenced in the manuscript as Fig 1, Fig 2, etc. (including capitalization), rather than Figure 1, Figure 2, etc. Please note, however, that the file names themselves must not include the space, i.e. Fig1.tif, Fig2.tif, etc.

**The sections have been revised accordingly.**

5. Please ensure that all main figure legends are placed after the paragraph containing their first in-text citation.

**The sections have been revised accordingly.**

**TABLES and BOXES:**

1. Please cite main tables in ascending numerical order in your main article file.

**The sections have been revised accordingly.**

2. Please ensure that all main tables in their entirety (including titles and footnotes), are placed after the paragraph containing their first in-text citation.

**The sections have been revised accordingly.**

3. Tables 1, 2, 3 and 4 use returns to control text wrapping. This is indicated by a [¶  or ↵] symbol when "Show Paragraph Marks" is turned on in Word. Please either split those cells into separate cells to achieve alignment or remove the returns.

**The tables have been revised accordingly.**

    METADATA:

1. In accordance with PLOS' data policy, please ensure that your Data Availability Statement in the submission form clearly identifies how readers can access your data. Data cannot be available on request, or only accessible by contacting one of the authors. Note that your Data Availability Statement will be typeset as it is written, so please ensure it is in complete sentences and appears as you would like it to in the published version.

**The Data Availability Statement has been revised accordingly.**

**ARTICLE FILE:**

1. Please indicate the corresponding author in the byline by placing an asterisk (*) after their affiliation number.

**The section has been revised accordingly.**

2. Please include the corresponding author's email address on the title page of your manuscript, indicated by an asterisk (*). Only an asterisk and the email address itself are required.

**The section has been revised accordingly.**

3. Please ensure that all authors have at least one affiliation listed on the title page, and that all listed affiliations are attributed to at least one author. Affiliations should be listed in ascending numerical order in both the affiliation list and in the author byline.

**The section has been revised accordingly.**

4. The sections in your manuscript do not comply with PLOS requirements. Please rename your headings as follows (ensuring you use them in the same order): Abstract, Introduction, Methods (or Methods and Materials), Results, Discussion.

**The sections have been revised accordingly.**

5. Please remove the funding information from your manuscript file. The published version is pulled automatically from the “Financial Disclosure” field in the submission form and must not be duplicated. Please therefore take this opportunity to check that the information in this field is correct and completed in full.

**The section has been revised accordingly.**

6. Please remove the Competing Interests section from your manuscript file. The published version is pulled automatically from the "Competing Interests" field in the submission form and must not be duplicated. Please therefore take this opportunity to check that the information in this field is correct and completed in full.

**The section has been revised accordingly.**

7. Please remove your Author Contributions section from your manuscript file as this information is automatically pulled from the authors section of our submission form and must not be duplicated. Please therefore take this opportunity to check that the information in this field is correct and completed in full.

**The section has been revised accordingly.**

8. Please remove the data availability information from your manuscript file as this is automatically pulled from the "Data Availability" field in the submission form and must not be duplicated. Please therefore take this opportunity to check that the information in this field is correct and completed in full.

**The section has been revised accordingly.**

**REFERENCES:**

1. Please ensure your references are in the style of PLOS Medicine (ICMJE/Vancouver style). The reference style guide can be found here: <http://journals.plos.org/plosmedicine/s/submission-guidelines#loc-references>. You can also find a downloadable EndNote file in these guidelines to assist you. Please do a manual check as well, as the EndNote file does not catch everything. Please note that the references will be typeset as they appear so please ensure the format is consistent.

2. Please put all reference citations in square brackets separated by either commas or dashes with no spaces, e.g. [1,2,3] or [1-3]

**The references have been revised accordingly.**

    3. Please make sure all references are cited in ascending numerical order in the text.

**The references have been revised accordingly.**

**SUPPORTING INFORMATION:**

1. Please upload your supporting information as individual files, and use the following naming format: S1 Text; S1 Fig; S1 Table, S1 Data, etc. Each file type should start as S1 and increase numerically: S1 Text, S2 Text, etc. Note that all relevant in-text citations and captions will need to be updated accordingly.

Complete

**The sections have been revised accordingly.**

2. Please ensure that all Supporting Information files use the following format verbatim throughout the manuscript (including legends and all in-text citations): S1 Fig, S1 Table, S1 Text, S1 Data, etc. Each file type should start at 1 and increase numerically.

**The sections have been revised accordingly.**

3. Please note, however, that the file names themselves must use an underscore rather than a space, i.e. S1_Fig.tif, S1_Table.xlsx, S1_Text.doc, S1_Data.xlsx, S1_PRISMA_Checklist.docx, etc.

**The file names have been revised accordingly.**

4. Please ensure that each supporting information file has a legend at the end of the manuscript file, after the Reference list.

**The sections have been revised accordingly.**

5. Please note that your submission cannot contain any references to generic "supporting information" files and instead must refer to specific files using our naming convention (S1 Fig, S1 Table, S1 Text, S1 Data, SI PRISMA Checklist, etc.)

**The sections have been revised accordingly.**
